# UGT8 mediated sulfatide synthesis modulates BAX localization and dictates apoptosis sensitivity of colorectal cancer

**DOI:** 10.1038/s41418-024-01418-y

**Published:** 2024-11-23

**Authors:** Le Zhang, Prashanthi Ramesh, Lidia Atencia Taboada, Rebecca Roessler, Dick W. Zijlmans, Michiel Vermeulen, Daisy I. Picavet-Havik, Nicole N. van der Wel, Frédéric M. Vaz, Jan Paul Medema

**Affiliations:** 1 LEXOR, Center for Experimental Molecular Medicine, Cancer Center Amsterdam, Amsterdam UMC, University of Amsterdam, Amsterdam, The Netherlands; 2 Oncode Institute, Amsterdam, The Netherlands; 3 Department of Molecular Biology, Faculty of Science, Radboud Institute for Molecular Life Sciences, Radboud University Nijmegen, Nijmegen, The Netherlands; 4 Oncode Institute, Nijmegen, The Netherlands; 5 Division of Molecular Genetics, The Netherlands Cancer Institute, Amsterdam, The Netherlands; 6 Medical Biology - MB Core Facility, Amsterdam UMC, University of Amsterdam, Amsterdam, The Netherlands; 7 Amsterdam UMC location University of Amsterdam, Department of Laboratory Medicine and Pediatrics, Laboratory Genetic Metabolic Diseases, Emma Children’s Hospital, Amsterdam, The Netherlands; 8 Amsterdam Gastroenterology Endocrinology Metabolism, Inborn errors of metabolism, Amsterdam, The Netherlands; 9 Core Facility Metabolomics, Amsterdam UMC location University of Amsterdam, Amsterdam, The Netherlands

**Keywords:** Cancer metabolism, Sphingolipids, Cell biology

## Abstract

Elevated de novo lipid synthesis is a remarkable adaptation of cancer cells that can be exploited for therapy. However, the role of altered lipid metabolism in the regulation of apoptosis is still poorly understood. Using thermal proteome profiling, we identified Manidipine-2HCl, targeting UGT8, a key enzyme in the synthesis of sulfatides. In agreement, lipidomic analysis indicated that sulfatides are strongly reduced in colorectal cancer cells upon treatment with Manidipine-2HCl. Intriguingly, this reduction led to severe mitochondrial swelling and a strong synergism with BH3 mimetics targeting BCL-XL, leading to the activation of mitochondria-dependent apoptosis. Mechanistically, Manidipine-2HCl enhanced mitochondrial BAX localization in a sulfatide-dependent fashion, facilitating its activation by BH3 mimetics. In conclusion, our data indicates that UGT8 mediated synthesis of sulfatides controls mitochondrial homeostasis and BAX localization, dictating apoptosis sensitivity of colorectal cancer cells.

## Introduction

Colorectal cancer (CRC) is one of the most common and best-studied cancers worldwide. Despite therapy innovation, many CRC patients, especially at late-stage disease, still have a poor prognosis and display unresponsiveness to current therapies [1, 2]. Novel therapeutic modalities are therefore still urgently needed. However, during progression, cancer cells are shaped to withstand stresses, such as oncogene-induced stress, hypoxia, nutrient insufficiency and anti-cancer immune attack. All these stresses are reported to somehow trigger the apoptotic machinery, which is wired to restrain the development of cancer. Effective progression of cancer cells therefore inevitably aligns with an adaptation to survive stresses and apoptotic insults, for instance through the upregulation of anti-apoptotic BCL2 family members. This adaptation endows cancer cells with apoptosis resistance. In CRC, we and others have shown, that a switch towards a dependency on BCL-XL, one of the anti-apoptotic BCL2 proteins, for survival is absolutely required for progression [3, 4]. In agreement, CRC can be effectively targeted with BH3 mimetics that antagonize the activity of BCL-XL. BH3 mimetics act as apoptosis inducers by modulating the balance between anti-apoptotic BCL2 proteins and apoptotic executioners like BAX. When relieved of inhibition, BAX is set free to oligomerize and permeabilize the outer mitochondrial membrane (OMM), releasing cytochrome c and unleashing the downstream apoptotic machinery [5].

Another way cancer cells adapt to changing requirements and stress is by a complete rewiring of their metabolic pathways. Almost 100 years ago, Warburg proposed the glycolytic adaptation of cancer cells [6], but a much broader metabolic adaptation of multiple pathways is detected and in fact constitutes one of the hallmarks of cancer [7]. Indeed, cancer cells acquire the capacity for de novo fatty acid synthesis and change their lipid metabolism to fit a growing need for fatty acids and lipids [8]. Intriguingly, this also exposes vulnerabilities and several studies have used drugs to target lipid metabolism as a potential and promising approach in cancer treatment [9–13]. Whether the metabolic adaptation is also linked to escape from stress and apoptosis is not fully explored. However, multiple lipid species have been reported to regulate the onset and execution of apoptosis [14]. For instance, cardiolipin is a crucial player in mitochondrial apoptosis and its constitution is adapted in cancer cells [15, 16]. Similarly, sphingolipids, which form a subgroup of lipids enriched in membrane micro-domains also known as lipid rafts [17], play a role in orchestration of signal transduction but also regulate apoptosis [18]. The metabolism of sphingolipids is complex and split into many connected pathways, with ceramides located centrally to many of these metabolic pathways. De novo synthesis, salvage pathways, sphingomyelin synthesis as well as glycosylation gives rise to the diversity of a sphingolipid superfamily [19]. Although some sphingolipids have been reported to regulate mitochondrial apoptosis [18, 20], their exact role remains largely unclear.

Here, we aimed to identify sensitizers of CRC cells to low doses of the BCL-XL inhibitor, A-1155463, in order to exploit the vulnerability towards BCL-XL inhibition of these cancer cells. We revealed that Manidipine-2HCl decreased the activity of UDP-glycosyltransferase 8 (UTG8) and as a result lowered sulfatide levels. This strongly affected mitochondrial function, reflected by a drop in respiration, mitochondrial swelling as well as enhanced mitochondrial BAX localization, which facilitated BH3 mimetic-induced apoptosis. Our data reveal a novel regulatory level of mitochondrial-dependent apoptosis that can be exploited to kill CRC cells.

## Materials and methods

### Compounds and reagents

A-1155463, Manidipine-2HCl, Q-VD-Ohe, ABT-199, AZD5991 and Clevidipine, Lacidipine, Nilvadipine, Nitredipine were purchased from Selleckchem. UGT8i19 was purchased from Cayman Chemical and sulfatides (Brain, Porcine) were the product of Avanti Polar Lipids. Ionomycin from *Streptomyces conglobatus* and Antimycin A was a product of Sigma. FCCP and Mitoquinone (MitoQ) mesylate was purchased from MedChemExpress. All these compounds were dissolved in DMSO and stored at –80 °C.

### Cell and organoid culture

All primary colon cancer cell lines (Co01, Co108, Co147) were maintained in Poly-HEMA coated repellent flasks in Advanced DMEM/F12 (Gibco) supplemented with N2 supplement (Gibco), 8.5 mM glucose (Sigma-Aldrich), 2mM L-glutamine (Lonza), 25μM HEPES (Thermo Fisher Scientific), 100μM β-mercaptoethanol (Sigma-Aldrich), 1:1000 Trace supplement B and C (Thermo Fisher Scientific), 2 μg/ml Heparine (Sigma-Aldrich), 10 μg/ml Insulin (Sigma-Aldrich), 50 ng/ml Human EGF (Peprotech) and 4 ng/ml Human bFGF (Tebu-Bio). All primary lines are grown in suspension and for passaging are disassociated with either trypsin(100 mg/L)/EDTA(55 mg/L) (Capricorn Scientific) for Co01 or 1× accutase (400–600 μ/ml Sigma) for the others. CRC cell lines RKO, SW48, HT55, LS-180, HUTU-80 and OMUS-23 were cultured in DMEM/F12 (Gibco) plus 10% Fetal Calf Serum (FCS, Gibco) and 50 U/ml penicillin-streptomycin (Gibco), while HCT-116, LS-1034, RCM-1, SNU-C1 and NCI-H716 were culture in RPMI 1640 plus glucose, pyruvate, L-glutamine and 10% FCS (Gibco) and 50 μ/ml penicillin-streptomycin (Gibco). Patient-derived tumor organoids P9T, P16T and normal colon organoids were maintained in drops of growth factor-reduced Matrigel (Corning). P9T and P16T organoids were maintained in Advance DMEM/F12 supplemented with N2 supplement, B27 (Gibco), antimycotic/antibiotic (Thermo Fisher Scientific), gentamicin (Thermo Fisher Scientific), 2 mM GlutaMax-1 (Thermo Fisher Scientific), 10 mM HEPES, 1mM N-acetyl-l-cysteine (Sigma-Aldrich), 10 nM [Leu15]-gastrin I (Sigma-Aldrich), 10 mM nicotinamide (Sigma-Aldrich), 500 nM A83-01 (Tocris), 3 μM SB202190 (Sigma-Aldrich), 50 ng/mL human EGF, 20% RSPO1 conditioned medium, 10% Noggin conditioned medium, and 10 nM PGE2 (Santa Cruz Biotechnology). Human normal colon organoids Co119N used for matrix titration were maintained in IntestiCult™ Organoid Growth Medium (Human, StemCell technologies). Human healthy platelets were isolated from peripheral blood by centrifuge. In brief, platelet-enriched plasma was obtained by centrifuging the whole blood from healthy donor collected in sodium citrate tubes at 180 × *g* for 15 min. 90 µl platelet enrich plasma was plated in each well of 96 well plates and treated with compounds for 24 h before the viability was determined by CellTiter Blue assay. All cell lines were verified by STR profiling at Center for Experimental Molecular Medicine, Amsterdam UMC and regularly tested negative for mycoplasma.

### Generation of cell lines

#### Co01-Mito-DsRed and Co01-Mito-Car-GECO

Mito-Car-GECO is a mitochondria-targeting Ca^2+^ probe. This construct was created as described in ref. [21]. Co01 expressing Mito-DsRed or Mito-Car-GECO were established by lentiviral transduction. In brief, lentivirus particles were packaged by co-transfecting HEK-293T cells with 5.6 μg pLV-mitoDsRed or pLenti-Mito-Car-GECO, 4 μg pPAX2 and 1.3 μg pMD2G plasmids. pLV-mitoDsRed was a gift from Pantelis Tsoulfas (Addgene plasmid # 44386; http://n2t.net/addgene:44386; RRID:Addgene_44386). pLenti-Mito-Car-GECO was a gift from Joseph Gordon (Addgene plasmid # 100765; http://n2t.net/addgene:100765; RRID:Addgene_100765). Cells were transduced by mild centrifuging at 1500 rpm at 32 °C for 1 h. 48 h after transduction, DsRed or GECO positive cells were sorted out by Sony SH800 Cell Sorter.

#### Co01-BAX/BAK and UGT8 Knock-out

To knock-out BAX/BAK and UGT8 in the Co01 cell line, lentivirus-based gRNA/Cas9 vector lentiCRISPRv2 was used to generate the construct targeting BAX or BAK with the following guide RNA. BAX: CCATTCGCCCTGCTCGATCC; BAK: (1) GCATGAAGTCGACCACGAAG, (2) GTTGATGTCGTCCCCGATGA; UGT8: GAGTGCTGTTGGGATAGCGA. Lentivirus containing above-described constructs were produced by the same packaging procedures as described in producing pLV-mitoDsRed. Co01 cells were transduced as described before and selected with 2 μg/ml puromycin for 72 h before single cell sorting was performed with Sony SH800 Cell Sorter to generate clones from single cells. BAX (Co01-BAX-KO) or BAK (Co01-BAK-KO) knock-out clones were validated by Western blot, while UGT8 knockout were quantified by TIDE analysis [22]. BAX/BAK double knock-out line (Co01-DKO) was established by transducing Co01-BAK knock-out line with BAX targeting lentiCRISPRv2 and single clones were validated and selected by Western blot.

#### Co01-BAX-KO-CFP-BAX

To create Co01 cell expressing CFP-BAX, transduction of the Co01-BAX-KO was performed. A lentivirus-based expression vector containing the human BAX ORF in frame with CFP was generated by Twist Bioscience. To avoid cutting by cas9 guided by RNA targeting wild-type BAX, which is present in the Co01-BAX-KO line, the PAM and BAX gRNA binding sequences of the BAX ORF were mutated without affecting the protein sequence. Lentivirus containing the construct were produced as described before. Co01-BAX-KO line was transduced and sorted by CFP to establish the Co01-BAX-KO-CFP-BAX line.

### Cell viability assay

Cell viability after drug treatment was determined by CellTiter blue assay. Briefly, cells were plated in 96 well plates and treated with the different drugs starting 24 h after plating, for an additional 48 h before CellTiter Blue (Promega) was added (20% volume). Cells were incubated for 3 h at 37 °C and color intensities were determined at 595 nm on a Biotek plate reader.

### Flow cytometry

#### Annexin V/Propidium Iodide staining

Annexin V/PI assays were performed to determine cell viability based on measuring translocation of phosphatidylserine (PS) and cell membrane integrity. Briefly, APC conjugated Annexin V (BioLegend, (1:20)) was used to label the PS on the external leaflet of apoptotic cells at room temperature for 15 min. Then 1 μg/ml Propidium Iodide (PI, Sigma) was used to stain the dead cells with compromised membranes. The positivity of each probe was detected 5 min later by flow cytometry.

#### Caspase 3 activation

Caspase 3 activation was determined by CaspaTag Caspase 3,7 In Situ Assay Kit, Sulforhodamine as instructed by the vendor (Merck). In brief, 50,000 cells were resuspended in 50 μl labeling solution and labeled at 37 °C for 60 min after which caspase-3 activity was detected by FACS.

#### Fluo-4 and mitotracker green staining

To assess the intracellular Ca2+ level, Fluo-4 (Thermo Fisher Scientific) was used to label the cells. Briefly, 50,000 cells were re-suspended in 50 μl labeling solution with 1 μM Fluo-4 and incubated at 37 °C for 30 min. Subsequently, fluorescence was determined by flow cytometry. Similarly, 50 nM mitotracker green (Thermo Fisher Scientific) was used to assess the mitochondrial mass. The fluorescence was measured after 30 min incubation at room temperature by Flow cytometry and the geometric mean of the fluorescence was generated by flowjo.10 (BD Biosciences).

#### Galactosyl-ceramide staining

To measure the expression of the galactosyl-ceramides (GalCer) in Co01, 10 μg/ml of anti-galactocerebroside antibody, clone mGalC (Merck, MAB342) was incubated with the cells in PBS with 1%BSA on ice for 30 min. After washing with PBS twice, 0.4 μg/ml of APC-conjugated Goat Anti-Mouse Ig (Multiple Adsorption, BD Pharmingen™, 550826) was added and incubated on ice for 30 min. Cells were washed twice with PBS and fluorescence was measured by Flow cytometry. The geometric mean of the fluorescence was analyzed by flowjo.10 (BD Biosciences) and plotted.

#### JC-1 staining

1 μM JC-1 (Thermo fisher) was added to cells treated with or without Manidipine and incubated at 37 °C for 30 min. FCCP was used as an OXPHOS uncoupler positive control. Two forms of JC-1 were detected with FACS at FL-1 (Mono) and FL-2 (Aggregates). The fluorescence was analyzed by flowjo.10 (BD Biosciences) and plotted.

#### Mitochondrial ROS measurement

Mitochondria ROS was measured by MitoSOX™ Mitochondrial Superoxide Indicators (Thermo Fisher). In brief, 50,000 cells were plated and treated with Manidipine for 24 h and labeled with 1 μM Mito SOX indicator for 30 min. Fluorescent intensity of the indicator was measured by flow cytometry. The geometric mean of the fluorescence was analyzed by flowjo.10 (BD Biosciences) and plotted.

### Quantitative PCR

The whole DNA from cell treated with or without Manidipine were extracted by the NucleoSpin®Tissue Kit (Bioke) as instructed by manufacturer’s manual. The mitochondria/nuclear DNA ratio was calculated by comparing the detection levels of the mitochondrial gene (MT-ND2) to the nuclear DNA element (AluYb8). Two sequences were amplified and quantified by Sybr Green (Roche) with the primers: (1)MT-ND2: forward: TGTTGGTTATACCCTTCCCGTACTA; reverse: CCTGCAAAGATGGTAGAGTAGATGA; (2)AluYb8: forward: CTTGCAGTGAGCCGAGATT; reverse: GAGACGGAGTCTCGCTCTGTC. Mitochondrial/nuclear DNA ratio was plotted.

### Western blot

Cells were pelleted and lysed on ice in RIPA Lysis and Extraction Buffer (Thermo Fisher Scientific) supplemented with 1× Halt™ Protease and Phosphatase Inhibitor Cocktail (Thermo Fisher Scientific) for 30 min. Cell debris was removed by centrifuging at 14,000 rpm for 10 min. Supernatants were collected and quantified by Pierce™ BCA Protein Assay Kits (Thermo Fisher Scientific). 20 µg total proteins was loaded per well of a 4–15% Mini-PROTEAN® TGX™ Precast Protein Gels (15-well, Bio-Rad) and separated for 90 min at 100 V using SDS-based electrophoresis. Proteins were then transferred to 0.2 µm PVDF membrane (Trans-Blot turbo RTA transfer kit PVDF, Bio-Rad) by Trans-Blot Turbo Transfer System (1 mini TGX gel turbo mode, Bio-Rad). Membrane was then blocked with 5% skim milk (Sigma) before incubation at 4 °C overnight with 1:1000 anti-BAX (2774S, Cell signaling), anti-BAK (3814S, Cell signaling) or 1:3000 anti-GAPDH (MAB374, 6C5, Merck). 1:5000 Goat Anti-Rabbit IgG(H+L), Mouse/Human ads-HRP (SouthernBiotech) or Goat Anti-Mouse IgG(H+L), Human ads-HRP (SouthernBiotech) were applied as secondary antibody at room temperature for 1 h before Lumi-Light plus western blot substrate (Roche) was used. Images of the blot were obtained with the ImageQuant™ LAS 4000 biomolecular imager (GE Healthcare).

### Digitonin assay for cytosolic fraction

To detect the formation of mitochondrial outer membrane permeabilization (MOMP), cytosolic fractions of treated cells were extracted by 50 µg/ml digitonin (Sigma) supplemented with 1× Halt™ Protease and Phosphatase Inhibitor Cocktail (Thermo Fisher Scientific) on ice for 5 min. The supernatants (cytosolic fraction) were collected and the pellets (contains mitochondrial fraction) were further homogenized with RIPA Lysis and Extraction Buffer supplemented with 1× Halt™ Protease and Phosphatase Inhibitor for 30 min. Cytochrome c in both fractions was blotted and detected by mouse anti human cytochrome c (1:500, 65981A (7H8), BD Biosciences).

### Isolation and preparation of heavy membrane fraction containing mitochondria

To determine the quantity of lipids, the heavy membrane (HM) fraction, which primarily consists of mitochondria was isolated with Mitochondria Isolation Kit for Cultured Cells (Thermo Fisher Scientific) as the manual instructed. In brief, CRC cell Co01 were pelleted and homogenized with Dounce homogenizer. The supernatants were collected after centrifuging at 700 × *g* for 10 min. The supernatants with organelles were further centrifuged at 3000 × *g* for 10 min to obtain mitochondria with less contamination of other organelles. The pellets of mitochondria were washed before used for in vitro cytochrome c release assay or sent to lipidomics.

### Cytochrome c release assay on HM fraction containing mitochondria

To investigate the direct effect of sulfatides on permeabilization of mitochondrial outer membrane, HM fraction containing mitochondria was pretreated with or without 25/50 µM sulfatides for 15 min to allow the incorporation of these lipids, followed by addition of 25 nM recombinant human BAX protein (provided by Raed Shalaby and Ana García Sáez, CECAD, Cologne, Germany) and 5 nM A-1155463 to permeabilize OMM at 37 °C for 1 h. Supernatants (released fraction) were collected by centrifuging at 5000 rpm for 10 min. The pellets (retained fraction) were further homogenized with RIPA Lysis and Extraction Buffer supplemented with 1× Halt™ Protease and Phosphatase Inhibitor for 30 min. Cytochrome c in both fractions was blotted and detected by mouse anti-human cytochrome c (1:500, 65981A (7H8), BD Biosciences). Grayscale values of the bands were obtained by ImageJ. The average relative release of cytochrome c was calculated and plotted according to three independent blots.

### Confocal fluorescent microscopy

#### Mitochondria imaging

To image the mitochondria by confocal microscope, 50,000 Co01 or Co01-Mito-DsRed cells in 500 μl medium were plated into 4 well Nunc™ Lab-Tek™ II Chamber Slide™ (Thermo Fisher Scientific) one day before treatment and then treated with drugs for another 24 h. Before imaging, mitochondria of Co01 were stained with 50 nM mitotracker Deep Red for 30 min. Nuclei were counterstained with Hoechst 33342. Mitochondrial images were captured at 63× oil magnification using a Leica TCS SP8 X. All the images were deconvoluted by Huygens professional software. The size, aspect ratio (AR, major axis/minor axis), and roundness of the particles of mitochondria were measured and calculated by ImageJ. As described in ref. [23], mitochondria were stratified into four groups based on the following criteria: (1) Networked: AR > 2.5; (2) Rod: AR ≤ 2.5, size≤1 × 10^–8 ^cm^2^, roundness≤0.8; (3) Punctate: AR ≤ 2.5, size≤1 × 10^–8 ^cm^2^, roundness>0.8; (4) Large round: size>1 × 10^–8 ^cm^2^. Distribution of each type of mitochondria in each group was plotted.

#### BAX aggregation imaging

Co01-BAX-KO-CFP-BAX cells were seeded into 4 well Nunc™ Lab-Tek™ II Chamber Slide™ one day before treatment. Cells were then treated with 2 μM Manidipine-2HCl or 2 μM UGT8i19 with or without 50 μM addition of exogenous sulfatides (Avanti Polar Lipids) to the medium for 24 h. On the day of imaging, cells were then treated with or without 5 nM A-1155463 for 3 h to trigger BAX aggregation. Right before imaging, mitotracker deep red was added to visualize mitochondria. All pictures were captured with a Leica TCS SP8 SMD. At least 3 pictures were taken for each condition for ImageJ quantification. To quantify BAX aggregation per cell, CFP channel pictures were analyzed by ImageJ particle analysis. Total area of fluorescent spots in each of the cells (A/cell) were quantified by the software, then average area of fluorescent spots per cell (A/cell) was calculated by averaging all the cells in one picture.

#### Active BAX staining and imaging

Co01-BAX-KO/CFP-BAX/Mito-DsRed cells were seeded on the round coverslips one day before treatment. Cells were then treated with 2 μM Manidipine-2HCl for 24 h. On the day of staining, cells were then treated with or without 5nM A-1155463 for 3 h to trigger BAX activation. Subsequently, cells were washed with PBS and fixed in 4% paraformaldehyde for 10 min. After washing with PBS, 1% Triton-X 100 was added to permeabilize the cells. Non-specific staining was blocked with 5% BSA and cells were subsequently incubated with 10 μg/ml Mouse Anti-BAX Clone 6A7 (RUO, BD Pharmingen™, 556467) at 4 °C overnight. After washing 1 μg/ml of APC-conjugated Goat Anti-Mouse Ig (Multiple Adsorption, BD Pharmingen™) was added and incubated at room temperature for 1 h. Cells were washed and images were captured at 63× oil magnification by Leica TCS SP8 X.

### Seahorse assay

All seahorse assays were performed according to the manufacturer’s instructions. Briefly, 16.000 cells were plated into Seahorse XF96 Cell Culture Microplates (Agilent) and cultured in complete advance DMEM/F12 medium containing 2% matrigel (Corning) to allow the cells to attach overnight. After 24 h treatment with the respective drugs, the oxygen consumption rate (OCR) were measured by Seahorse XFe96 Analyzer (Agilent) using the mito stress protocol. In brief, the basal OCR was determined followed by the first injection of Oligomycin (final concentration in wells was 1.5 μM) and then maximal OCR was read again after injection of FCCP (final concentration in wells was 1 μM). Rotenone (final concentration in wells was 1.25 μM) and antimycin A (final concentration in wells was 2.5 μM) were injected as the final step to determine baseline non-OXPHOS OCR. After the Seahorse run cells were lyzed with 1% NP-40 and the total protein was quantified by Pierce™ BCA Protein Assay Kits (Thermo Fisher Scientific). OCR readout was normalized to the corresponding BCA readout.

### Transmission electron microscope

To fix cells, the culture medium was mixed 1:1 with fresh 2x fixative (4% paraformaldehyde and 1% glutaraldehyde in PHEM buffer pre-warmed to room temperature). After incubation for 2 h at room temperature, fixed cells are stored in 0,5% paraformaldehyde. Then samples were washed and fixed with 1% OsO4 solution and dehydrated in a series of ethanol. After infiltration of the epon, pellets were polymerized at 65 °C. Ultra-thin sections were cut at a Leica Ultracut FC6, stained with Uranyl Acetate and imaged at the Transmission Electron Microscope (Talos 120 with Ceta 16M camera and Tecnai T12 with Velata and Xarosa camera). The average size of each mitochondrion was measured by imageJ and plotted. The percentage of mitochondria with damaged cristae was also calculated and plotted.

### Lipidomics

Lipidomics analysis was performed as previously described [24]. In short, a range of internal standards, dissolved in 1:1 (v/v) methanol:chloroform, were added to each sample. Subsequently, samples were centrifuged (10 min) at 14,000 rpm and the supernatant was evaporated under N2 at 60 °C. Lipid residue was dissolved in 150 μL of (v/v) methanol:chloroform (1:1v/v). Lipids were analyzed using a Thermo Scientific Ultimate 3000 binary HPLC coupled to a Q Exactive Plus Orbitrap mass spectrometer. Quantification was performed using the in house developed pipeline.

### Thermal proteome profiling

Thermal protein profiling was done as previously described [25]. Briefly, Co01 cells were treated with 2 μM Manidipine-2HCl or DMSO for 1 h. Then, cells were harvested and washed with PBS. 10 aliquots, each of 0.75 × 10^6^ cells in 100 μL PBS, were distributed in a 96-well PCR plate and pelleted by centrifugation (300 × *g* for 3 min) before the removal of most of the supernatant (80 μL). Each aliquot was subsequently heated for 3 min to incremental temperatures (37 °C, 40.4 °C, 44 °C, 46.9 °C, 49.8 °C, 52.9 °C, 55.5 °C, 58.6 °C, 62 °C, 66.3 °C) in a PCR machine (Agilent SureCycler 8800) followed by 3 min at room temperature. 30 μL ice-cold lysis buffer (final concentration 0.8% NP-40, 1.5 mM MgCl_2_, protease inhibitors, phosphatase inhibitors, 0.4 μ/μL benzonase) was used to lyse the cells on a shaker (500 rpm) at 4 °C for 1 h. Then, cell debris was removed by centrifuging the PCR plate at 300 × *g* for 3 min at 4 °C, and the supernatant was filtered at 300 × *g* for 3 min at 4 °C through a 0.45-μm 96-well filter plate (Millipore, MSHVN4550) to remove protein aggregates. 25 μL of the flow-through was mixed with 2× sample buffer (180 mM Tris pH = 6.8, 4% SDS, 20% glycerol, 0.1 g bromophenol blue) and stored at –20 °C until prepared for mass spectrometry analysis. The protein concentration was determined by using the remainder in a BCA (ThermoFisher Scientific). Samples were diluted to 1 μg/μL in 1x sample buffer based on the protein concentrations in the lowest two temperatures (37 °C, 40.4 °C).

#### Cellular thermal shift assay for UGT8 specific inhibitor: UGT8i19

To validate the interaction between the UGT8i19 and its corresponding target UGT8, a simplified version of thermal shift assay was performed. Briefly, Co01 was treated with 2 μM UGT8i19 or DMSO for 1 h. Then, cells were harvested and heated to incremental temperatures (37 °C, 40.5 °C, 43.3 °C, 46.4 °C, 49.8 °C, 53.2 °C, 56.6 °C, 59.7 °C, 62.5 °C, 66.3 °C) in a PCR machine followed by 3 min at room temperature. Soluble fractions of each condition were obtained as described previously. 10 μL of each flow-through was immunoblotted with UGT8 Polyclonal antibody (1:1000, Proteintech 17982-1-AP). The grayscale values of bands were quantified by ImageJ. Relative non-denatured fraction (all normalized to grayscale value of 37 °C) was calculated and melting curve was plotted.

#### Proteomics: MS sample preparation and measurement

Proteins were digested as previously described [26]. In brief, 10 μg of protein (calculated from the protein concentrations in the lowest two temperatures) was mixed with a bead suspension (10 μg of beads (Thermo Fischer Scientific—Sera-Mag Speed Beads, 4515-2105-050250, 6515-2105-050250) in 10 μl 15% formic acid and 30 μl ethanol). Mixtures were pre-incubated on a shaker (500 rpm) for 15 min at RT before washed four times with 70% ethanol. Overnight protein digestion was performed in 40 μl digest solution (5 mM chloroacetamide, 1.25 mM TCEP, 200 ng trypsin, and 200 ng LysC in 100 mM HEPES pH 8). Peptides were reconstituted in 10 μl of water after eluting from the beads and vacuum-drying. Peptides were further labeled at room temperature for 1 h with 18 μg of TMT10plex (Thermo Fisher Scientific) dissolved in 4 μl of acetonitrile. The reaction was quenched with 4 μl of 5% hydroxylamine, and samples were combined by temperature. StageTips [27] were used to acidify and desalt the samples that were then eluted with 2 × 30 μl of buffer B (80% acetonitrile, 0.01% TFA). Sample fractionation was performed to generate 3 fractions (Fraction No. 3, 7 and 8) by using the Pierce™ High pH Reversed-Phase Peptide Fractionation Kit (ThermoFisher Scientific). Peptides were applied to reverse-phase chromatography using a nanoLC-Easy1000 coupled online to a Thermo Orbitrap Q-Exactive HF-X. Peptides were eluted and subjected to tandem mass spectrometry by using a 120 min gradient of buffer B. The mass spectrometer was operated in Top20 mode and dynamic exclusion was applied for 30 s.

#### Data analysis

MS data analysis was performed by Proteome Discoverer (ThermoFisher Scientific, version 2.2). Data were searched against the human UniProt database by parameters: trypsin, missed cleavages 3, peptide tolerance 10ppm, 0.02 Da for MS/MS tolerance. Fixed modifications were carbamidomethyl on cysteines and TMT10plex on lysine. Variable modifications included acetylation on protein N terminus, oxidation of methionine and TMT10plex on peptide N-termini.

#### Abundance and stability score calculation

The Proteome Discoverer output files were loaded into R and merged into a single dataset, which was filtered for duplicates and proteins with less than 2 unique peptides. Data were analyzed using the TPP analysis package (version 3.22.1) [28]. with the TPP-TR method using both the *analyzeTPPTR* function with standard settings, as well as executing the individual workflow steps. The resulting output tables were filtered using the following criteria: (1) a protein must be identified in all 6 samples; (2) the melt point differences between conditions for a protein should all have the same sign (all positive or all negative); (3) for each protein, the smallest melt point difference between Manidipine-2HCl and DMSO samples should be larger than the largest melt point difference between DMSO samples; (4) for each protein, the melt points for the three replicates in each condition (Manidipine-2HCl & DMSO) should be within 1 °C of each other. After filtering, the output tables were compared to find common hits.

### Statistics

The expected drug combination responses were calculated based on BLISS reference model using SynergyFinder [29]. Deviations between observed and expected responses with positive and negative values denote synergy and antagonism respectively. A Bliss score greater than 10 is considered indicative of synergy.

All statistical details of experiments can be found in the Figure legends. All data in this study are represented as mean ± standard deviation, unless otherwise noted. A two-tailed unpaired Mann–Whitney test was performed to analyze the difference between two independent groups. In case other statistical tests were applied, this was noted in the figure legends. ∗*p* ≤ 0.05, ∗∗*p* ≤ 0.01, ∗∗∗*p* ≤ 0.001, ∗∗∗∗*p* ≤ 0.0001. Statistical significance was considered at *p* ≤ 0.05.

### List of antibodies

**Table:** 

Antibody name	Vendor	Cat#
Anti-galactocerebroside antibody, clone mGalC	Merck	MAB342
APC-conjugated Goat Anti-Mouse Ig	BD	550826
Anti-BAX	Cell signaling	2774S
Anti-BAK	Cell signaling	3814S
Goat Anti-Rabbit IgG(H+L), Mouse/Human ads-HRP	Southern Biotech	4050-05
Goat Anti-Mouse IgG(H+L), Human ads-HRP	Southern Biotech	1031-05
Anti-GAPDH (6C5)	Merck	MAB374
Anti human cytochrome c	BD	65981A
Mouse anti-BAX Clone 6A7	BD	556467
Anti human UGT8	Proteintech	17982-1-AP

## Results

### Manidipine-2HCl sensitizes colorectal cancer cells to BCL-XL inhibition

**Fig. 1:**
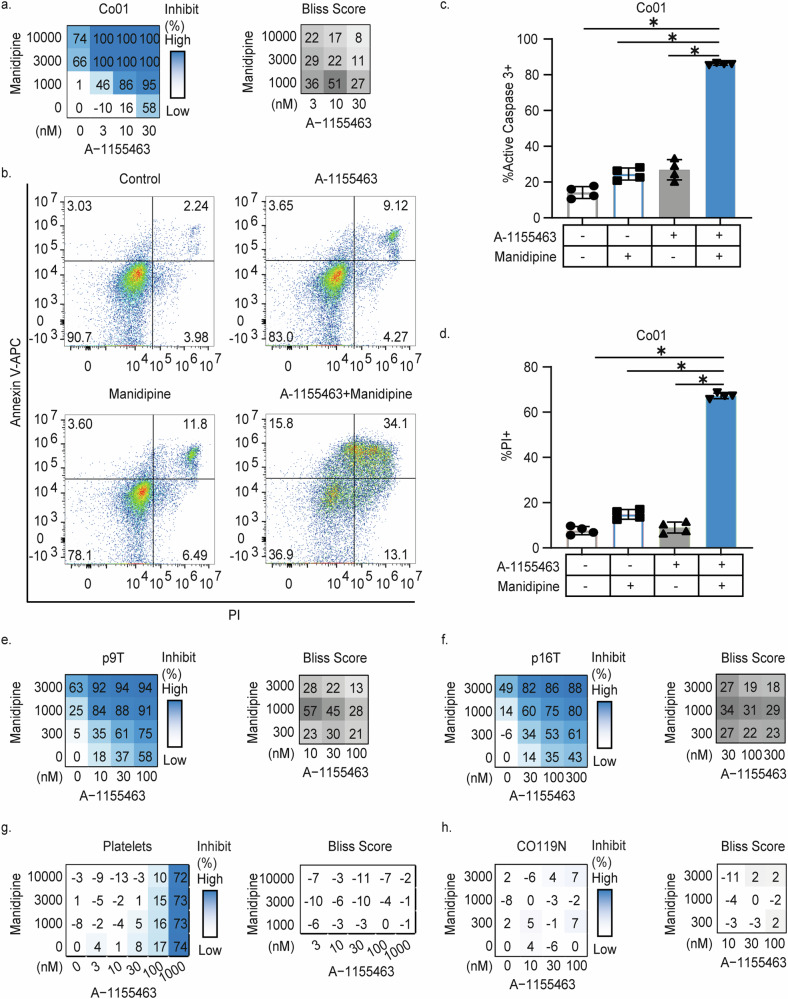
Manidipine-2HCl sensitizes colorectal cancer cells to BCL-XL inhibition. **a** Matrix titration and corresponding Bliss synergy scores of the Manidipine-2HCl and A-1155463 in Co01. Cell viability was measured by CellTiter Blue (*n* = 4). Relative inhibition on viability was plotted and Bliss synergy scores were calculated; **b** Representative plots of Annexin V and PI FACS upon treatment with or without 2 µM Manidipine-2HCl and/or 5nM A-1155463 after 48 h, determined by flow cytometry. *n* = 4; Percentage of Co01 cells showing caspase-3 activity (**c**) or PI positivity (**d**) upon treatment with or without 2 µM Manidipine-2HCl and/or 5nM A-1155463 after 48 h, determined by flow cytometry. *n* = 4; Matrix titration and corresponding Bliss synergy scores of Manidipine-2HCl and A-1155463 treatment of patient-derived tumor organoids p9T(**e**) and p16T (**f**). Cell viability was measured by CellTiter Blue (*n* = 4). Relative inhibition of viability was plotted and Bliss synergy scores were calculated; **g** Matrix titration and corresponding Bliss synergy scores of Manidipine-2HCl and A-1155463 treatment in healthy human platelets.1 μM A-1155463 was used as positive control for platelet killing. Viability was determined by CellTiter Blue (n = 3). Relative inhibition of viability was plotted and the Bliss synergy scores were calculated. **h** Matrix titration and corresponding Bliss synergy scores of Manidipine-2HCl and A-1155463 treatment in human normal colon organoids. Viability was determined by CellTiter Glow (*n* = 3). Relative inhibition on viability was plotted and Bliss synergy scores were calculated. Significance was calculated with Mann–Whitney test; ns: not significant. ∗*p* ≤ 0.05.

To find a safe and CRC-selective combination therapy, we utilized low doses of the BCL-XL specific inhibitor A-1155463 in combination with Manidipine-2HCl, which we have previously shown to provide effective synergy in a drug screen. Manidipine-2HCl, which is developed and clinically approved as calcium channel blocker and anti-hypertensive, displayed the highest synergy [30]. To validate the effectiveness of the combination treatment in a broader set of CRC lines and over a broader concentration range, both drugs were titrated alone or in combination on 3 independent primary patient-derived CRC lines (Co01, Co108, Co147). In all cases very effective synergy was detected (Fig.1a, Supplementary Fig. 1c, d). Importantly, the drop in cell viability as measured by the CellTiter Blue assay was the result of cell death achieved by the combination treatment as indicated by massive induction of caspase-3 activity, exposure of phosphatidylserine (PS) as well as propidium iodide (PI) influx into the cells (Fig.1b–d and Supplementary Fig. 1e, f). Importantly, synergy was evident over a broad range of A-1155463 concentrations and specifically observed with Manidipine-2HCl concentrations above 1 μM (Supplementary Fig. 1a, b) Next to the synergistic toxicity on primary spheroid lines, the synergy was studied in two patient-derived tumor organoids (Fig. 1e, f) and in a panel of 11 CRC cell lines (Supplementary Fig. 1g–q, Supplementary Table 1: Inhibition and Bliss score of all CRC cell lines). Although the concentration at which synergy was detected was higher in the CRC cell lines, a result of the use of fetal calf serum in these cultures (Supplementary Fig. 1r, Supplementary Table 1: HCT-116 Serum complete vs free), in all cases the combination resulted in a strong induction of cell death. In addition to the BCL-XL inhibitor, Manidipine-2HCl was also capable of sensitizing cells to BCL-2 and MCL1 inhibitors, albeit with reduced efficacy as these compounds showed less impact on CRC by themselves requiring much higher concentrations (Supplementary Fig. 1s, t). Notably, in contrast to all CRC cell lines and primary cultures tested, both platelets (Fig. 1g) and normal colon epithelial organoid cultures (Fig. 1h) resisted the combination of Manidipine-2HCl and A-1155463. Only at very high A-1155463 concentrations toxicity on platelets was observed, which confirmed the BCL-XL dependency of these cells but also provided evidence that the combination treatment is tumor selective.

### Manidipine-2HCl sensitized colorectal cancer in a BAX-dependent, but Cav1.1 independent manner

**Fig. 2:**
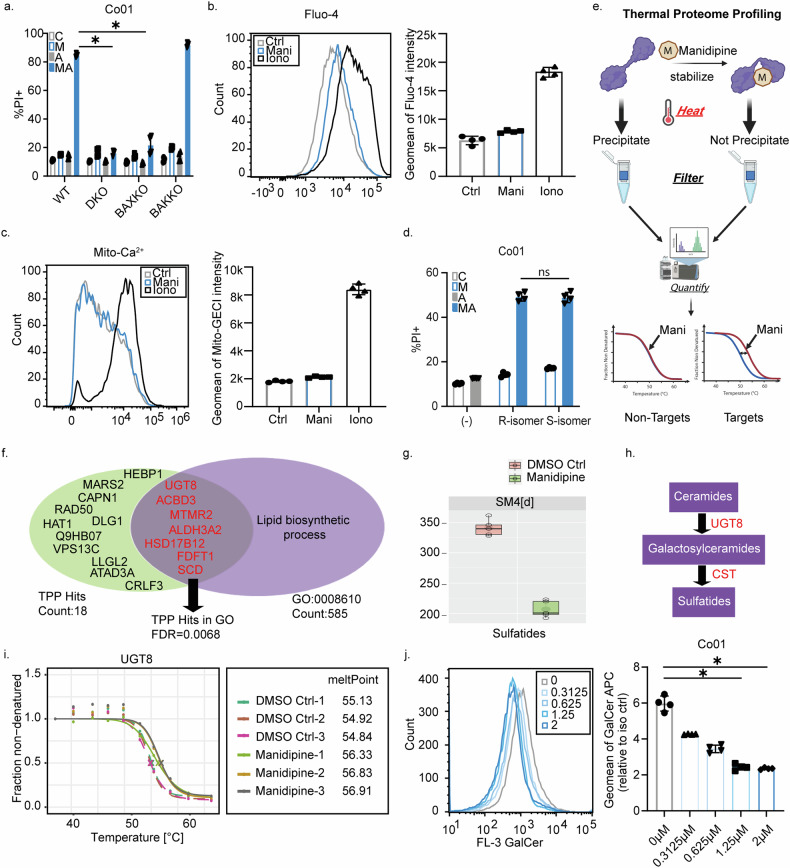
Manidipine-2HCl sensitizes colorectal cancer in a BAX-dependent, but Cav1.1 independent manner and targets UGT8. **a** Cell death induced in BAX (BAX-KO), BAK (BAK-KO) single or double (DKO) knock-out Co01 cells treated with or without 2μM Manidipine-2HCl and/or 5nM A-1155463. C: Control; M: Manidipine-2HCl 2 µM; A: A-1155463 5 nM; MA: Manidipine-2HCl 2 µM + A-1155463 5 nM. Percentage of PI positive Co01 were determined by flow cytometry and plotted; *n* = 4; **b** Intracellular Ca^2+^ levels in Co01 were measured by 1 μM Fluo-4. Cells were treated with 2 μM Manidipine-2HCl for 24 h or 10 μM Ionomycin for 5 min before measuring by flow cytometry. Geometric means of the fluorescence of fluo-4 was plotted; *n* = 4; **c** Mitochondrial Ca^2+^ level in Co01 was measured in cells that contained a transduced mitochondrial targeting Ca^2+^ sensor. Cells were treated with 2 μM Manidipine-2HCl for 24 h or 10 μM Ionomycin for 5 min before measuring by flow cytometry. Geometric means of the fluorescence of the mito-Ca^2+^ sensor was plotted; *n* = 4; **d** Cell death induced in Co01 treated with or without 2 μM of the two enantiomers of Manidipine (R-; S-) and/or 5nM A-1155463. C: Control; M: Manidipine-2HCl 2 µM; A: A-1155463 5 nM; MA: Manidipine-2HCl 2 µM + A-1155463 5 nM. Percentage of PI positive Co01 were determined by flow cytometry and plotted; *n* = 4; **e** Schematic illustration of Thermal Proteome Profiling (TPP). **f** Network cluster analysis of the hits of TPP was performed by STRING v.12. The false discovery rate(FDR) of the association of the cluster with lipid biosynthetic process (GO:008610) was calculated. **g** Sulfatide quantification by lipidomic profiling on Co01 treated with or without 2 μM Manidipine-2HCl for 24 h. The relative mass spectometry readout was normalized to the quantity of total input protein; *n* = 5; **h** Schematic diagraph of sulfatide biosynthesis; **i** Thermostability curve of UGT8 in Co01 treated with or without 2 μM Manidipine-2HCl. Melt points of each treatment were calculated as described in methods; *n* = 3; **j** Flow cytometry of galactosyl-ceramide (GalCer) in Co01 treated without (0 μM) or with 0.3125, 0.625, 1.25 and 2 μM Manidipine-2HCl for 24 h. Geometric means of the relative fluorescence of GalCer probe to mouse IgG isotype was plotted; *n* = 4. Significance was calculated with Mann–Whitney test; ns: not significant, ∗*p* ≤ 0.05.

BAX and BAK are crucial mediators of mitochondrial-dependent apoptosis and therefore expected to coordinate A-1155463-dependent apoptosis. To determine whether these canonical executors of apoptosis are also crucial for the synergism, BAX and BAK single (KO) and double knockouts (DKO) CRC lines were generated and verified (Supplementary Fig. 2a). Intriguingly, whereas BAK-KO cells were equally sensitive to the combination treatment, both the BAX-KO and the BAX/BAK-DKO lines were completely resistant to Manidipine-2HCl and A-1155463 treatment (Fig. 2a), suggesting that BAX is the key regulator of cell death induction in this combination treatment. Interestingly, the pan-caspase inhibitor, Q-VD-Oph, only partially rescued cell death induced by this combination (Supplementary Fig. 2b), suggesting that while caspase activation was involved, inhibiting caspase alone is not sufficient to prevent cell death, as alternative cell death pathways may be activated.

Manidipine-2HCl is reported to target L type Ca^2+^ channel subunit α1 (Cav1.1) and prevent calcium fluxes over the plasma membrane. However, mRNA expression of Cav1.1 revealed a variable and often very low expression in the CRC lines that showed sensitivity to the combination treatment (Supplementary Fig. 2c). Moreover, the cytosolic Ca^2+^ levels, as assessed by the Ca^2+^ fluorescent probe Fluo-4 over a time course, rather showed a slight increase than decrease during a 24 h Manidipine-2HCl treatment (Fig. 2b, Supplementary Fig. 2d). Importantly, mitochondrial Ca^2+^ levels were not affected by Manidipine-2HCl (Fig. 2c, Supplementary Fig. 2e), while classical regulators like EGTA and ionomycin gave the expected changes, suggesting that Manidipine-2HCl may not sensitize CRC lines through inhibition of L type Ca^2+^ channels. In agreement, the two enantiomers of Manidipine-2HCl both showed similar activity in sensitizing CRC cells to A-1155463 (Fig. 2d), while the S-Manidipine-2HCl form has reported 30–80 times higher potency in inhibiting Cav1.1 [31]. Similarly, four other dihydropyridines, all of which are effective Cav1.1 inhibitors, neither synergized with 5nM A-1155463 (Supplementary Fig. 2f–i) nor reduced Ca2+ levels (Supplementary Fig. 2j, k). Combined these observations indicated that Manidipine-2HCl displayed off-target activity in its sensitization of CRC cells to the BCL-XL inhibitor.

### Inhibition of UGT8-induced sulfatide formation is central to apoptosis induction

To identify the target of Manidipine-2HCl, we made use of thermal proteome profiling (TPP). As illustrated in Fig. 2e and Supplementary Fig. 2l, binding of Manidipine-2HCl to a target is expected to alter the biochemical properties of the target resulting in a potential melting temperature shift. To maximize the potential to identify direct binders of Manidipine-2HCl, we performed the TPP analysis 1 h after the addition of Manidipine-2HCl to the CRC cells and found that 18 proteins significantly shifted their melting curves (Fig. 2f, Supplementary Tables 2, 3 and Supplementary Data 1). Strikingly, 7 out of these 18 proteins were associated with lipid biosynthesis (Fig. 2f). Therefore, to determine whether Manidipine-2HCl impacted the lipid composition of cells, lipidomics was performed. Interestingly, the majority of lipids did not reveal significant changes upon treatment (Supplementary Table 4). However, a significant and strong reduction was observed in sulfated sphingolipids, specifically the sulfatides (Fig. 2g), which suggested that Manidipine-2HCl affected sulfatide biosynthesis (Fig. 2h). This is in line with the TPP analysis in which UGT8 displayed the strongest shift in its melting curve (Fig. 2i and Supplementary Tables 2 and 3). Unfortunately, the direct product of the reaction catalyzed by UTG8, galactosyl-ceramide (GalCer) cannot be directly monitored by mass spectrometry analysis as it is identical to its diastereoisomer glucosyl-ceramide, which is part of another biosynthethic pathway. To circumvent this, an antibody to GalCer was used and staining of Manidipine-2HCl-treated cells confirmed the significant dosage-dependent reduction in GalCer (Fig. 2j), which subsequently leads to the reduction of the downstream metabolite sulfatide. To confirm the role of the sulfatide biosynthesis in sensitization of CRC cells to the BCL-XL inhibitor A-1155463, a selective UGT8 inhibitor, UGT8i19, was tested and its suspected binding to UGT8 was confirmed using TPP (Supplementary Fig. 2m). Compared to Manidipine-2HCl, UGT8i19 was even more potent in decreasing sulfatides and GalCer levels in CRC cells (Supplementary Fig. 2n, o). Intriguingly, UGT8i19 also sensitized CRC cells to low dose of the BCL-XL inhibitor (Supplementary Fig. 2p), further supporting the hypothesis that the lipid metabolism pathway controlled by UGT8 is crucial in defining the impact of BH3 mimetics targeting BCL-XL.

To further validate the specificity of the effects observed in Manidipine-2HCl and UGT8i19-treated cells, we generated a UGT8 knockout (UGT8-KO) Co01 cell line using the CRISPR-Cas9 system. This knockout was confirmed by TIDE analysis(Supplementary Fig. 2q). As expected, the knockout of UGT8 led to a significant reduction in GalCer levels in the UGT8-KO line, while Manidipine-2HCl and UGT8i19 failed to further decrease GalCer levels due to the absence of the biochemical activity of their target UGT8 (Supplementary Fig. 2r). The genetic knockout of UGT8 or knockout of cerebroside sulfotransferase (CST-KO), the downstream enzyme essential for sulfatides production (Fig. 2h), did not independently increase sensitivity to A-1155463 (Supplementary Fig. 2s–u), likely due to metabolic reprogramming and lipid adaptation during the generation of the UGT8-KO and CST-KO line. Nevertheless, a significant reduction of the sensitization effects of Manidipine-2HCl and UGT8i19 was observed in the UGT8-KO line (Supplementary Fig. 2v). This finding suggested that sensitization by Manidipine-2HCl and UGT8i19 required UGT8. Collectively, these results highlighted the role of UGT8-driven sulfatide production in the regulation of mitochondrial-dependent apoptosis in CRC.

### Inhibition of sulfatide synthesis impacted on mitochondrial morphology

**Fig. 3:**
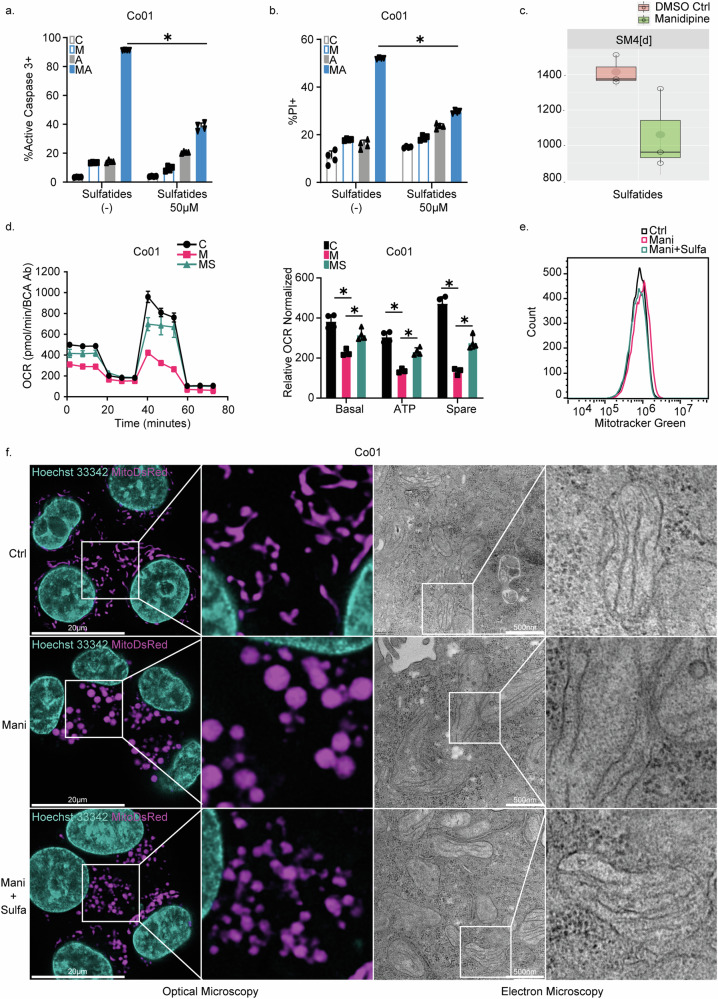
Inhibition of sulfatide synthesis impacted mitochondrial morphology. **a** Caspase-3 activity in Co01 cells after treatment for 48 h with 2 μM Manidipine-2HCl and/or 5 nM A-1155463 in the presence or absence of 50 μM sulfatides as determined by flow cytometry. C: Control; M: Manidipine-2HCl 2 µM; A: A-1155463 5 nM; MA: Manidipine-2HCl 2 µM + A-1155463 5 nM; *n* = 4; **b** Percentage of PI positive Co01 induced by 2 μM Manidipine-2HCl and/or 5nM A-1155463 in the presence or absence of 50μM sulfatides after 48 h, determined by flow cytometry. C: Control; M: Manidipine-2HCl 2 µM; A: A-1155463 5 nM; MA: Manidipine-2HCl 2 µM + A-1155463 5 nM; *n* = 4; **c** Co01 treated with or with 2 μM Manidipine-2HCl for 24 h were used to determine the level of sulfatides in isolated heavy membrane fraction that primarily consists of mitochondria by lipidomics; *n* = 3. **d** Oxygen consumption rate (OCR) of Co01 treated with 2 μM Manidipine-2HCl with or without 50μM sulfatides were measured by seahorse mito stress assay. The basal, ATP production related and spare respiratory capacity were plotted as shown by the right lane. C: Control; M: Manidipine-2HCl 2 µM; MS: Manidipine-2HCl 2 µM+sulfatides 50 µM; *n* = 4. **e** Mitochondrial mass in Co01 was measured by 50 nM Mitotracker Green. Cells were treated with 2 μM Manidipine-2HCl with or without 50 μM sulfatides for 24 h before measuring by flow cytometry; *n* = 4. **f** The mitochondrial morphology was determined with mitochondria targeting DsRed (MitoDsRed) after treatment with and without 2 μM Manidipine-2HCl and/or 50 μM sulfatides (left two panels). Mitochondria were imaged with confocal microscope at 63× magnification. Right two panels depict the mitochondrial morphology after fixation and imaging by electron microscopy. Size bars are indicated and significance was calculated with Mann–Whitney test; ns: not significant, ∗*p* ≤ 0.05.

UGT8 is often upregulated in cancer and its expression is associated with poor prognosis in breast cancer patients [32]. As inhibition of this enzyme by UGT8i19 or Manidipine-2HCl had a major impact on the cellular levels of sulfatide, exogenously added sulfatides should be able to revert the synergistic induction of cell death. To verify the restoration of sulfatide levels in cells treated with exogenous sulfatides, lipidomic analysis was performed on cells treated with 50 µM sulfatides. The results confirmed a clear increase in sulfatide levels with especially long fatty acid side chains, but not those with shorter side chains (less than C36, especially C34), reflecting the composition of the exogenously added sulfatides, which are derived from porcine brain (Supplementary Fig. 3a, b). In agreement, addition of 50 μM of sulfatides to the culture medium could significantly rescue multiple CRC lines from Manidipine-2HCl/A-1155463-induced apoptotic cell death (Fig. 3a, b and Supplementary Fig. 3c, d). As expected, exogenously added sulfatides also rescued CRC cells from the combination treatment of UGT8i19 and A-1155463 (Supplementary Fig. 3e). Intriguingly, sulfatides were also detected by mass spectrometry lipid analysis in the heavy membrane (HM) fraction of CRC cells, which primarily consists of mitochondria with some contamination from endoplasmic reticulum (ER) markers (Supplementary Fig 3f) and displayed a significant reduction in quantity upon Manidipine-2HCl treatment alone (Fig. 3c), suggesting that mitochondrial homeostasis could depend on sulfatides. In agreement, Manidipine-2HCl induced massive changes in the mitochondrial morphology of CRC cells. Firstly, mitochondrial respiration was strongly impaired by treatment with Manidipine-2HCl in multiple CRC cell lines and this was partly restored by sulfatides (Fig. 3d and Supplementary Fig. 3g), whereas the mitochondrial respiration was not impacted by BCL-XL inhibitor, A-1155463 (Supplementary Fig. 3h). This reduction induced by Manidipine-2HCl was not due to a change in the mitochondrial content as quantification of mitochondrial mass with Mitotracker Green did not reveal differences (Fig. 3e). This finding was further corroborated by the unchanged mitochondrial DNA copy number (Supplementary Fig. 3i) and total mitochondrial area, as assessed from mito-DsRed images (Supplementary Fig. 3j). Additionally, the reduction in respiration was not attributable to a collapse of mitochondrial membrane potential (Supplementary Fig. 3k). Subsequent visualization and stratification of the mitochondria with mito-DsRed indicated that Manidipine-2HCl treated cells contained heavily swollen mitochondria (Fig. 3f, Supplementary Fig. 3l, m). This was confirmed by electron microscopy, which pointed to an inflated mitochondrial matrix (Fig. 3f, Supplementary Fig. 3n, o). Interestingly, the effect of Manidipine-2HCl on mitochondria is dose-dependent (Supplementary Fig. 3p) and perfectly aligned with sensitization (Supplementary Fig. 1a). Only the doses that induced mitochondrial morphology change resulted in sensitization to the BCL-XL inhibitor A-1155463, further indicating that this effect on mitochondria is associated with the sensitization process. The role of sulfatide, and specifically the reduction in the levels of sulfatide, in these morphological changes was again confirmed by the addition of exogenous sulfatides (Fig. 3f, Supplementary Fig. 3l–o), suggesting that they play a crucial role in mitochondrial function. Consistently, Manidipine-2HCl failed to induce mitochondrial swelling in cells lacking UGT8, further confirming UGT8 as the target of Manidipine-2HCl in regulating mitochondrial morphology (Supplementary Fig. 3q–s).

Mitochondrial damage typically induces the production of mitochondrial reactive oxygen species (mitoROS). While Manidipine-2HCl did elevate mitoROS levels (Supplementary Fig. 3t), the use of a mitoROS scavengers did not prevent sensitization to cell death (Supplementary Fig. 3u), suggesting that mitoROS induction was not responsible for the observed sensitization. Importantly, the severe impact on mitochondrial morphology led to cell death over longer periods of Manidipine-2HCl exposure (5 days) (Supplementary Fig. 3v, w). Notably, the pan-caspase inhibitor Q-VD-Oph failed to protect the cells (Supplementary Fig. 3v), whereas sulfatides successfully maintained cell survival during long-term Manidipine-2HCl treatment (Supplementary Fig. 3w). This finding further supports the conclusion that Manidipine-2HCl impairs the sulfatide pathway, affecting both mitochondrial function and cell viability.

### Mitochondrial sulfatide regulated BAX localization on the mitochondria

**Fig. 4:**
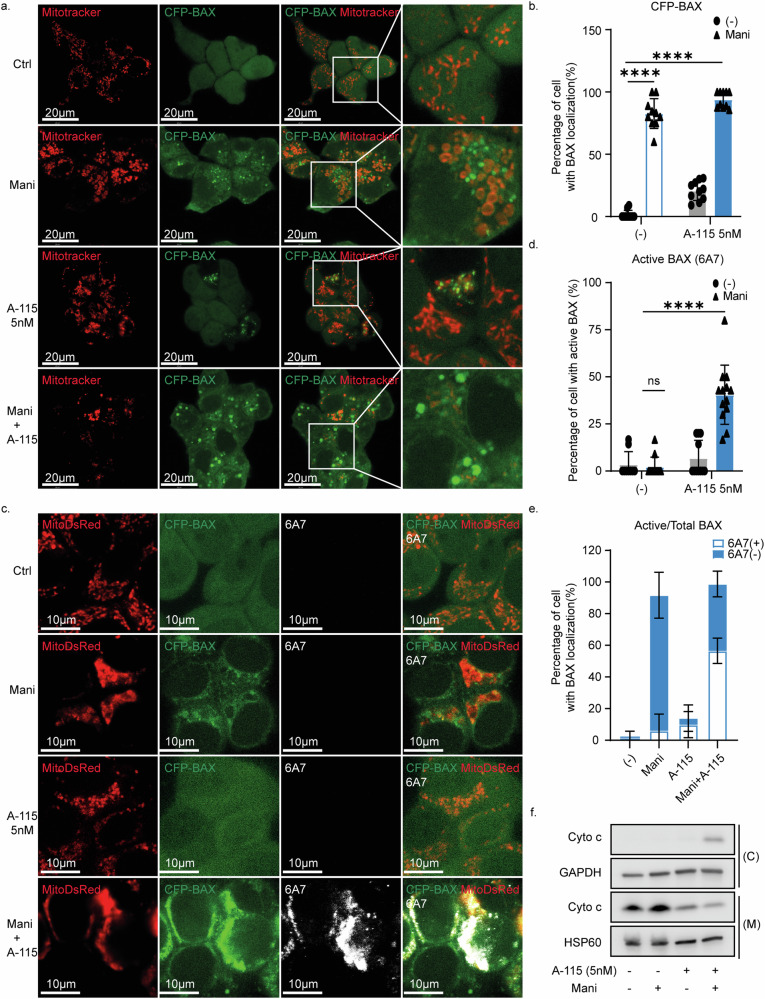
Inhibition of sulfatide regulated BAX localization on the mitochondria. **a** CFP-BAX localization was imaged with confocal microscope at 63× magnification. Co01-BAX-KO-CFP-BAX were treated with 2 μM Manidipine-2HCl for 24 h followed by 5 nM A-1155463 stimulation for 3 h. 50 nM Mitotracker Deep Red was used to stain mitochondria; **b** CFP-BAX localization was analyzed using multiple fields of imaging. Percentage of cells with CFP-BAX localized to the mitochondria was calculated and plotted; *n* = 10; **c** Active BAX immunofluorescent staining with mouse anti-BAX monoclonal antibody 6A7 clone. Co01-BAXKO-CFP-BAX-MitoDsRed were treated with or without 2 μM Manidipine-2HCl for 24 h and where indicated followed by 5 nM A-1155463 stimulation for 3 h before fixation. Images were obtained with confocal microscope at 63× magnification; **d** BAX 6A7 staining was analyzed using multiple fields of imaging. Percentage of the cells with 6A7-positive staining was calculated and plotted; *n* = 16; **e** Percentage of cell with active BAX (6A7 positive) in those cells with BAX localization (CFP-BAX localization) to mitochondria. *n* = 6; **f** MOMP induction was examined by detecting cytosolic cytochrome c (Cyto c) in Co01 cells treated with 2 Manidipine and 5nM A-1155463 alone or in combination. Both cytosolic(C) and the rest (containing mitochondrial fraction (M)) of cells were blotted. GAPDH and HSP60 were used as the loading control for cytosolic and mitochondrial fraction respectively. Significance was calculated with Mann–Whitney test; ns: not significant, ∗∗∗∗*p* ≤ 0.0001.

The central role of BAX in the combination treatment, which mediates mitochondrial outer membrane permeabilization (MOMP), combined with the impact on mitochondrial morphology induced by the sulfatide reduction, prompted us to analyze BAX localization dynamics. To this end, CFP-BAX was transduced in Co01-BAX-KO cells, which resulted in relatively high BAX levels (Supplementary Fig. 4a). This expression did not induce apoptosis by itself and only slightly sensitized the cells to BCL-XL inhibition (Supplementary Fig. 4b). However, the sensitization effect was indeed further enhanced when combined with Manidipine-2HCl (Supplementary Fig. 4c). Under normal conditions BAX was dispersed in the cytoplasm and only little BAX was found to be localized to the mitochondria (Fig. 4a). In contrast, 24 h of Manidipine-2HCl treatment alone resulted in a significant shift in BAX localization, with close to a 100% of the cells displaying mitochondria with BAX aggregates (Fig. 4a, b and Supplementary Fig. 4d). A similar shift in BAX localization was induced by UGT8i19 and very high doses of A-1155463 (Supplementary Fig. 4d). Not surprisingly, the extend of BAX localization to the mitochondria could be further enhanced by addition of low dose of A-1155463 to Manidipine-2HCl (Fig. 4a, b). Interestingly, Antimycin A, a Complex III inhibitor and a known inducer of mitochondrial swelling, also promotes BAX localization to mitochondria, albeit in a pattern different from that of Manidipine-2HCl (Supplementary Fig. 4d). It similarly sensitized cells to the BCL-XL inhibitor A-1155463 (Supplementary Fig. 4e), further suggesting a possible connection between mitochondrial swelling and BAX localization. Although BAX localization to the mitochondrial membrane is a prerequisite for MOMP, it is by itself not sufficient to permeabilize the mitochondrial membrane as BAX requires further activation to insert into the lipid bilayer. This insertion is detectable with the 6A7 anti-BAX antibody (Supplementary Fig. 4f) but only upon the addition of high doses of A-1155463 and crucial for the formation of pore structures. Interestingly, although Manidipine-2HCl induced the mitochondrial localization of BAX, it failed to induce the insertion into the membrane as shown by the absence of staining with the 6A7 antibody that recognizes the active/inserted conformation (Fig. 4c–e). In contrast, the addition of low dose A-1155463 quickly changed the BAX state from a mitochondrial residing form to a mitochondrial outer membrane-inserted form (Fig. 4c, d) leading to the rapid induction of MOMP and release of cytochrome c into cytosol (Fig. 4f).

**Fig. 5:**
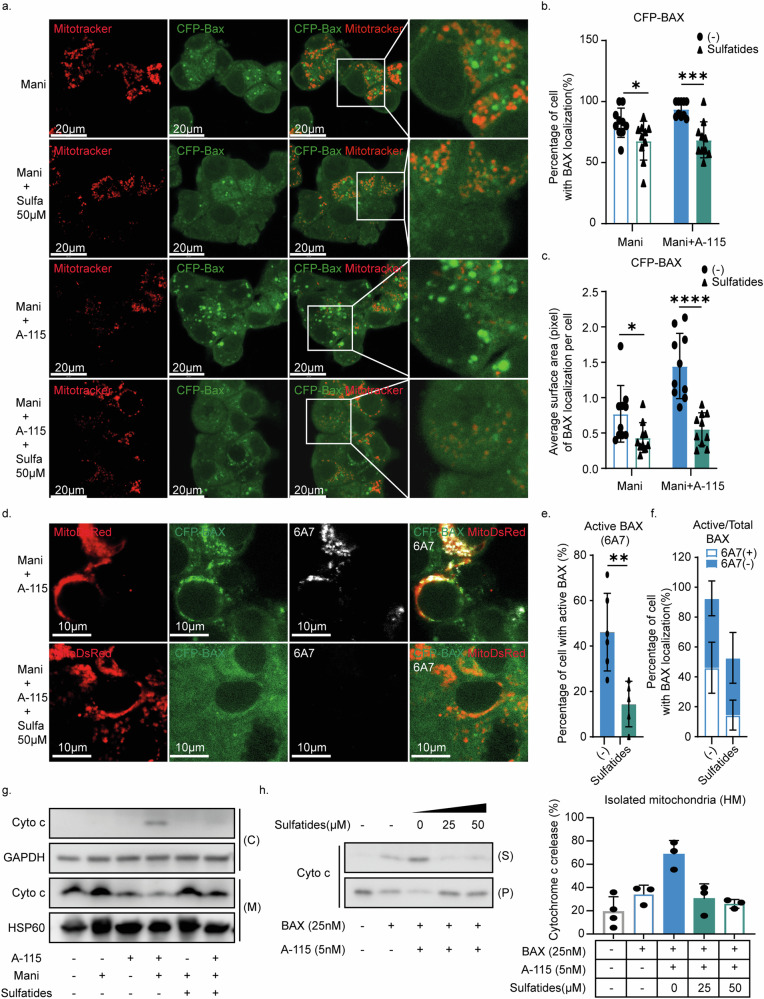
Mitochondrial sulfatide regulated BAX localization and directly prevented MOMP. **a** CFP-BAX localization was imaged with confocal microscope at 63× magnification. Co01-BAX-KO-CFP-BAX were treated with 2 μM Manidipine-2HCl with or without 50 μM sulfatides for 24 h followed by 5nM A-1155463 stimulation for 3 h. 50 nM Mitotracker Deep Red was used to stain mitochondria; *n* = 10; **b** Quantification of the cells positive for CFP-BAX localization to the mitochondria and (**c**) the average surface areas of the fluorescent spots per cell treated with 2 μM Manidipine-2HCl with or without 50 μM sulfatides for 24 h followed by 5 nM A-1155463; *n* = 10; **d** Active BAX immunofluorescent staining with mouse anti-BAX monoclonal antibody 6A7 clone. Co01-BAXKO-CFP-BAX-MitoDsRed were treated with 2 μM Manidipine-2HCl with or without 50 μM sulfatides for 24 h and where indicated followed by 5nM A-1155463 stimulation for 3 h before fixation. Images were obtained with confocal microscope at 63× magnification; **e** BAX 6A7 staining were analyzed using multiple fields of imaging. Percentage of the cells with 6A7-positive staining was calculated and plotted; *n* = 6; **f** Percentage of cell with active BAX(6A7 positive) in those cells with BAX localization (CFP-BAX localization) to mitochondria, *n* = 6; **g** MOMP induction was examined by detecting cytosolic cytochrome c (Cyto c) in Co01 cells treated with 2 Manidipine-2HCl and 5nM A-1155463 alone or in combination in the presence or absence of 50 μM sulfatides. Both cytosolic(C) and the rest (containing mitochondrial fraction (M)) of cells were blotted. GAPDH and HSP60 were used as the loading control for cytosolic and mitochondrial fraction respectively. **h** In vitro cytochrome c release assay was performed on isolated mitochondria within heavy membrane fraction pretreated with or with sulfatides. Both the released fraction (S) and the pellet fraction (P) of mitochondria were blotted. Grayscale of bands was quantified by imageJ and relative release of cytochrome c (supernatant: pellet) was calculated and plotted, *n* = 3. Significance was calculated with Mann–Whitney test; ns: not significant, ∗*p* ≤ 0.05, ∗∗*p* ≤ 0.01, ∗∗∗*p* ≤ 0.001, ∗∗∗∗*p* ≤ 0.0001.

Strikingly, exogenously added sulfatides, not affecting BAX localization by itself (Supplementary Fig. 5a), dramatically reduced the Manidipine-2HCl-induced localization of BAX to the mitochondria both in the amount of cells showing localization and in the extend of the localization (Fig. 5a–c). Consequently, the addition of exogenous sulfatides also reduced BAX activation (Fig. 5d–f) and the subsequent induction of MOMP (Fig. 5g). To determine whether this effect of sulfatides on mitochondria is direct or an indirect result caused by sulfatide loss, isolated mitochondria in heavy membrane fraction were permeabilized with a combination of recombinant BAX and the BCL-XL inhibitor A-1155463, with or without sulfatides. Notably, pre-incorporation of sulfatides significantly reduced OMM permeability and cytochrome c release (Fig. 5h), indicating a direct effect of sulfatides on mitochondria that mitigates BAX-mediated MOMP. All these observations provided further evidence that a change in the mitochondrial lipid constitution regulates mitochondrial function as well as mitochondrial BAX localization.

**Fig. 6:**
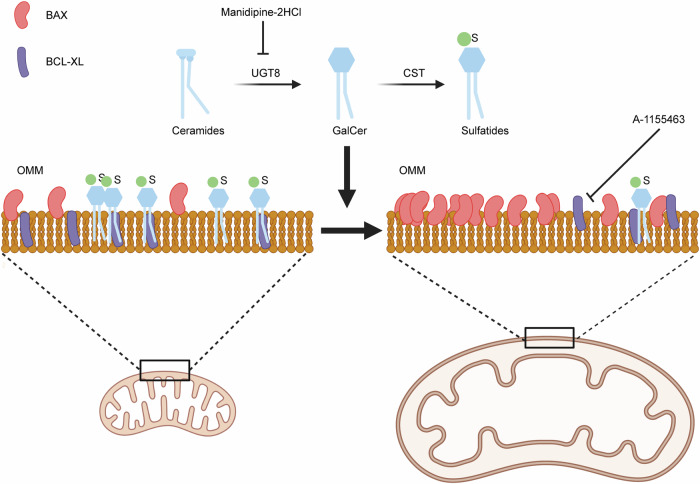
Model of the molecular mechanism of the Manidipine-2HCl induced sensitization in CRC cell. Manidipine-2HCl inhibits the UGT8 mediated synthesis of sulfatides that subsequently leads to the reduction of mitochondria sulfatides. This results in a localization of BAX to mitochondria. Localized BAX is activated by BCL-XL inhibitor and activated BAX permeabilizes the outer mitochondrial membrane.

Manidipine-2HCl or UGT8i19 treated cells are therefore primed to undergo apoptosis due to an enhanced BAX localization to the mitochondria, ready to unleash MOMP. A simply push by A-1155463 is then sufficient to release the brake and induce MOMP (Fig. 6).

## Discussion

Our data reveal that Manidipine-2HCl inhibited UGT8 and impaired sulfatides synthesis. The synergism elicited by Manidipine-2HCl with BH3 mimetic A-1155463 uncovered a complete novel regulatory mechanism that is dependent on sulfatides and organizes mitochondrial morphology as well as regulates BAX localization to the mitochondria. Reduced levels of sulfatides effectively enhance sensitivity to pro-apoptotic insults.

Manidipine-2HCl, a 4th generation dihydropyridine calcium channel antagonist (CCA), was firstly introduced 35 years ago for hypertension treatment with a long-lasting calcium channel blocking effect [33] and widely reported to display metabolic benefit in hypertensive patients [34]. However, in this study, we revealed an off-target effect of Manidipine-2HCl to sensitize CRC cell to apoptosis. In order to pinpoint the direct binder of Manidipine-2HCl responsible for sensitization, we took advantage of TPP [25]. In principle, TPP can detect target proteins if these change thermostability upon Manidipine binding. However, as thermostability of proteins is determined by several factors, such as dynamic post-translational modifications (phosphorylation, ubiquitination) or binding to other proteins, it is difficult to conclude using TPP that Manidipine and UGT8 interact directly. Similarly, it is difficult to determine whether the other hits that were revealed in the TPP analysis represent downstream modified proteins or direct targets of Manidipine-2HCl. Even at such short incubation times (1 h), the thermostability shift of the proteins identified could be the result of an indirect effect for instance mediated by a change in lipid composition of the cells. At this point, it is therefore uncertain whether Manidipiene-2HCl is a direct inhibitor of UGT8. However, the fact that a specific UGT8 inhibitor mirrored the effects observed with Manidipine-2HCl at least confirm that the inhibition of this enzyme is key in the sensitization of CRC cells for the subsequent targeting of BCL-XL. In addition, the reduced Gal-Cer and sulfatide synthesis that was observed with both UGT8-inhibitor and Manidipine-2HCl indicated that the enzymatic activity of UGT8 was affected by Manidipine-2HCl treatment.

UDP-glycosyltransferases (UGTs) are a superfamily of enzymes that catalyze the covalent addition of sugars from nucleotide UDP-sugar donors to a broad range of lipophilic molecules. In mammals, the superfamily comprises four families: UGT1, UGT2, UGT3, and UGT8, which are distinguishably characterized by utilizing distinct UDP-conjugated sugar donors. UGT8, also known as ceramide galactosyltransferase (CGT), uses exclusively UDP-galactose to add galactose to ceramides [35]. Emerging evidence indicates an elevation of this enzyme in different cancers [36, 37]. However, their exact role in the formation of cancer is not fully understood. Under physiological circumstances, UGT8 is crucial for the generation of sulfatides that form an important constituent of the myelin sheath of neurons [38]. More recently, it was reported to be a crucial enzyme in the detoxification of bile acids in the gut, explaining the expression of UGT8 in intestinal epithelium [39]. When looking at the lipid metabolism directly, it is evident that the activity of UGT8 governs the balance between ceramide and its glycosylated metabolites, galactosyl-ceramides (GalCer) and the derived sulfated molecules 3-O-sulfogalactosylceramide, better known as sulfatide. Previously, ceramides were suggested to enhance apoptosis [40–42], while other sphingolipids, i.e., sphingosine-1-phosphate and sphingomyelin, which can be generated directly from ceramide, rather protect from cell death [18, 43, 44]. Although controversial, ceramides were suggested to modulate MOMP at early stages of apoptosis [45, 46] and it was suggested that cancer cells detoxify ceramides by glycosylation into either glucosyl-ceramides (GluCer) [47–49] or galactosyl-ceramides (GalCer) [50]. The latter pathway relates to the observed upregulation of UGT8 [36, 51, 52]. In agreement, sulfatides are found to be elevated in some cancer cells [52]. Here we provide evidence that sulfatides indeed have an anti-apoptotic function, providing an additional rationale for cancer cells to elevate the expression or activity of UGT8, favoring cancer cell survival. Whether UGT8 simply maintains a pro-survival balance in the mitochondria between ceramides and sulfatides remains to be established, but is an interesting option, especially as a clear decrease in the level of sulfatide was also observed in mitochondrial membranes.

The observed sensitization is strongly tumor-selective as no toxicity of the combination treatment was observed in normal colonic organoids even though these cells express UGT8. Whether these cells also generate sulfatide and use sulfatide as protective lipid remains to be determined. However, our previous work showed that normal colonocytes resist BCL-XL targeting due to the expression of BCL-2, which may explain the lack of toxicity of the combination treatment as well [3]. Whether transformation leads to enhanced ceramide synthesis, as reported before, and a subsequent need for a counterbalancing level of sulfatides requires further investigation, but that lipids exert a regulatory role in the formation of MOMP is undisputed [53]. Previously, saturation of the acyl chains of cardiolipin have been reported to regulate cytochrome c release [16] and there is a clear relation between the lipid composition of the OMM and BAX activation [54]. Phosphatidylethanolamine, which is a lipid that leads to negative curvature, inhibited BAX oligomerization without affecting its insertion [54]. Conversely, cardiolipin was essential for BAX insertion and activation [54]. Clifton’s lab further addressed that the cardiolipin-BAX complexes drive pore formation during early stage of apoptosis [55]. Cholesterol, on the other hand, decreased the membrane fluidity that also dampened BAX activation [56]. Finally, the catabolites of ceramide, sphingosines-1 phosphate, and hexadecenal, were shown to cooperate with BAX and BAK to facilitate MOMP [20], suggesting that lipids can directly modify the capacity to permeabilize the mitochondrial membrane. In addition to the actual lipid composition of the membrane, the lipid asymmetry was reported to be associated with BAX-induced permeabilization as well [57]. Intriguingly, we observed that sulfatides play a direct role in protecting isolated mitochondria from permeabilization by BAX and BCL-XL inhibitors, further suggesting that the lipid composition of the mitochondrial membrane is involved in regulating BAX dynamics and apoptosis. However, the precise molecular mechanism by which sulfatides regulate BAX localization remains a topic of debate. Two potential models can be proposed: the direct model and the indirect model. In the direct model, sulfatides residing on the OMM may directly interact with BAX, inhibiting its aggregation on the OMM. In the indirect model sulfatides might target the inner mitochondrial membrane (IMM), leading to respiratory defects and mitochondrial swelling that subsequently trigger stress-induced BAX localization to the mitochondria as reported [58]. Based on our observations of intact isolated mitochondria, we deem the direct inhibition model more likely, hypothesizing that sulfatides incorporated into the OMM directly repel BAX and reduce its localization to the membrane. This is supported by the fact that the protective effects of sulfatides on intact isolated mitochondria are unlikely to involve reversing respiratory defects or mitochondrial swelling, which are less likely to occur in these intact isolated mitochondria. Nevertheless, within the context of intact cells, we cannot entirely exclude the potential role of sulfatides in the IMM, as the observed reductions in respiration and mitochondrial swelling in cells are more likely consequences of impaired inner membrane function, though alterations in the outer membrane could also affect the function of the respiratory chain and contribute to mitochondrial swelling, consistent with the well-documented roles of BAX/BAK in respiration [59]. The complex interdependence between the inner and OMMs complicates the determination of cause and effect. How sulfatides fit into this regulation remains to be established, but it is clear that their synthesis provides a means to regulate mitochondrial homeostasis in CRC. Inactivation of this pathway by Manidipine-2HCl has a major consequence for the shape and function of the mitochondria, allowing these to be covered with BAX that is ready to be activated. Traces of the BCL-XL inhibitor A-1155643 are then sufficient to release the apoptotic machinery. As such, targeting UGT8 in combination with BCL-XL inhibition could provide an interesting avenue to treat CRC.

## Supplementary information

Supplementary Figure 1-5

Supplementary Figure Legends

Supplementary Table 1

Supplementary Table 2

Supplementary Table 3

Supplementary Table 4

Supplementary Table 5

Supplementary Table 6

Supplementary Data 1

Original WBlots

## Data Availability

The authors declare that all data supporting the findings of this study are available within the article and its Supplementary files. Supplementary files contain Inhibition and Bliss score of all CRC cell lines, the cellular Lipidomics original data DMSO vs Manidipine and DMSO vs UGT8i19and the Mitochondrial Lipidomics original data DMSO vs Manidipine. In addition, the complete Thermal proteome profiling data, the hits overview and the individual melting curves are included.
