# Supplementary figures and images for: UGT8 mediated sulfatide synthesis modulates BAX localization and dictates apoptosis sensitivity of colorectal cancer
Source: Cell Death Differ. 2024 Nov 23;32(4):657–71. doi: 10.1038/s41418-024-01418-y (PMC11982410; doi:10.1038/s41418-024-01418-y)

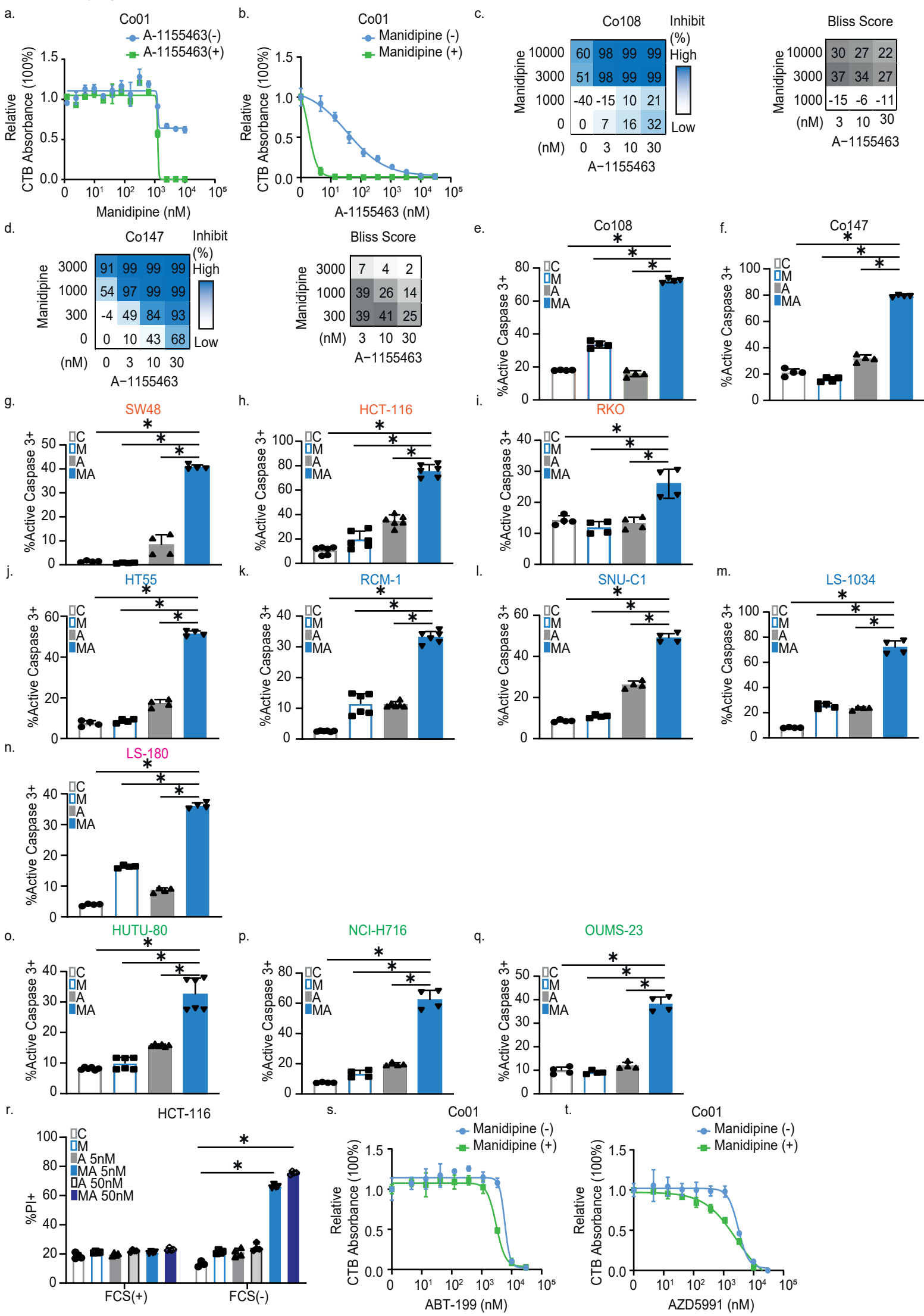

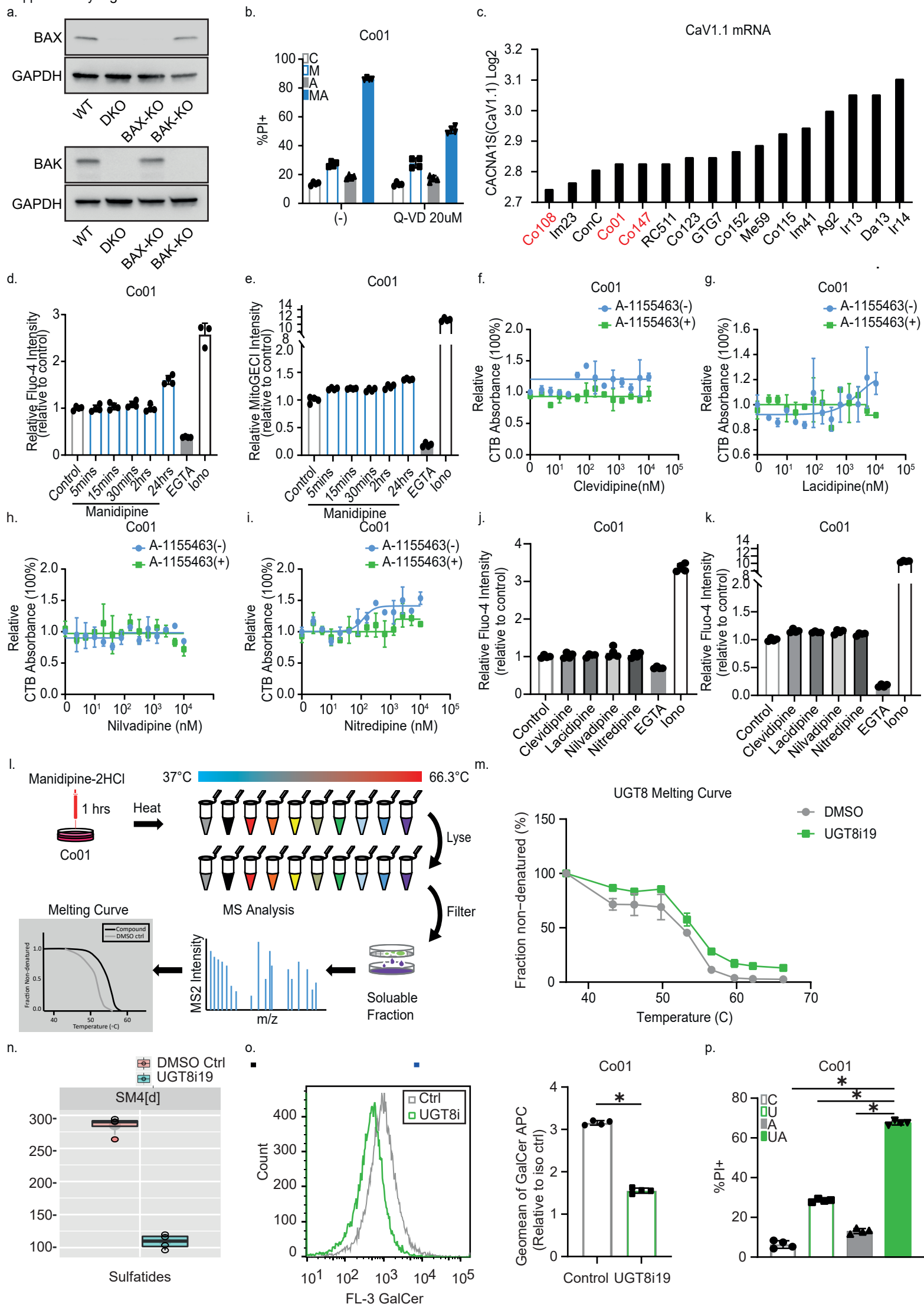

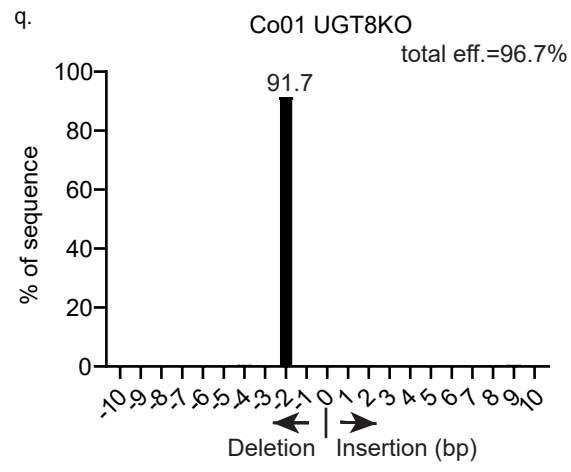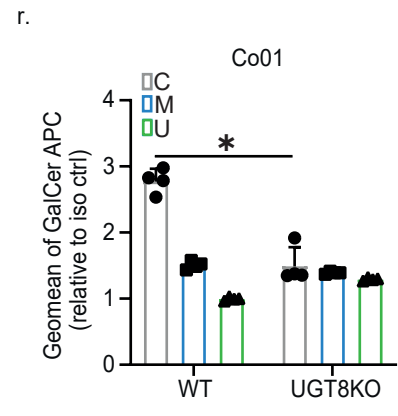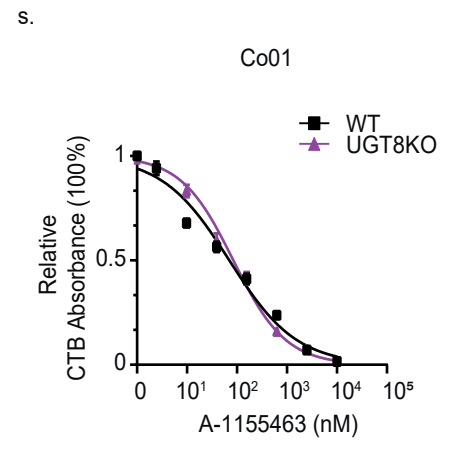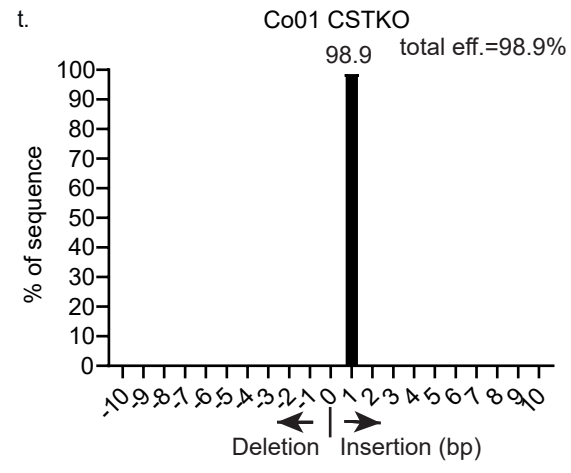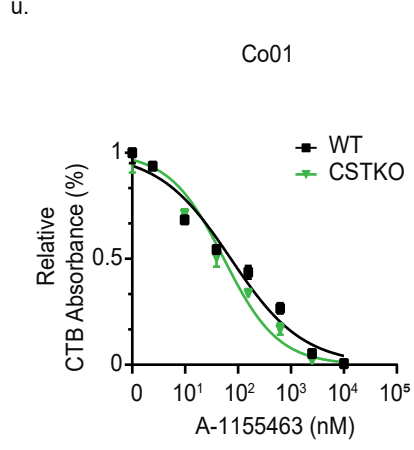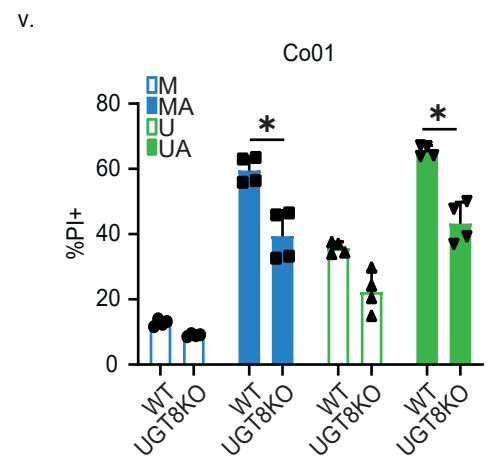

a.

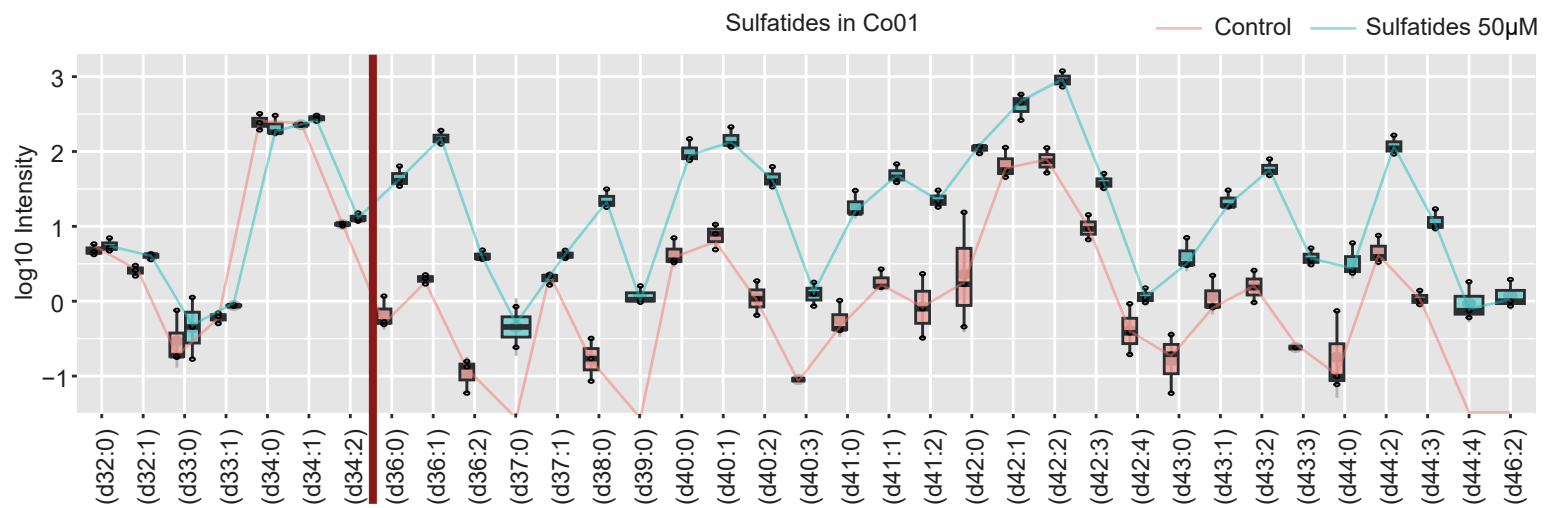

b.

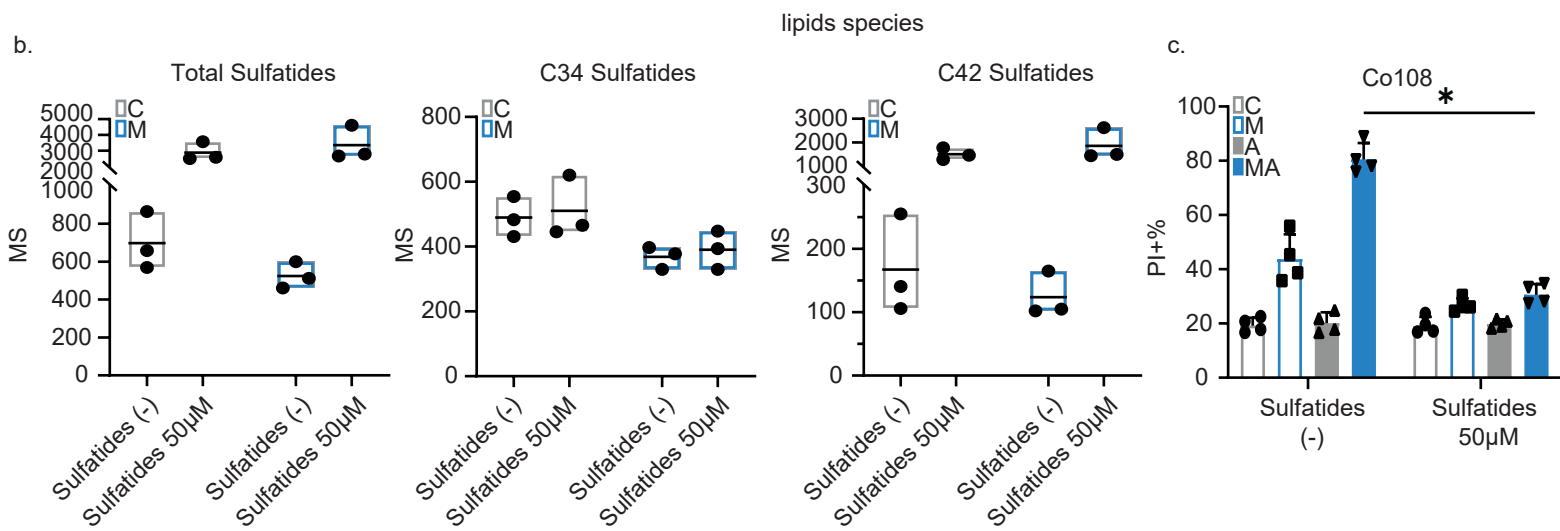

d.

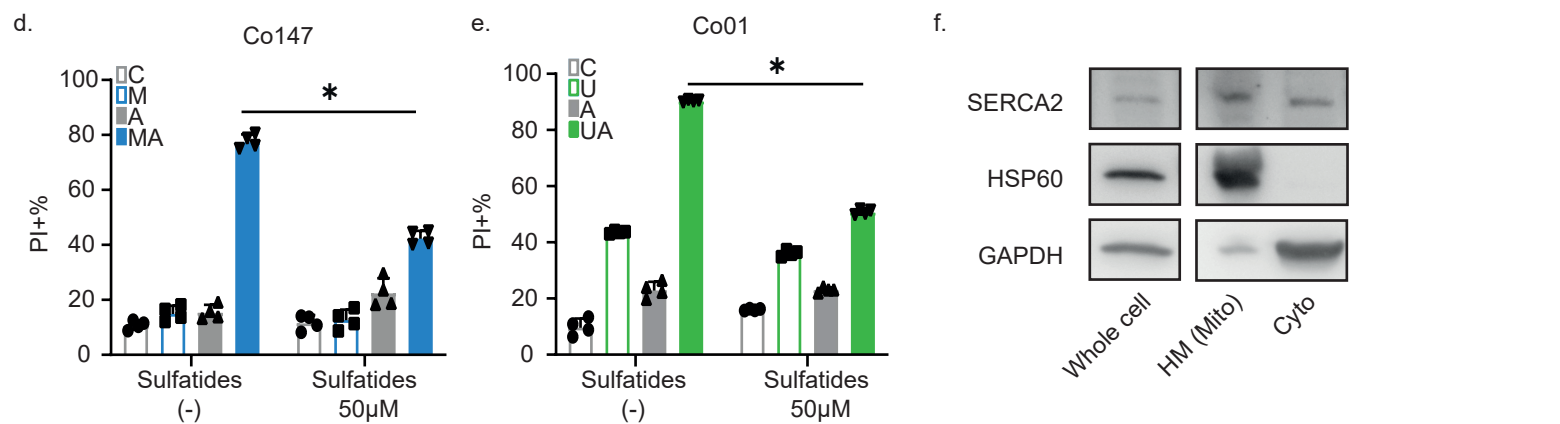

g.

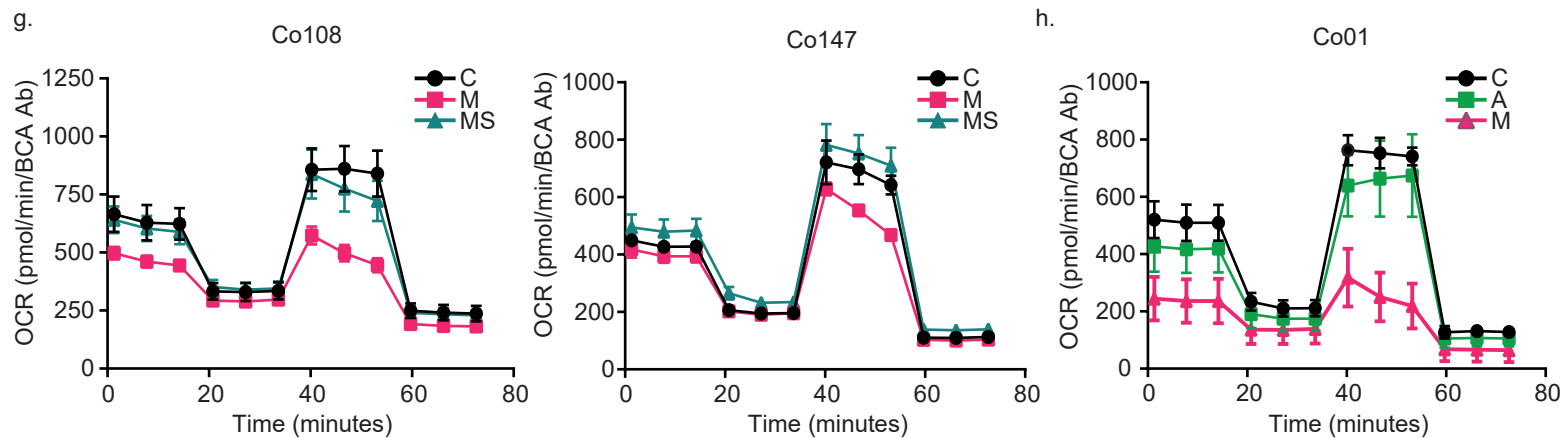

h.

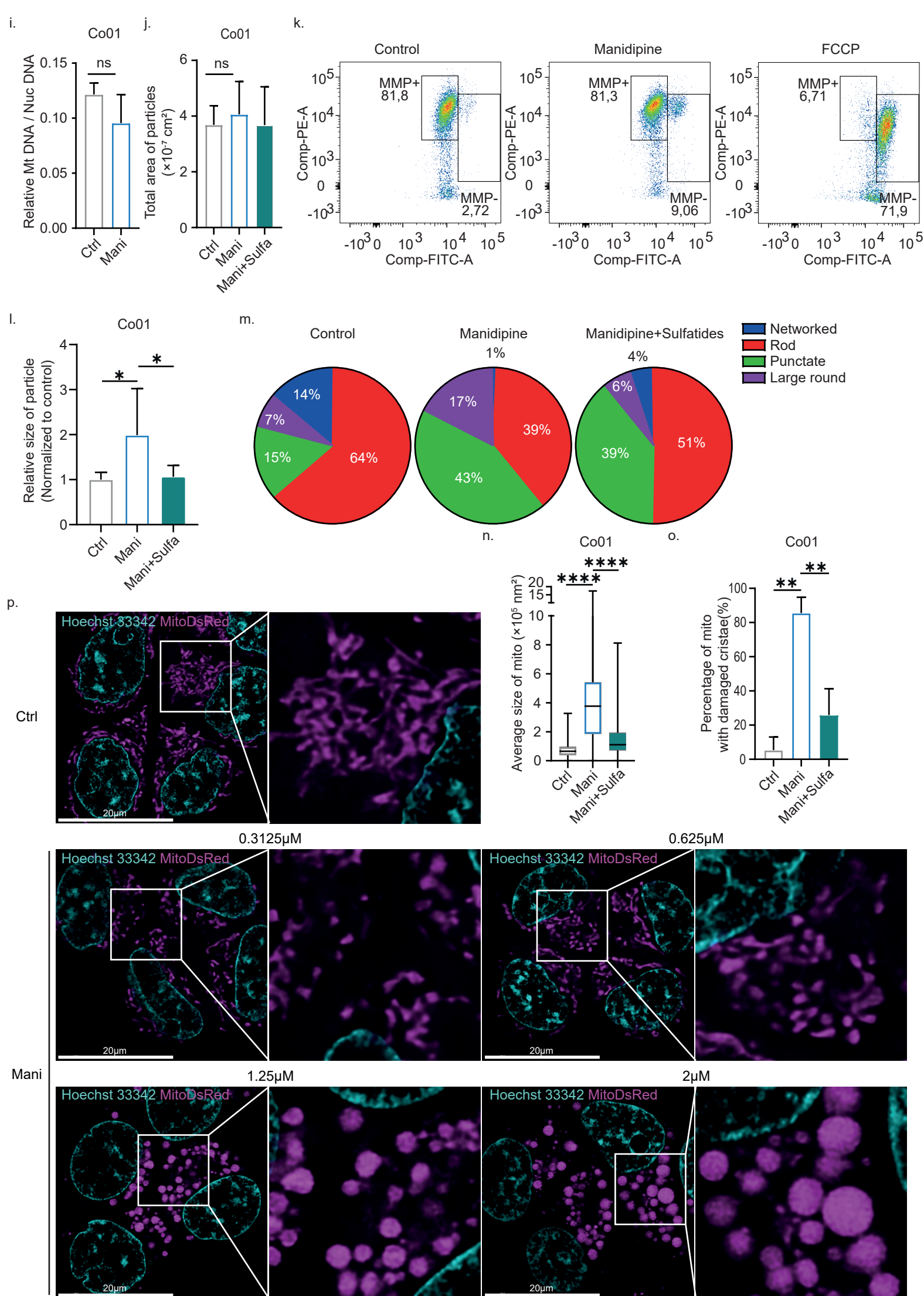

q.

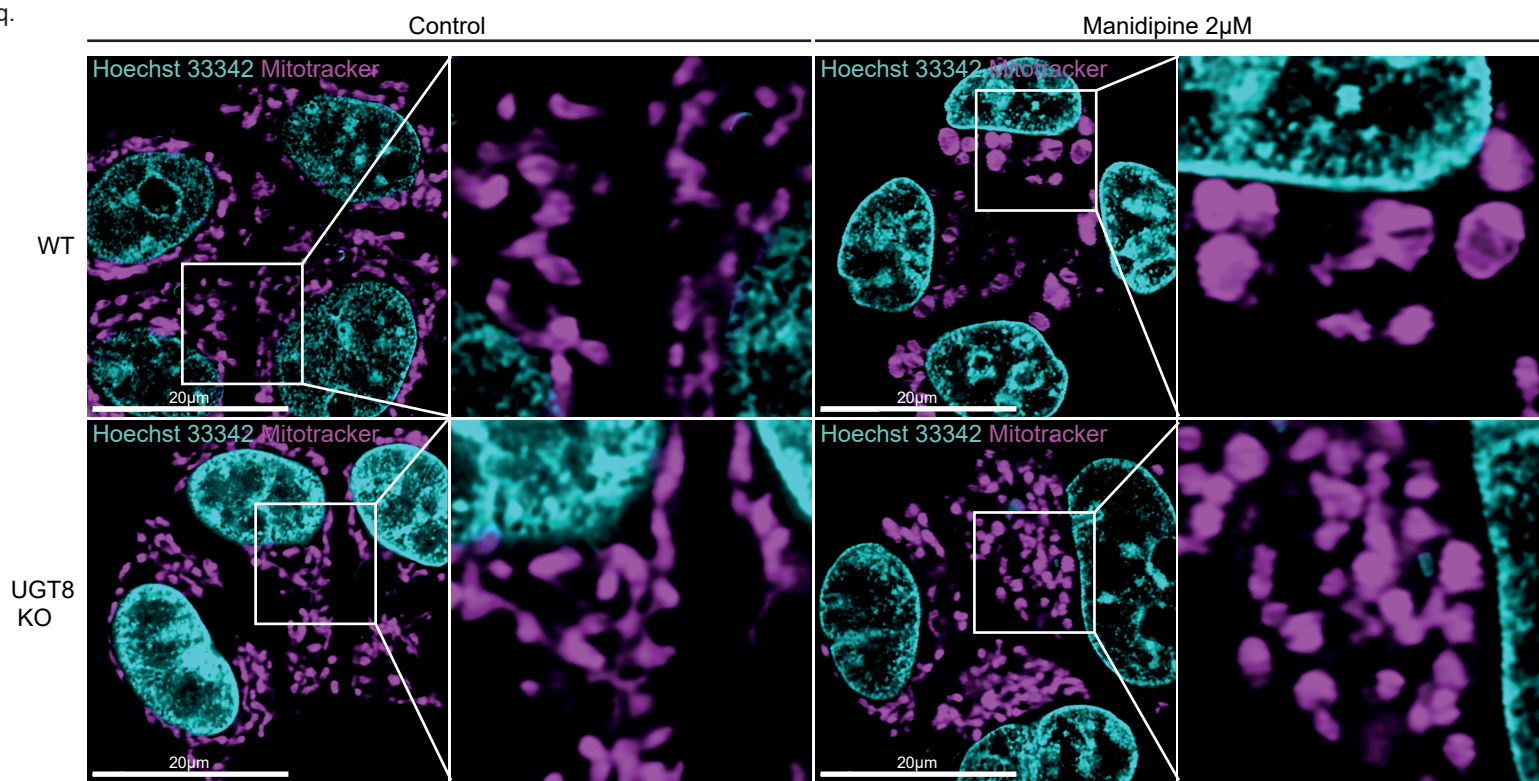

r.

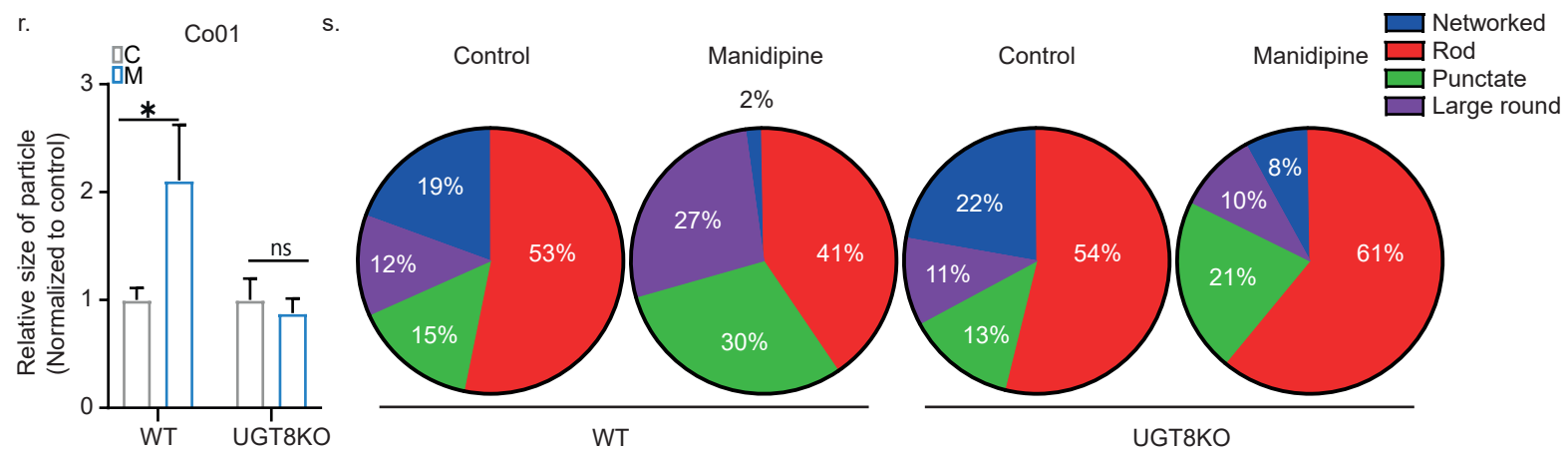

t.

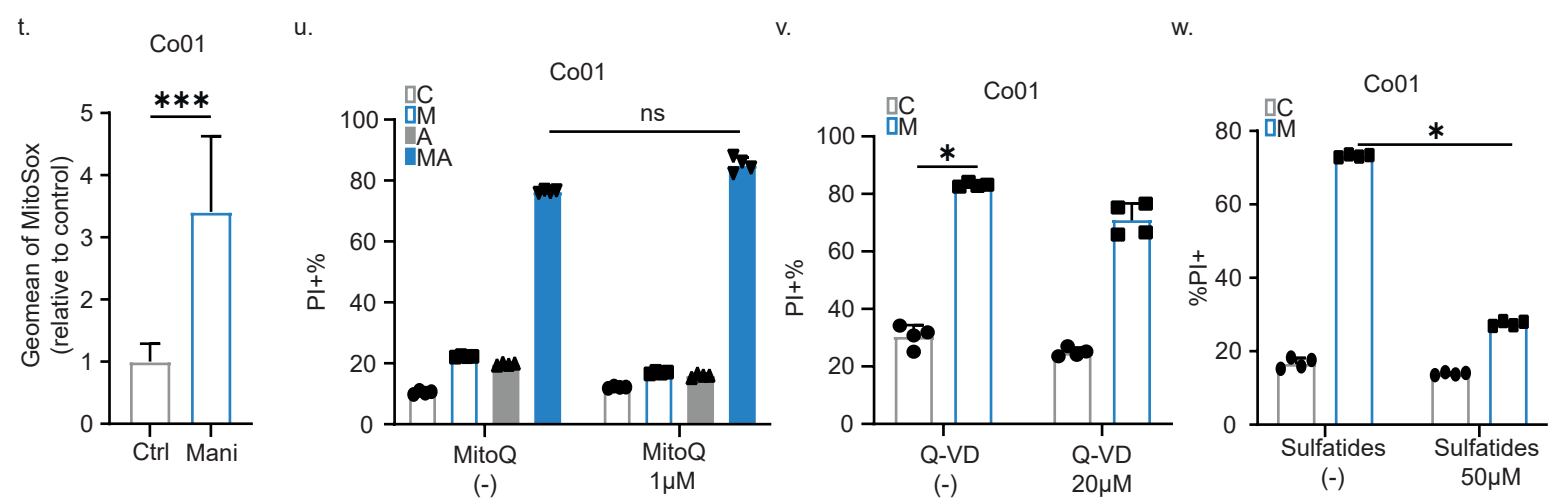

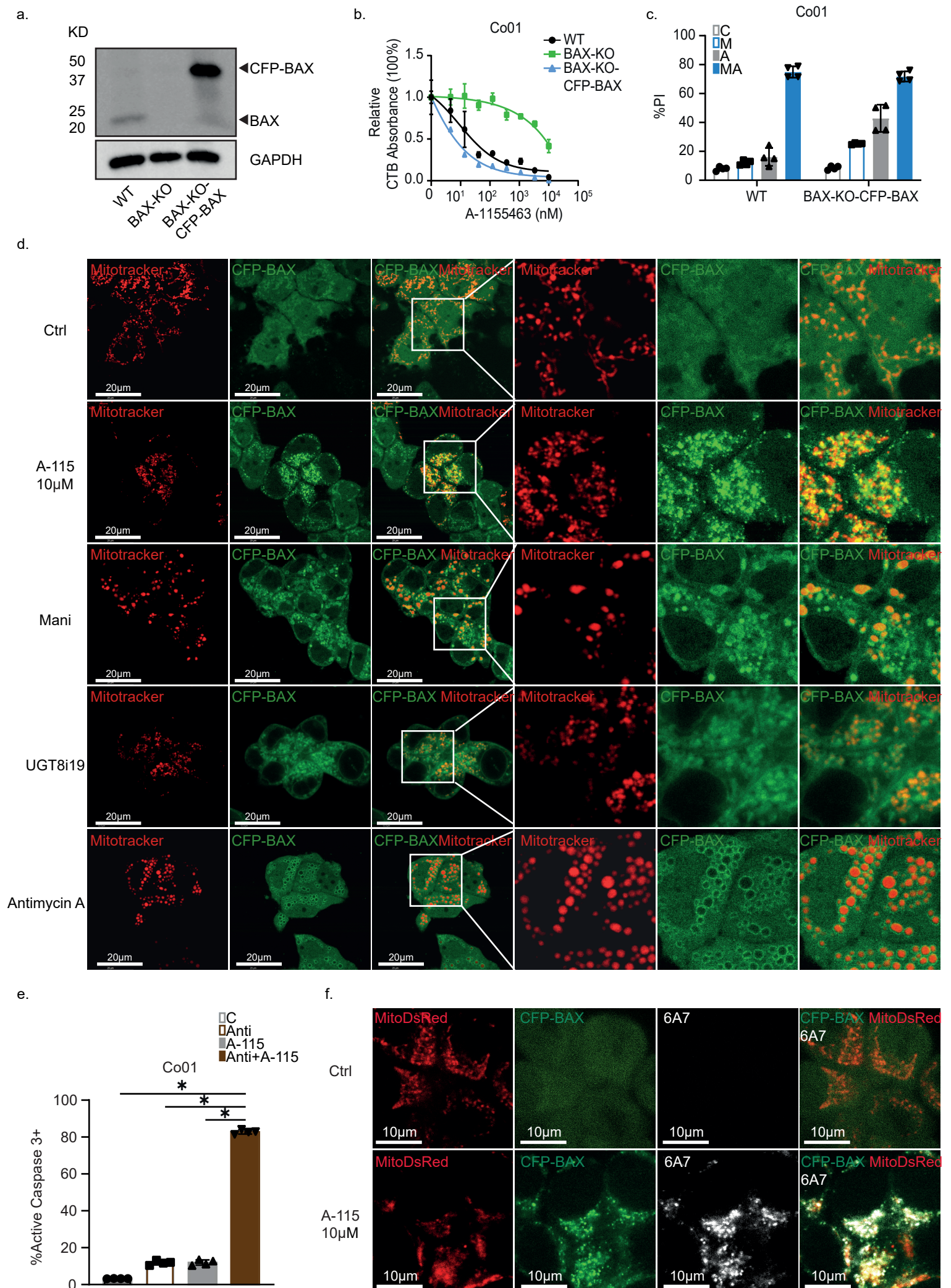

Supplementary Fig.5

a.

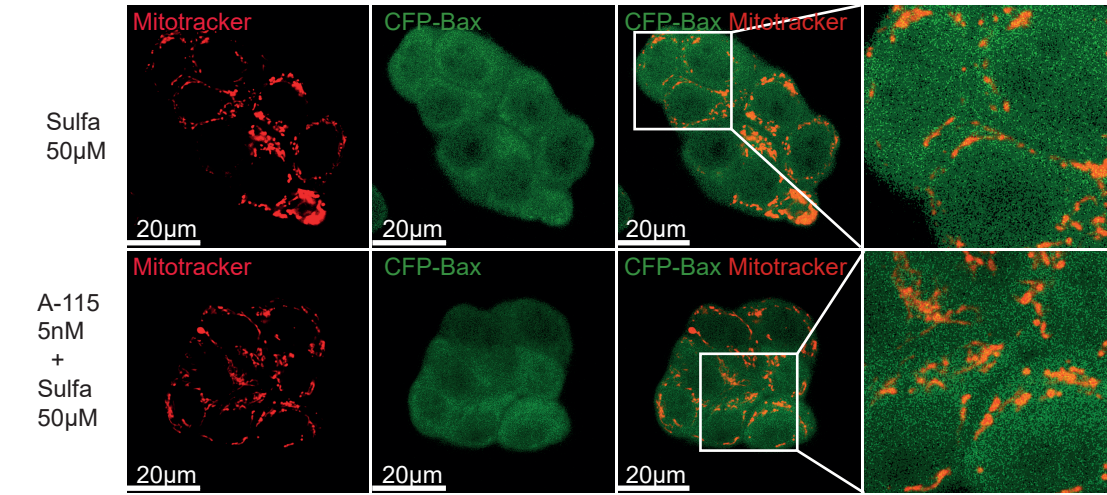

Supplement: Supplementary file 1 — Supplementary Figure 1-5 [file 41418_2024_1418_MOESM1_ESM.pdf]
