# Supplementary material for: UGT8 mediated sulfatide synthesis modulates BAX localization and dictates apoptosis sensitivity of colorectal cancer
Source: Cell Death Differ. 2024 Nov 23;32(4):657–71. doi: 10.1038/s41418-024-01418-y (PMC11982410; doi:10.1038/s41418-024-01418-y)
Supplement: Supplementary file 2 — Supplementary Figure Legends [file 41418_2024_1418_MOESM2_ESM.docx]

**Extended Fig. Legends**

**Extended Data Fig.1. Validation of the synergy between Manidipine-2HCl and A-1155463 in other CRC cells.**

**a)** Titration curve of Manidipine-2HCl with or without 5nM A-1155463. Relative CellTiter Blue absorbance to untreated control were calculated and plotted; n=3; **b)** Titration curve of A-1155463 with or without 2µM Manidpine-2HCl. Relative CellTiter Blue absorbance to untreated control were calculated and plotted; n=3; **c&d)** Heat map of the Bliss synergy score of the Manidipine-2HCl and A-1155463 in Co108(c) and Co147(d). Cell viability was measured by CellTiter Blue (n=4) and heat map of synergy scores were automatically calculated and plotted by Synergyfinder 3.0; **e&f)** Statistics of the percentage of Co01 with activated caspase 3 induced with or without 2.5µM Manidipine-2HCl and/or 5nM A-1155463 in Co108(e) while 1µM Manidipine-2HCl and/or 5nM A-1155463 in Co147 (f) after 48hrs, determined by flow cytometry; n=4. **g-q)** Percentage of cells showing caspase-3 activity upon treatment with or without corresponding doses of Manidipine-2HCl and/or A-1155463 after 48hrs, determined by flow cytometry. n=4; 5µM Manidipine for HT55 and HUTU-80, 10µM Manidipine for all the rest; 2.5µM A-1155463 for SW48, HCT-116, RCM-1, LS-1034 and NCI-H716; 5µM A-1155463 for OUMS-23; 10µM A-1155463 for all the rest; n=4-6, **r)** Percentage of PI positive HCT-116 in FSC complete (FSC(+)) or free (FSC(-)) condition, induced by 2μM Manidipine-2HCl and/or 5, 50nM A-1155463 after 48hrs, determined by flow cytometry. C: Control; M: Manidipine-2HCl 2µM; A 5nM: A-1155463 5nM; MA 5nM: Manidipine-2HCl 2µM+A-1155463 5nM; A 50nM: A-1155463 5nM; MA 50nM: Manidipine-2HCl 2µM+A-1155463 50nM; n=4;  **s&t)** Titration curve of BCL-2 inhibitor ABT-199(r) and MCL-1 inhibitor AZD5991(s) with or without 2µM Manidpine-2HCl. Relative CellTiter Blue absorbance to untreated control were calculated and plotted; n=3; Mann-Whitney test was performed to analyze the differences between two conditions. ∗: p ≤ 0.05;

**Extended Data Fig.2. Targeting UGT8 sensitizes CRC cells to low dose of A-1155463.**

**a)** Validation of the knockout of BAX and BAK in Co01-BAX/BAK SKO DKO cells. **b)** Percentage of PI positive Co01 induced by 2μM Manidipine-2HCl and/or 5nM A-1155463 in the presence or absence of 20μM Q-VD-Oph after 48hrs, determined by flow cytometry. C: Control; M: Manidipine-2HCl 2µM; A: A-1155463 5nM; MA: Manidipine-2HCl 2µM+A-1155463 5nM; n=4; **c)** mRNA expression of gene CaV1.1 in human CRC cell lines, the log2 transformed values were plotted according to the gene expression profile from R2 platform. Three lines, Co01, Co108 and Co147, used in this study were highlighted. **d&e**) Time course assessment of cytosolic Ca^2+^ (d) and mitochondrial Ca^2+^ (e) in Co01 cells treated with 2µM Manidipine. 30mins of 25mM EGTA treatment and 5mins of 10µM Ionomycin treatment were used as negative and positive control. **f-i)** Titration curve of four Ca^2+^ channel blockers with or without 5nM A-1155463. Relative CellTiter Blue absorbance to untreated control were calculated and plotted; n=3. j&k) Measurements of cytosolic Ca^2+^ (j) and mitochondrial Ca^2+^ (k) in Co01 cells treated with 1µM each dipine for 24hrs, 30mins of 25mM EGTA treatment and 5mins of 10µM Ionomycin treatment were used as negative and positive control. n=4. **l)** Illustrative scheme of the workflow of TPP assay. **m)** Melting curve of UGT8 treated with 1hr UGT8i19 generated by simplified TTP assay. . **n)** Sulfatides quantification by Lipidomic profiling on Co01 treated with or without 2µM UGT8i19 for 24hrs. The relative MS readout was normalized to the quantity of total input protein; n=5; **o)** Flow cytometry of galactosyl-ceramides (GalCer) in Co01 treated with or without 2µM UGT8i19 for 24hrs. Geometric means of the relative fluorescence of GalCer probe to mouse IgG isotype was plotted; n=4. **p)** Percentage of PI positivity of Co01 cells upon treatment with or without 2µM UGT8i19 and/or 5nM A-1155463 after 48hrs, determined by flow cytometry; n=4. **q)** TIDE analysis quantified the percentage of cells in the UGT8-KO cell that have certain number of base pair deletion or insertion at PAM of CRISPR Cas9 cutting site. **r)** Flow cytometry of galactosyl-ceramides (GalCer) in Co01 WT and UGT8-KO treated with or without 2µM Manidipine-2HCl or UGT8i19 for 24hrs. Geometric means of the relative fluorescence of GalCer probe to mouse IgG isotype was plotted; n=4. **s)** Titration curve of A-1155463 in Co01 WT and UGT8-KO lines. Relative CellTiter Blue absorbance to untreated control were calculated and plotted; n=3; **t)** TIDE analysis quantified the percentage of cells in the CST-KO cell that have certain number of base pair deletion or insertion at PAM of CRISPR Cas9 cutting site. **u)** Titration curve of A-1155463 in Co01 WT and CST-KO lines. Relative CellTiter Blue absorbance to untreated control were calculated and plotted; n=3; **v)** Percentage of PI positivity of Co01 WT and UGT8-KO cells upon treatment with or without 2µM Manidipine-2HCl UGT8i19 and/or 5nM A-1155463 after 48hrs, determined by flow cytometry; n=4. Significance was calculated with Mann-Whitney test. ∗: p ≤ 0.05.

**Extended Data Fig.3. Manidipine-2HCl sensitize to A-1155463 by targeting sulfatide synthesis.**

**a)** Lipidomic data of sulfatides species in Co01 treated with or without 50µM exogenous porcine brain-derived sulfatides. Red bar differentiate the species containing fatty acids bigger and smaller than 36 carbon. **b)** Lipidomic data of total, C34 and C42 sulfatides species in Co01 treated with or without 2µM Manidipine and /or 50µM exogenous porcine brain-derived sulfatides. C: Control, M: 2µM Manidipine. **c&d)** Statistics of the percentage of PI positive Co108(c) and Co147(d) induced by 2.5µM (Co108) or 2µM (Co147) Manidipine-2HCl and/or 5nM A-1155463 in the presence or absence of 50µM sulfatides after 48hrs, determined by flow cytometry. C: Control; M: Manidipine-2HCl; A: A-1155463; MA: Manidipine-2HCl+A-1155463; n=4. **e)** Percentage of PI positivity of Co01 cells induced by 2µM UGT8i19 and/or 5nM A-1155463 in the presence or absence of 50µM sulfatides after 48hrs, determined by flow cytometry. C: Control; U: UGT8i19 2µM; A: A-1155463 5nM; UA: UGT8i19 2µM+A-1155463 5nM; n=4. **f)** Validation of the composition of isolated heavy membrane fraction. The protein expression of ER marker (SERCA2), mitochondria marker (HSP60) and cytosol marker (GAPDH) in whole cell lysates, heavy membrane fraction containing mitochondrial (HM (Mito)) and cytosolic fraction (Cyto) of Co01 were presented. **g)** Oxygen consumption rate (OCR) of Co108 and Co147 treated with 2.5µM (Co108) or 2µM (Co147) Manidipine-2HCl with or without 50µM sulfatides were measured by seahorse mito stress assay. C: Control; M: Manidipine-2HCl; MS: Manidipine-2HCl+sulfatides; n=4. **h)** Oxygen consumption rate (OCR) of Co01 treated with 5nM A-1155463 or 2.5µM Manidipine-2HCl were measured by seahorse mito stress assay. C: Control; A:A-1155463; M:Manidipine-2HCl; n=4. **i)** Relative copy number of mitochondrial DNA (MtDNA) to nuclear DNA (Nuc DNA) was determined by quantitative PCR in Co01 treated with or without 2µM Manidipine-2HCl (Mani) for 24hrs . **j)** Total particle area of mitochondria calculated according to confocal MitoDsRed images of Co01 treated with or without 2µM Manidipine-2HCl (Mani) with 50µM sulfatides (Mani+Sulfa) or without for 24hrs. **k)** JC-1 staining of Co01 cell treated with 2µM Manidipine-2HCl for 24hrs or 2µM FCCP for 30mins. Mono form (FITC) and aggregate form (PE) of JC-1 were measured by FACS. **l)** Quantification of the particle size of the MitoDsRed of Co01 treated with or without 2µM Manidipine-2HCl (Mani) with 50µM sulfatides (Mani+Sulfa) or without for 24hrs. **m)** Distribution of 4 types of mitochondria stratified by shapes were plotted according to confocal MitoDsRed images of Co01 treated with or without 2µM Manidipine-2HCl (Mani) with 50µM sulfatides (Mani+Sulfa) or without for 24hrs. **n)** Quantification of the mitochondrial size of Co01 treated with with or without 2µM Manidipine-2HCl (Mani) with 50µM sulfatides (Mani+Sulfa) or without for 24hrs, according to EM images. **o)** Percentage of the mitochondria with damaged cristae in Co01 treated with with or without 2µM Manidipine-2HCl (Mani) with 50µM sulfatides (Mani+Sulfa) or without for 24hrs, according to EM images. **p)** The mitochondrial morphology was determined with mitochondria targeting DsRed (MitoDsRed) after treatment with 0, 0.3125, 0.625, 1.25 and 2μM Manidipine-2HCl. Mitochondria were imaged with confocal microscope at 63× magnification. **q)** The mitochondrial morphology of WT and UGT8-KO Co01 was determined with Mitotracker Deep Red after treatment with or without 2μM Manidipine-2HCl. Mitochondria were imaged with confocal microscope at 63× magnification. **r)** Quantification of the particle size of the Mitotracker Deep Red particle of WT and UGT8-KO Co01 treated with or without 2µM Manidipine-2HCl for 24hrs, C: control, M: Manidipine-2HCl. **s)** Distribution of 4 types of mitochondria stratified by shapes were plotted according to confocal Mitotracker Deep Red images of WT and UGT8-KO Co01 treated with or without 2µM Manidipine-2HCl for 24hrs. **t)** Mitochindrial ROS production of Co01 treated with or without 2μM Manidipine-2HCl (Mani) for 24hrs was determined by MitoSOX flow cytometry. **u)** Statistics of the percentage of PI positive Co01 induced by 2µM Manidipine-2HCl and/or 5nM A-1155463 in the presence or absence of 1µM mitoquinone mesylate (MitoQ) after 48hrs, determined by flow cytometry. C: Control; M: Manidipine-2HCl; A: A-1155463; MA: Manidipine-2HCl+A-1155463; n=4. **v&w)** Statistics of the percentage of PI positive Co01 induced by 2µM Manidipine-2HCl in the presence or absence of 20µM Q-VD-Oph(v) or 50µM sulfatides(w) after 5 days, determined by flow cytometry. C: Control; M: Manidipine-2HCl; Significance was calculated with Mann-Whitney test. ns: not significant, ∗:p≤ 0.05, ∗∗: p ≤ 0.01, ∗∗∗:p≤ 0.001, ∗∗∗∗: p ≤ 0.0001;

**Extended Data Fig.4. UGT8i19 regulates BAX localization.**

**a)** Western blot for BAX and CFP-BAX in wild type (WT) BAX-KO (BAK-KO) and BAX-KO-CFP-BAX Co01. BAX is 20KD while CFP is 26KD, thus CFP-BAX is 46KD. **b)** Titration curve of BCL-XL inhibitor A-1155463 in wild type (WT) BAX-KO (BAK-KO) and BAX-KO-CFP-BAX Co01. Relative CellTiter Blue (n=4) absorbance to untreated control were calculated and plotted; **c)** Percentage of PI positive Co01-wild type (WT) and Co01-BAX-KO-CFP-BAX induced by 2μM Manidipine-2HCl and/or 5nM A-1155463 after 48hrs, determined by flow cytometry. C: Control; M: Manidipine-2HCl 2µM; A: A-1155463 5nM; MA: Manidipine-2HCl 2µM+A-1155463 5nM; n=4; **d)** CFP-BAX aggregation were imaged with confocal microscope at 63× magnification. Co01-BAX-KO-CFP-BAX were treated with 10µM A-1155463 for 3hrs or 2µM Manidipine-2HCl/2µM UGT8i19/2 µM Antimycin A for 24hrs before 50nM Mitotracker Deep Red was used to stain mitochondria. **e)** Percentage of active caspase 3 positive Co01 induced by 2μM Antimycin A and/or 5nM A-1155463 after 48hrs, determined by flow cytometry. C: Control; Anti: 2µM; A-115: A-1155463 5nM; Anti+A-115: Antimycin A 2µM+A-1155463 5nM; n=4; **f)** Active BAX immunofluorescent staining with mouse anti-BAX monoclonal antibody 6A7 clone. Co01-BAXKO-CFP-BAX-MitoDsRed were treated with or without 10μM A-1155463 for 3hrs before fixation. Images were obtained with confocal microscope at 63× magnification;

**Extended Data Fig.5. Sulfatides regulates BAX localization.**

**a)** CFP-BAX aggregation was imaged with confocal microscope at 63× magnification. Co01-BAX-KO-CFP-BAX were pre-treated with 50µM sulfatides with or without 5nM A-1155463 for 3hrs before 50nM Mitotracker Deep Red was used to stain mitochondria.
