# Supplementary material for: UGT8 mediated sulfatide synthesis modulates BAX localization and dictates apoptosis sensitivity of colorectal cancer
Source: Cell Death Differ. 2024 Nov 23;32(4):657–71. doi: 10.1038/s41418-024-01418-y (PMC11982410; doi:10.1038/s41418-024-01418-y)
Supplement: Supplementary file 9 — Supplementary Data 1 [file 41418_2024_1418_MOESM9_ESM.pdf]

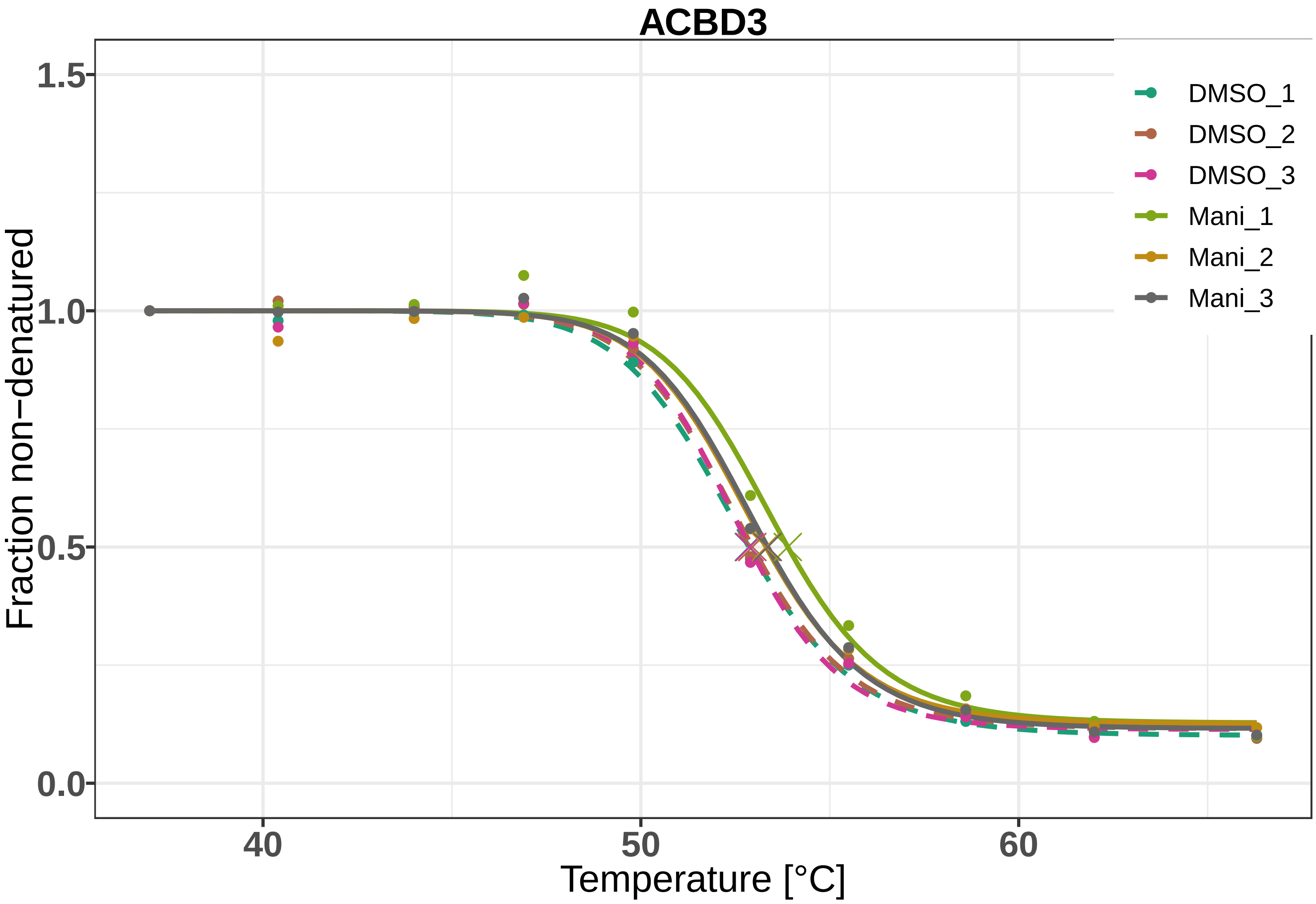

	meltPoint	slope	plateau	R2
DMSO_1	52.86	−0.14	0.1	1
DMSO_2	52.95	−0.15	0.12	1
DMSO_3	52.88	−0.16	0.11	1
Mani_1	53.89	−0.15	0.13	0.99
Mani_2	53.31	−0.15	0.13	1
Mani_3	53.36	−0.15	0.12	1

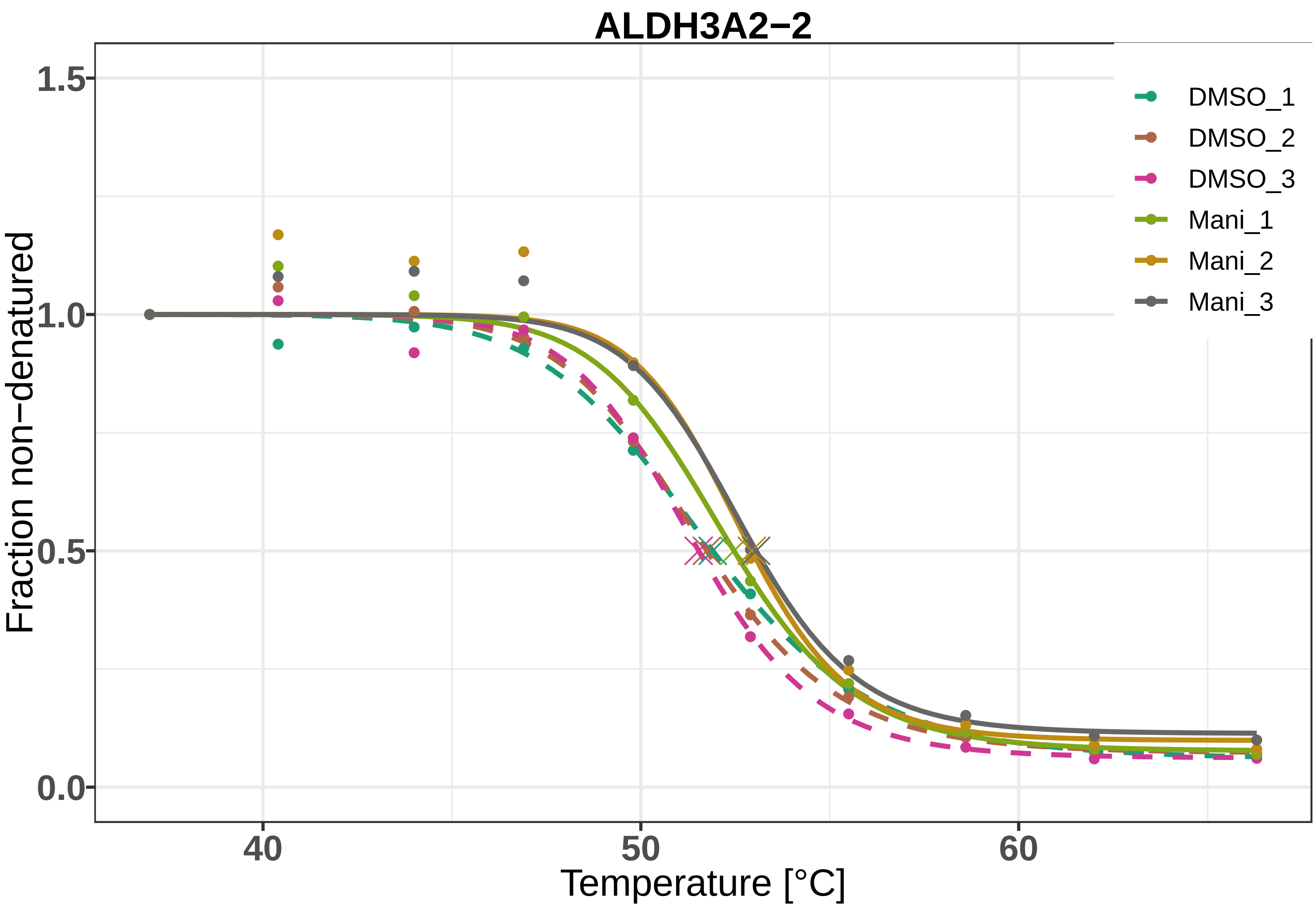

	meltPoint	slope	plateau	R2
DMSO_1	51.9	−0.11	0.06	1
DMSO_2	51.75	−0.13	0.07	1
DMSO_3	51.53	−0.14	0.06	1
Mani_1	52.46	−0.13	0.08	0.99
Mani_2	52.94	−0.16	0.1	0.97
Mani_3	53.05	−0.15	0.11	0.99

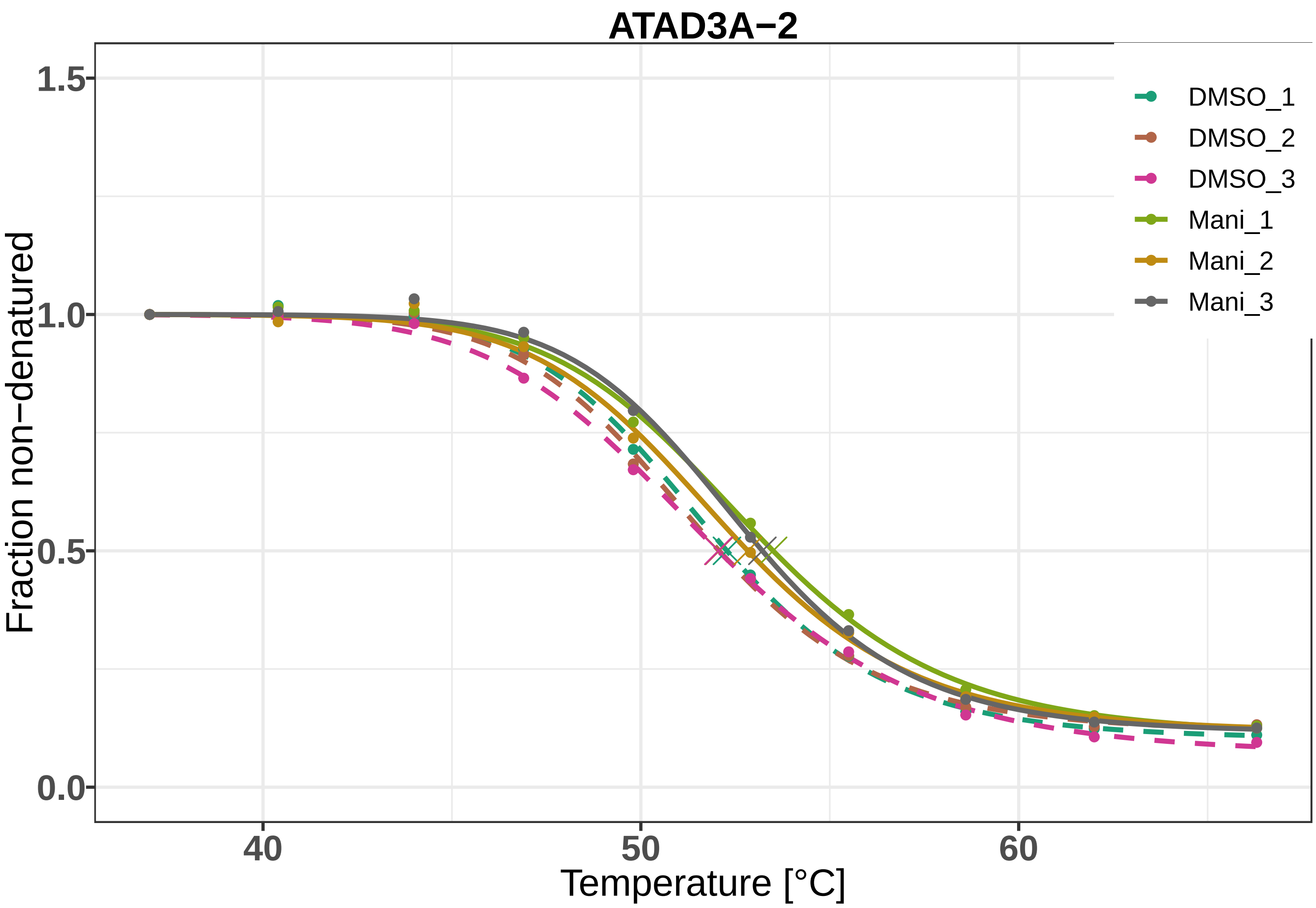

	meltPoint	slope	plateau	R2
DMSO_1	52.28	−0.096	0.1	1
DMSO_2	52.07	−0.093	0.12	1
DMSO_3	52.05	−0.082	0.07	1
Mani_1	53.5	−0.085	0.11	1
Mani_2	52.83	−0.088	0.12	1
Mani_3	53.22	−0.098	0.12	1

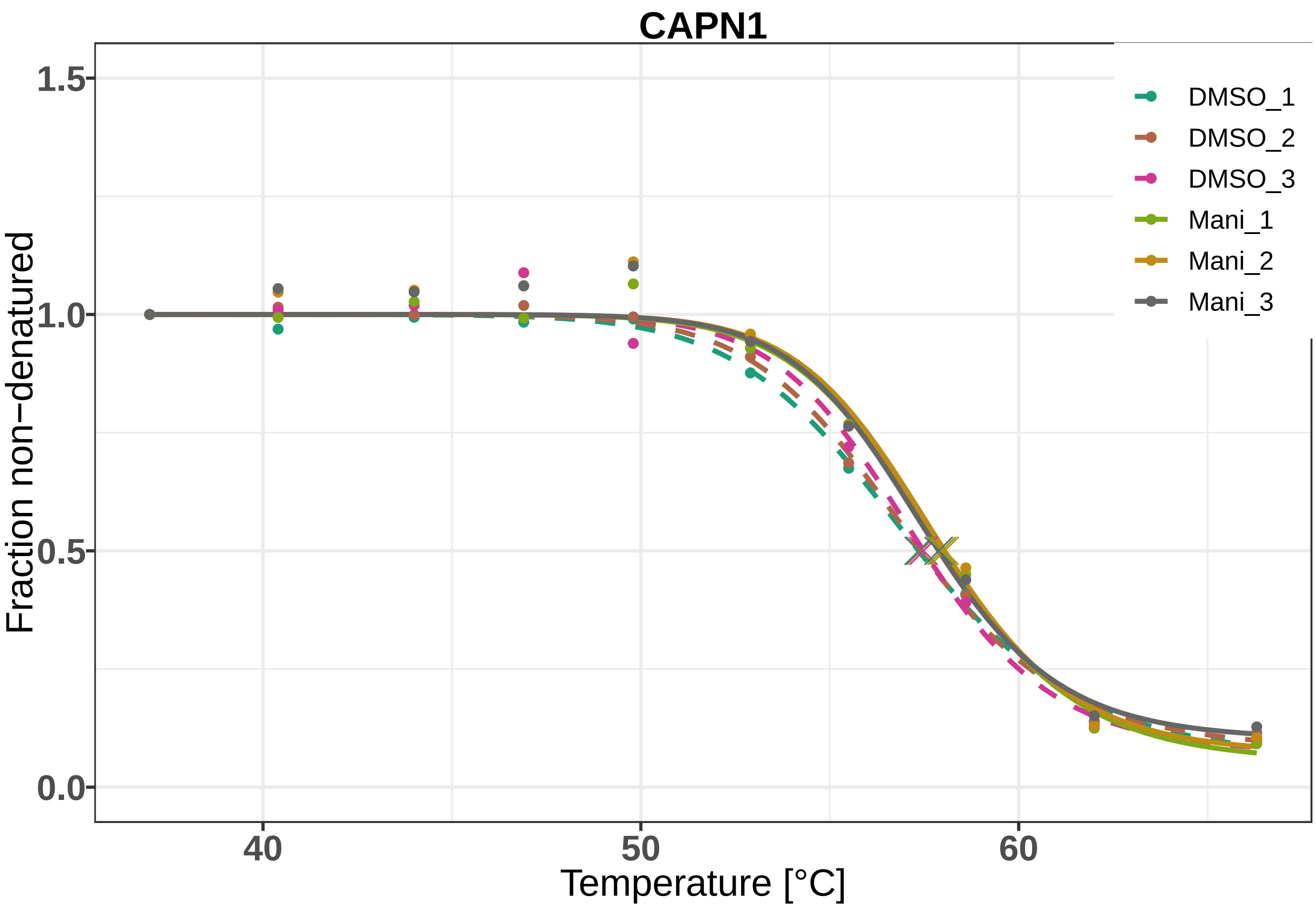

	meltPoint	slope	plateau	R2
DMSO_1	57.35	−0.1	0.06	1
DMSO_2	57.4	−0.11	0.08	1
DMSO_3	57.48	−0.12	0.07	0.99
Mani_1	57.96	−0.12	0.05	0.99
Mani_2	58.04	−0.13	0.07	0.98
Mani_3	57.88	−0.13	0.1	0.98

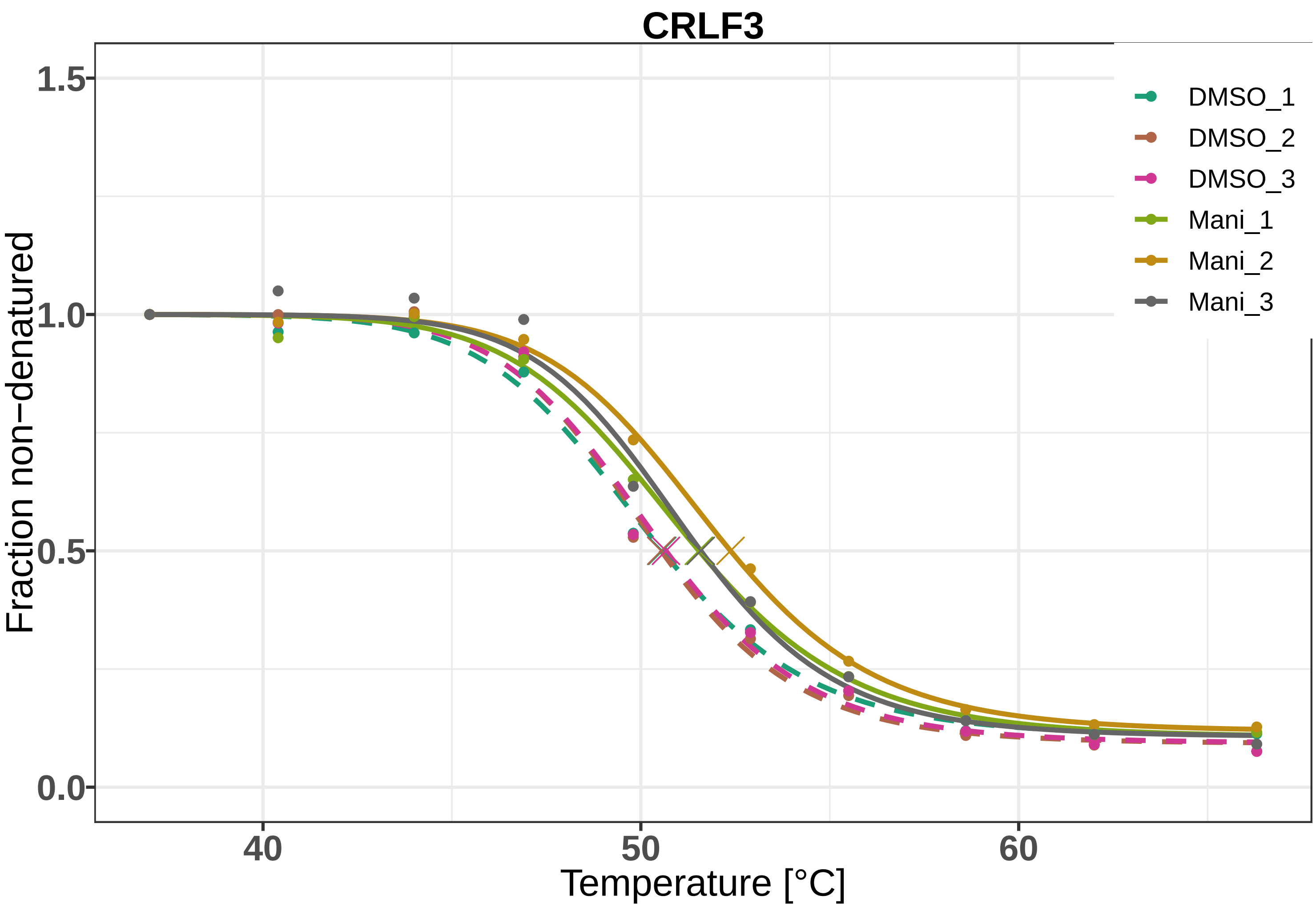

	meltPoint	slope	plateau	R2
DMSO_1	50.57	−0.1	0.11	1
DMSO_2	50.54	−0.12	0.09	0.99
DMSO_3	50.67	−0.11	0.09	0.99
Mani_1	51.54	−0.099	0.11	1
Mani_2	52.37	−0.1	0.12	1
Mani_3	51.59	−0.11	0.11	0.99

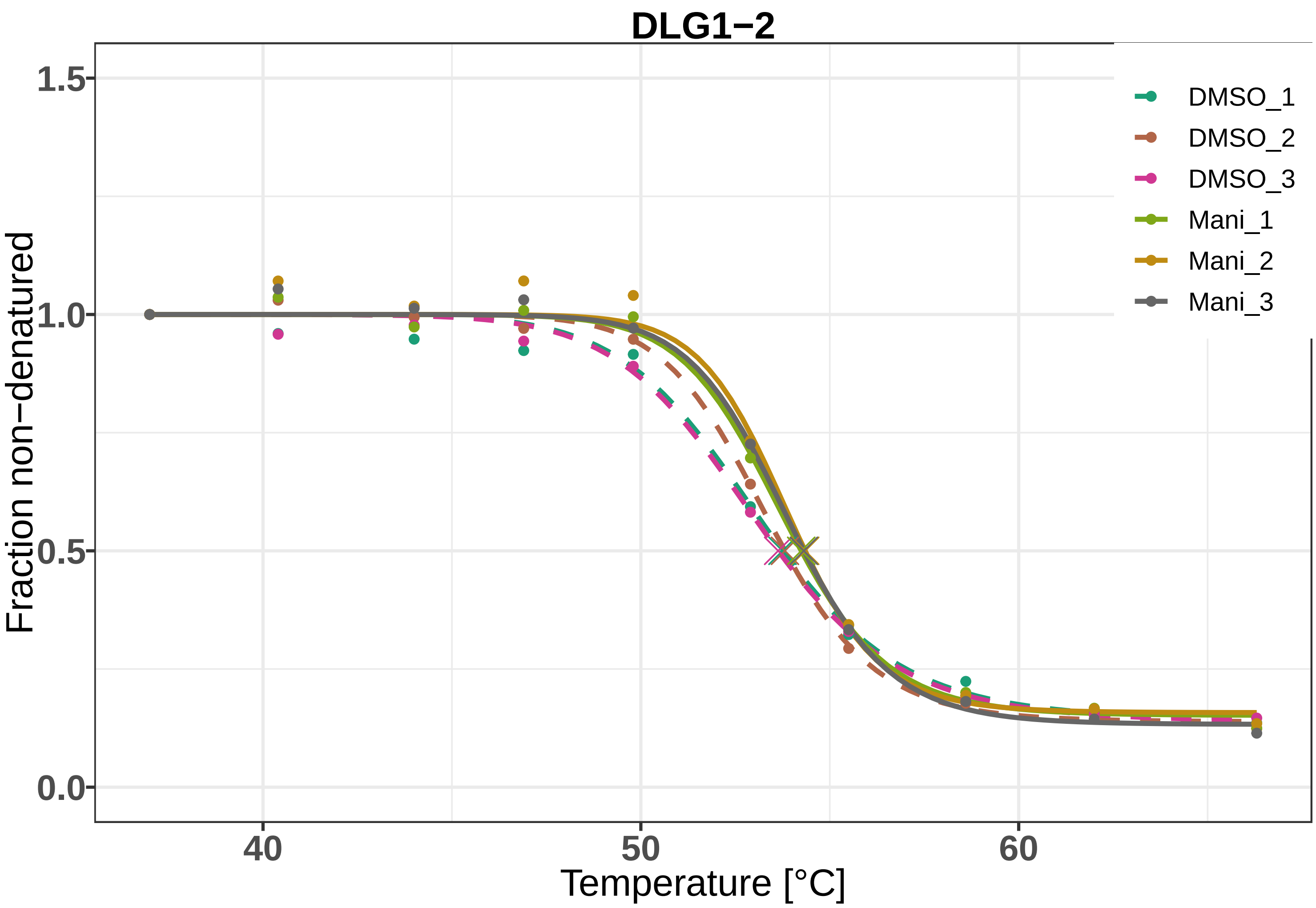

	meltPoint	slope	plateau	R2
DMSO_1	53.75	−0.12	0.14	0.99
DMSO_2	53.81	−0.15	0.14	1
DMSO_3	53.63	−0.11	0.14	1
Mani_1	54.24	−0.16	0.15	1
Mani_2	54.35	−0.18	0.16	0.99
Mani_3	54.32	−0.16	0.13	1

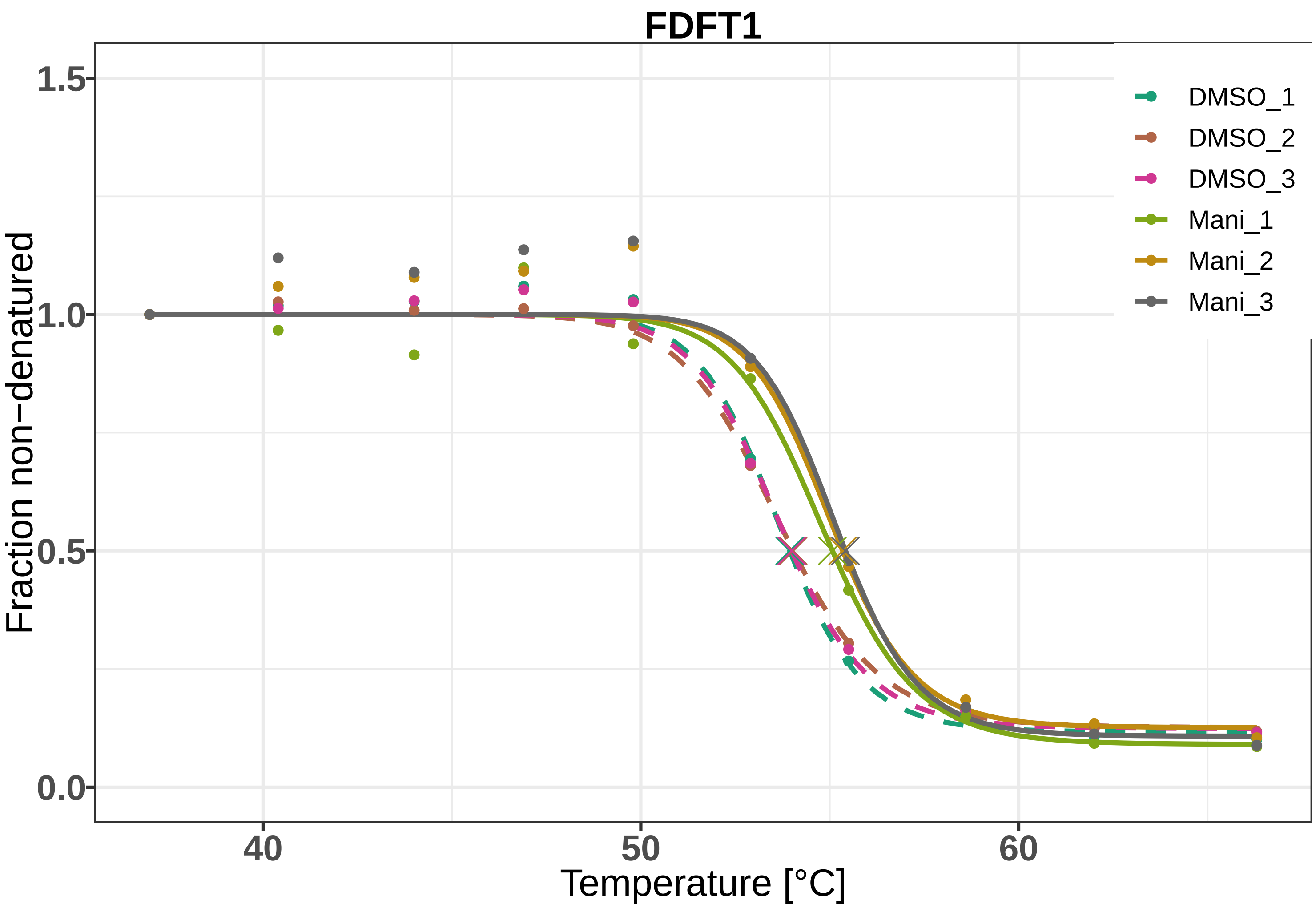

	meltPoint	slope	plateau	R2
DMSO_1	53.94	−0.2	0.12	1
DMSO_2	54.03	−0.17	0.13	1
DMSO_3	53.99	−0.19	0.12	1
Mani_1	55.07	−0.19	0.09	0.99
Mani_2	55.34	−0.2	0.13	0.98
Mani_3	55.42	−0.21	0.11	0.96

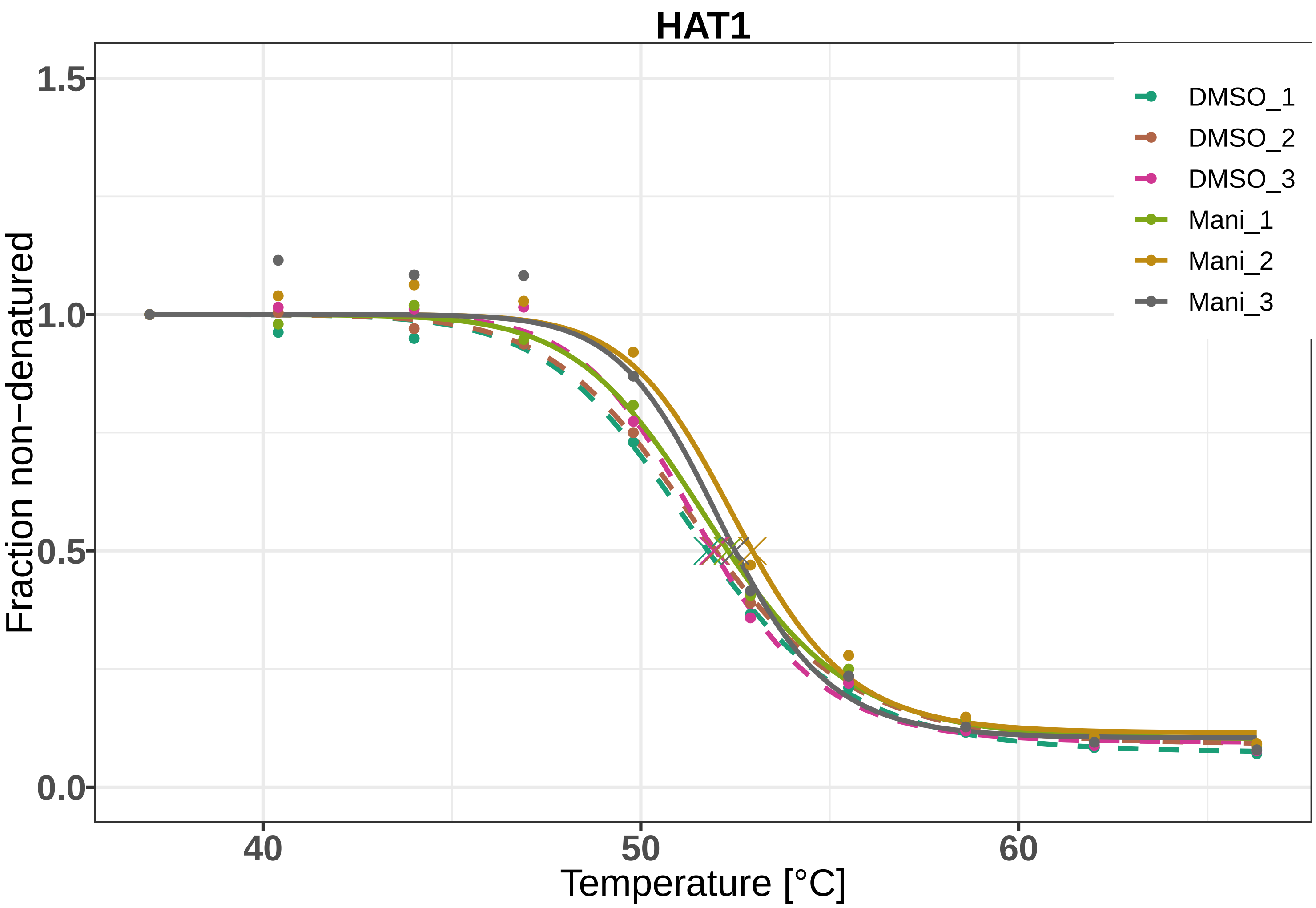

	meltPoint	slope	plateau	R2
DMSO_1	51.77	−0.12	0.07	1
DMSO_2	51.98	−0.11	0.09	1
DMSO_3	51.93	−0.14	0.09	1
Mani_1	52.31	−0.12	0.1	1
Mani_2	52.95	−0.15	0.11	0.99
Mani_3	52.49	−0.16	0.1	0.98

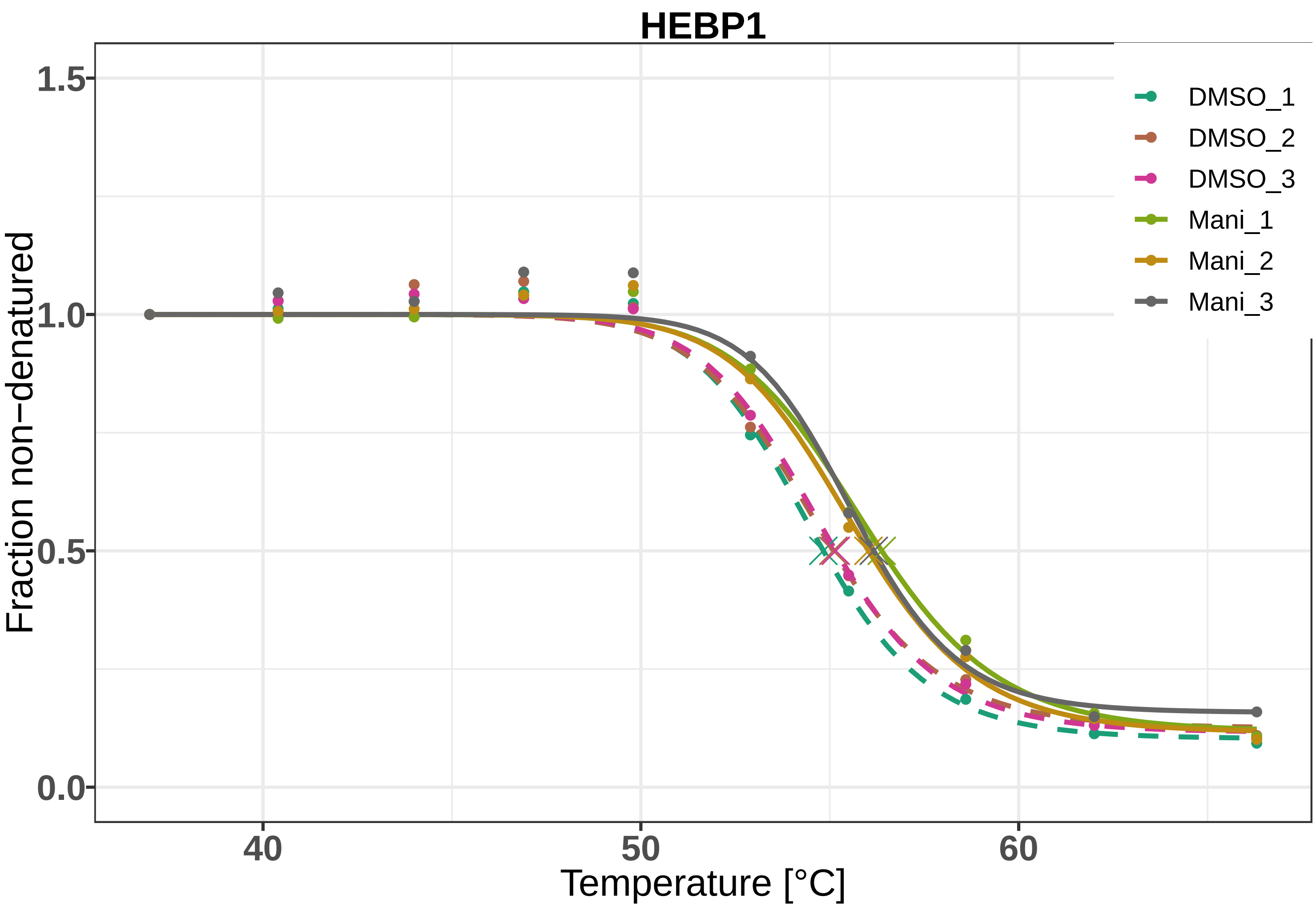

	meltPoint	slope	plateau	R2
DMSO_1	54.83	−0.15	0.1	1
DMSO_2	55.09	−0.13	0.12	0.99
DMSO_3	55.16	−0.14	0.12	1
Mani_1	56.37	−0.13	0.12	0.99
Mani_2	56.02	−0.13	0.12	0.99
Mani_3	56.16	−0.15	0.16	0.98

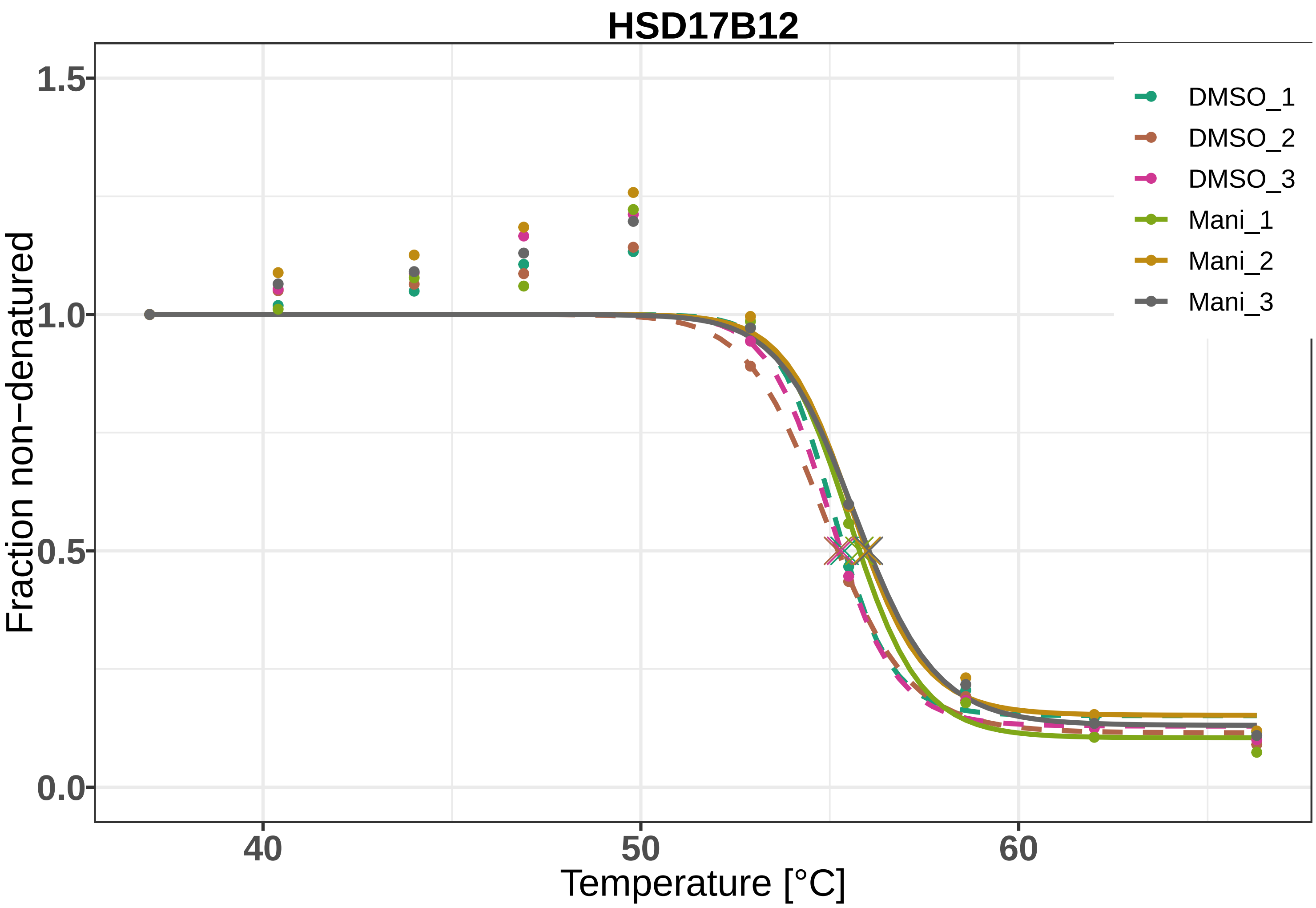

	meltPoint	slope	plateau	R2
DMSO_1	55.39	−0.28	0.15	0.98
DMSO_2	55.22	−0.21	0.11	0.98
DMSO_3	55.3	−0.26	0.13	0.96
Mani_1	55.78	−0.24	0.1	0.97
Mani_2	55.97	−0.23	0.15	0.93
Mani_3	56.04	−0.21	0.13	0.96

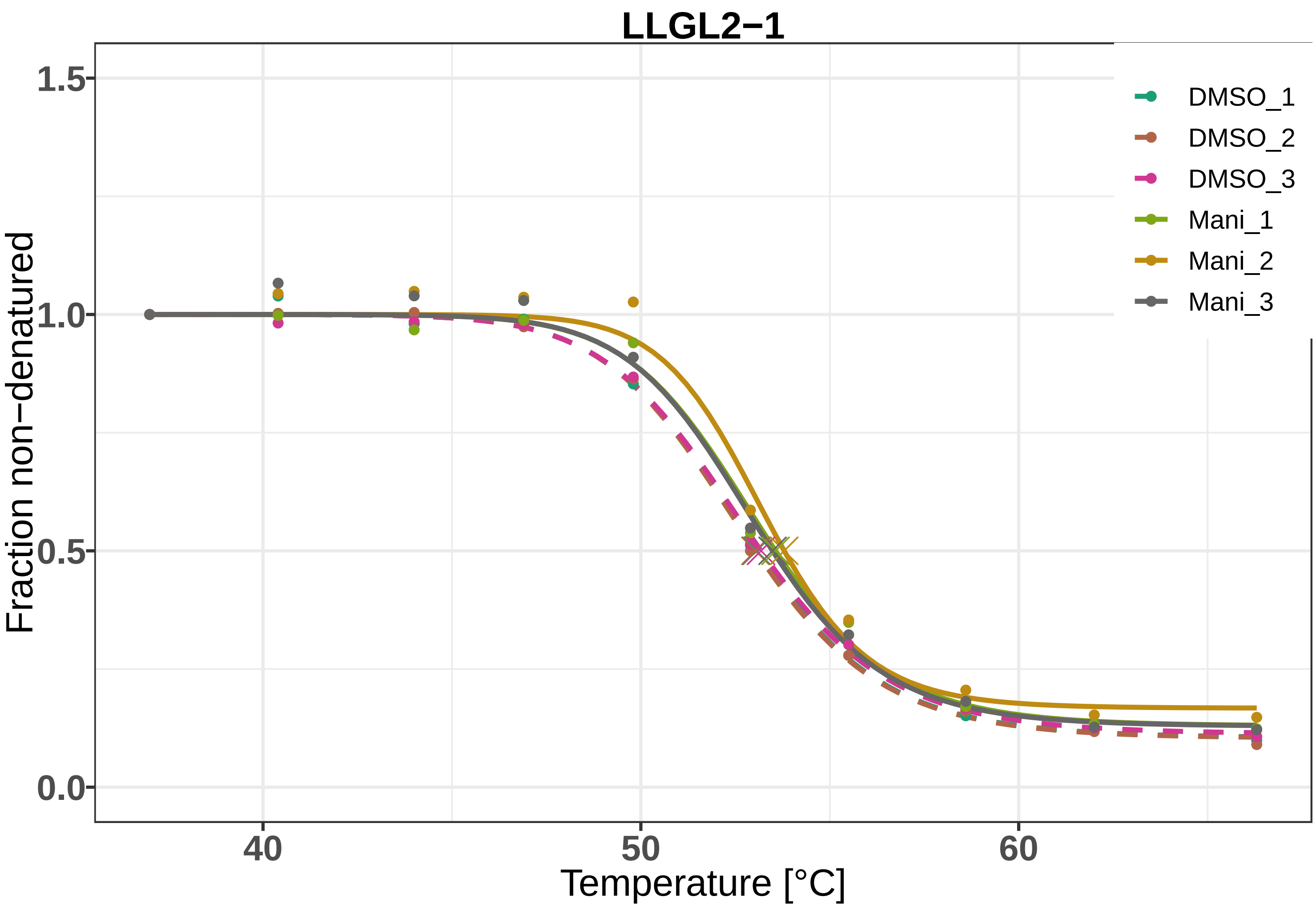

	meltPoint	slope	plateau	R2
DMSO_1	53.06	−0.12	0.1	1
DMSO_2	53.04	−0.12	0.1	1
DMSO_3	53.18	−0.12	0.11	1
Mani_1	53.56	−0.13	0.13	1
Mani_2	53.79	−0.15	0.17	0.99
Mani_3	53.49	−0.13	0.13	0.99

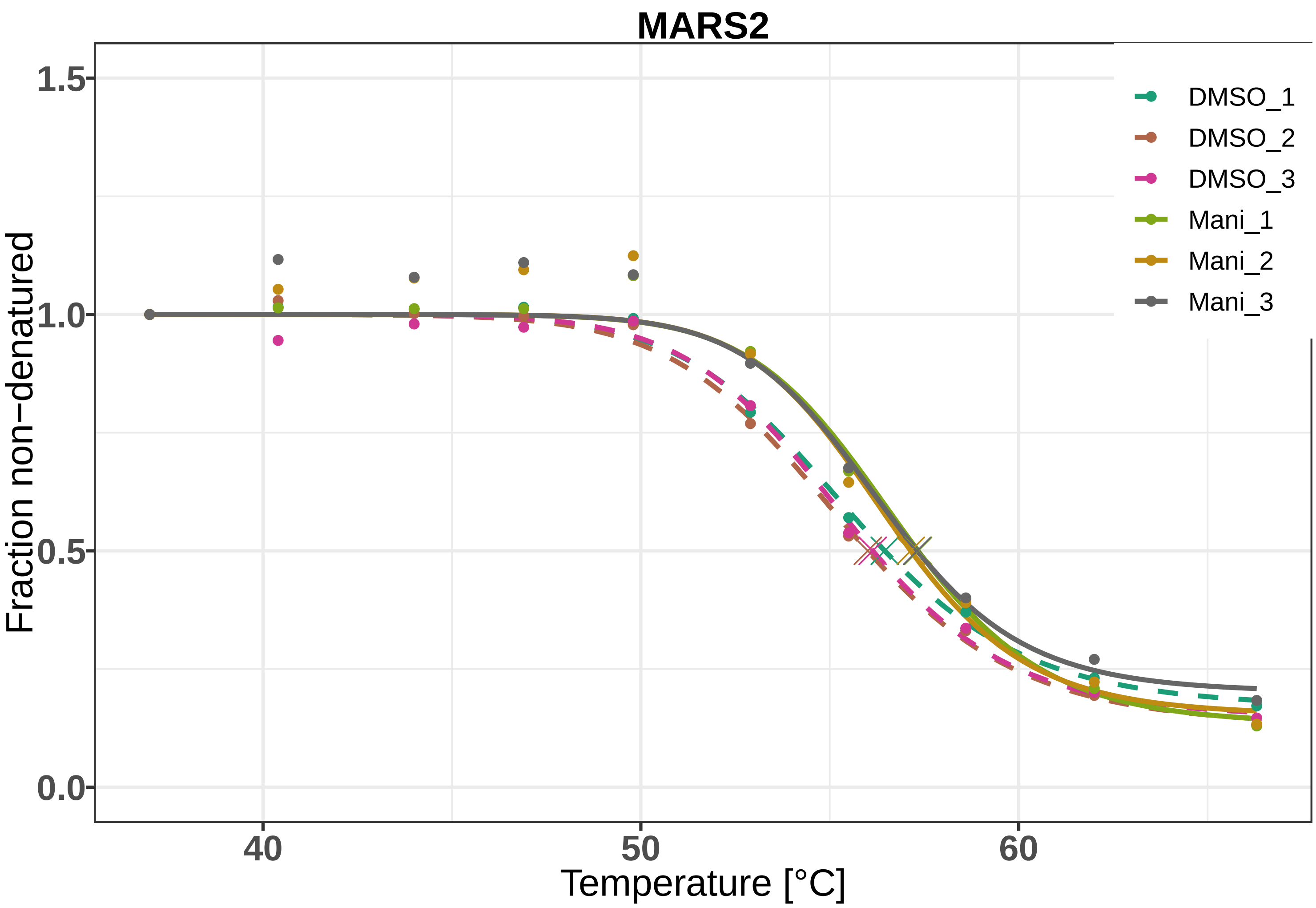

	meltPoint	slope	plateau	R2
DMSO_1	56.46	−0.092	0.17	1
DMSO_2	56.01	−0.095	0.13	1
DMSO_3	56.14	−0.1	0.15	0.99
Mani_1	57.34	−0.11	0.13	0.99
Mani_2	57.14	−0.12	0.15	0.97
Mani_3	57.31	−0.11	0.2	0.96

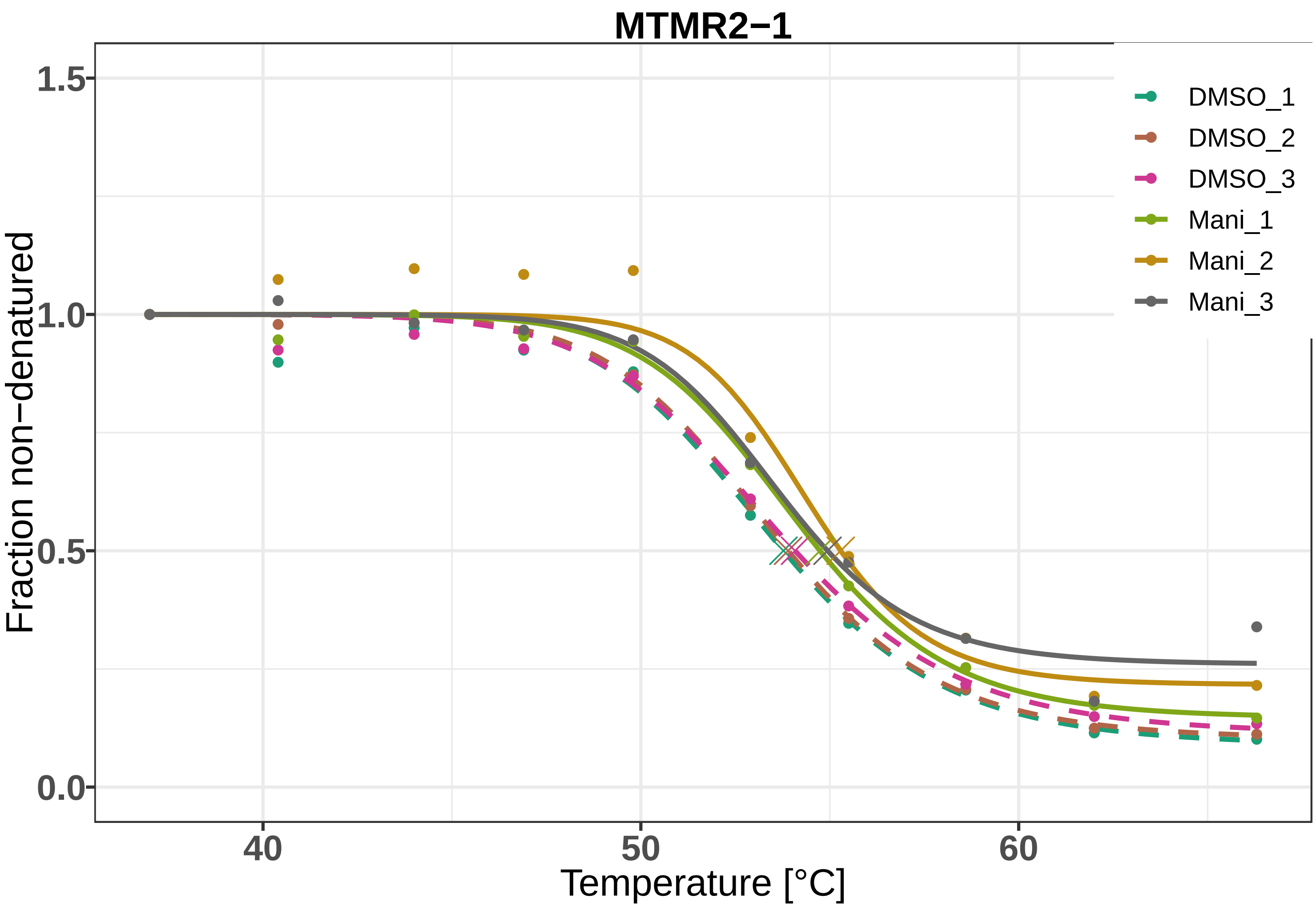

	meltPoint	slope	plateau	R2
DMSO_1	53.77	−0.098	0.09	0.99
DMSO_2	53.9	−0.1	0.1	1
DMSO_3	54.08	−0.092	0.11	0.99
Mani_1	54.74	−0.11	0.15	1
Mani_2	55.29	−0.13	0.22	0.97
Mani_3	54.94	−0.1	0.26	0.98

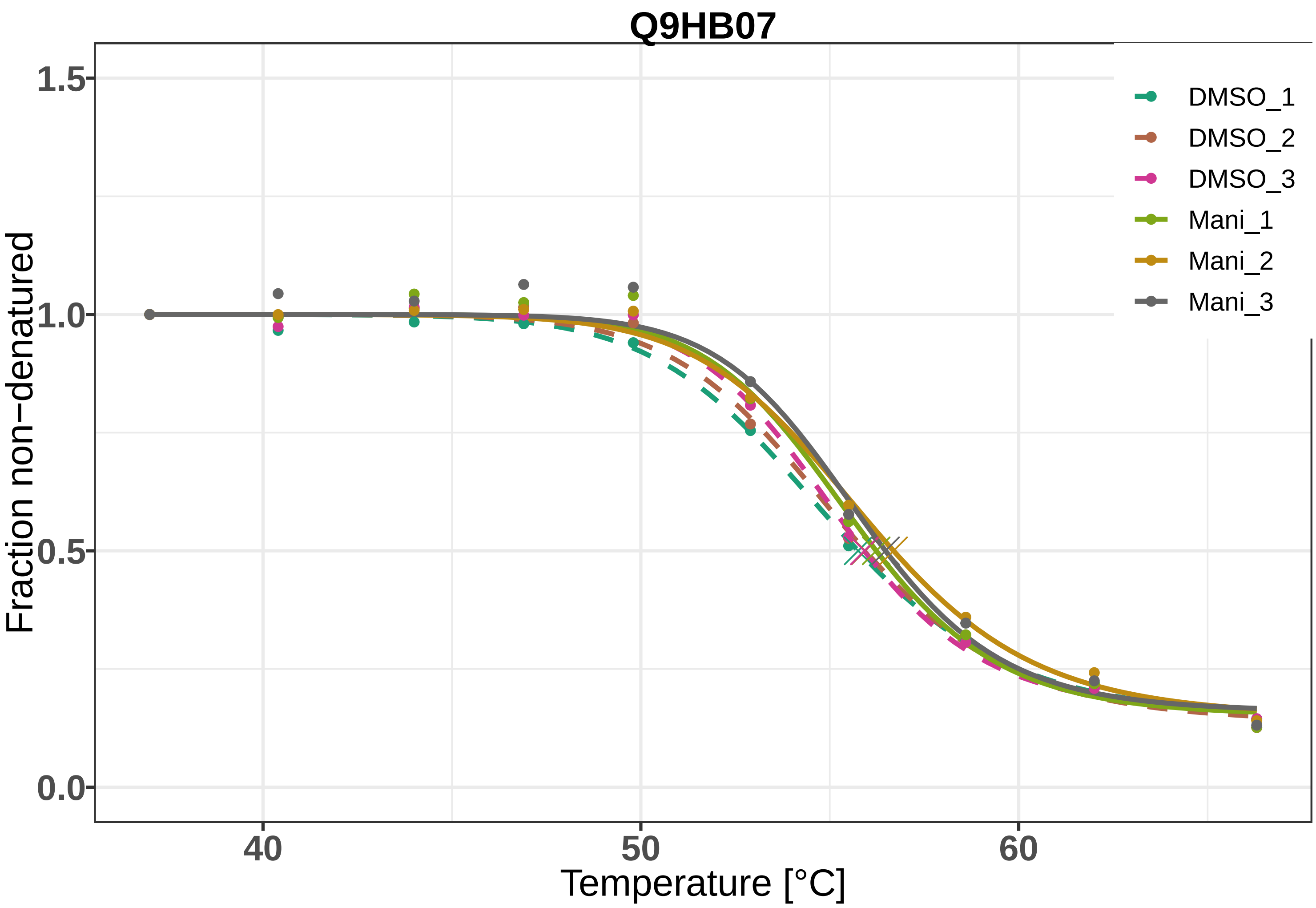

	meltPoint	slope	plateau	R2
DMSO_1	55.75	−0.091	0.14	1
DMSO_2	55.94	−0.097	0.14	1
DMSO_3	55.91	−0.11	0.16	1
Mani_1	56.23	−0.11	0.15	0.99
Mani_2	56.69	−0.096	0.15	1
Mani_3	56.47	−0.11	0.16	0.99

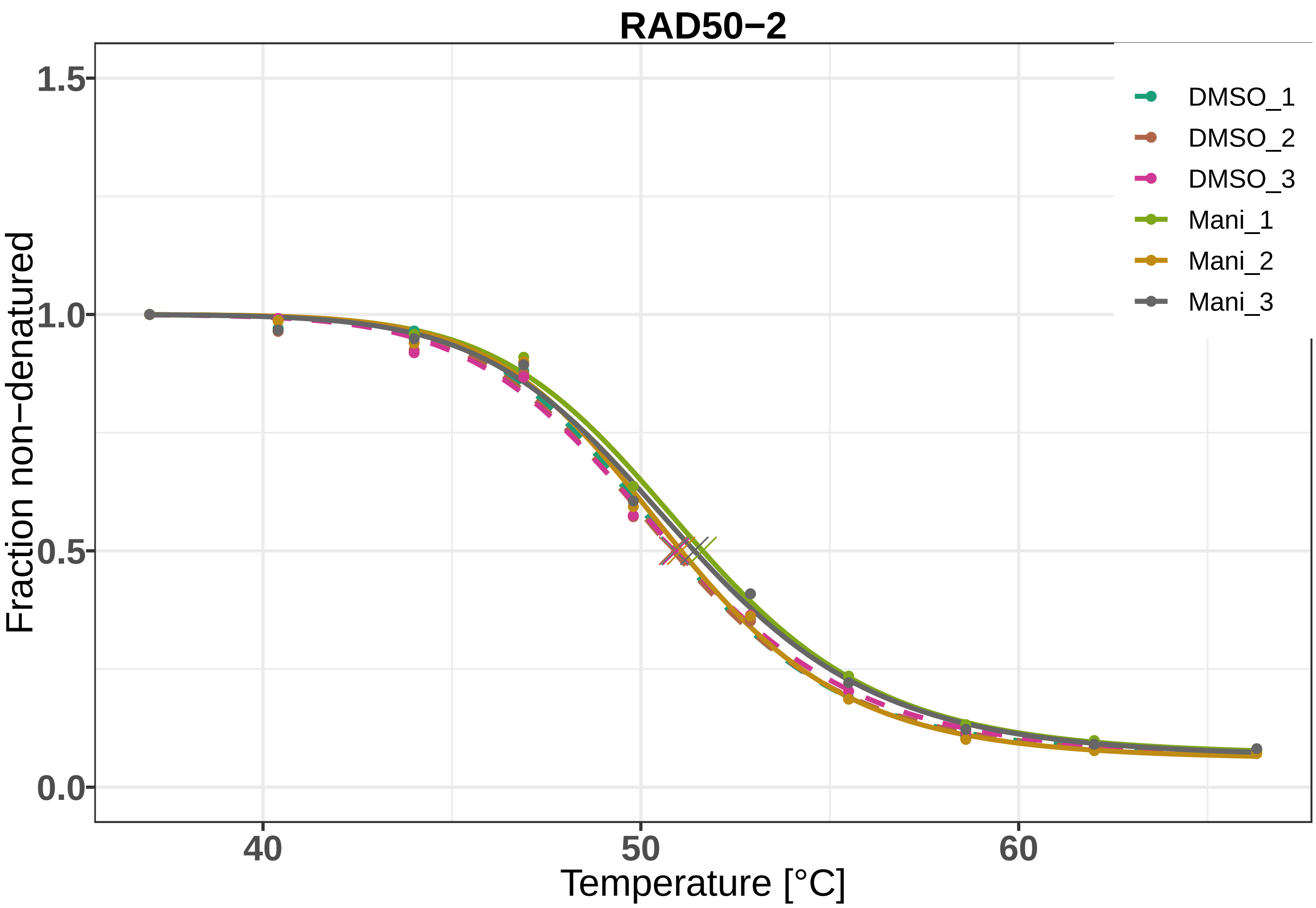

	meltPoint	slope	plateau	R2
DMSO_1	50.92	−0.099	0.07	1
DMSO_2	50.85	−0.095	0.06	1
DMSO_3	50.94	−0.09	0.06	1
Mani_1	51.63	−0.092	0.07	1
Mani_2	51.07	−0.1	0.06	1
Mani_3	51.42	−0.09	0.06	1

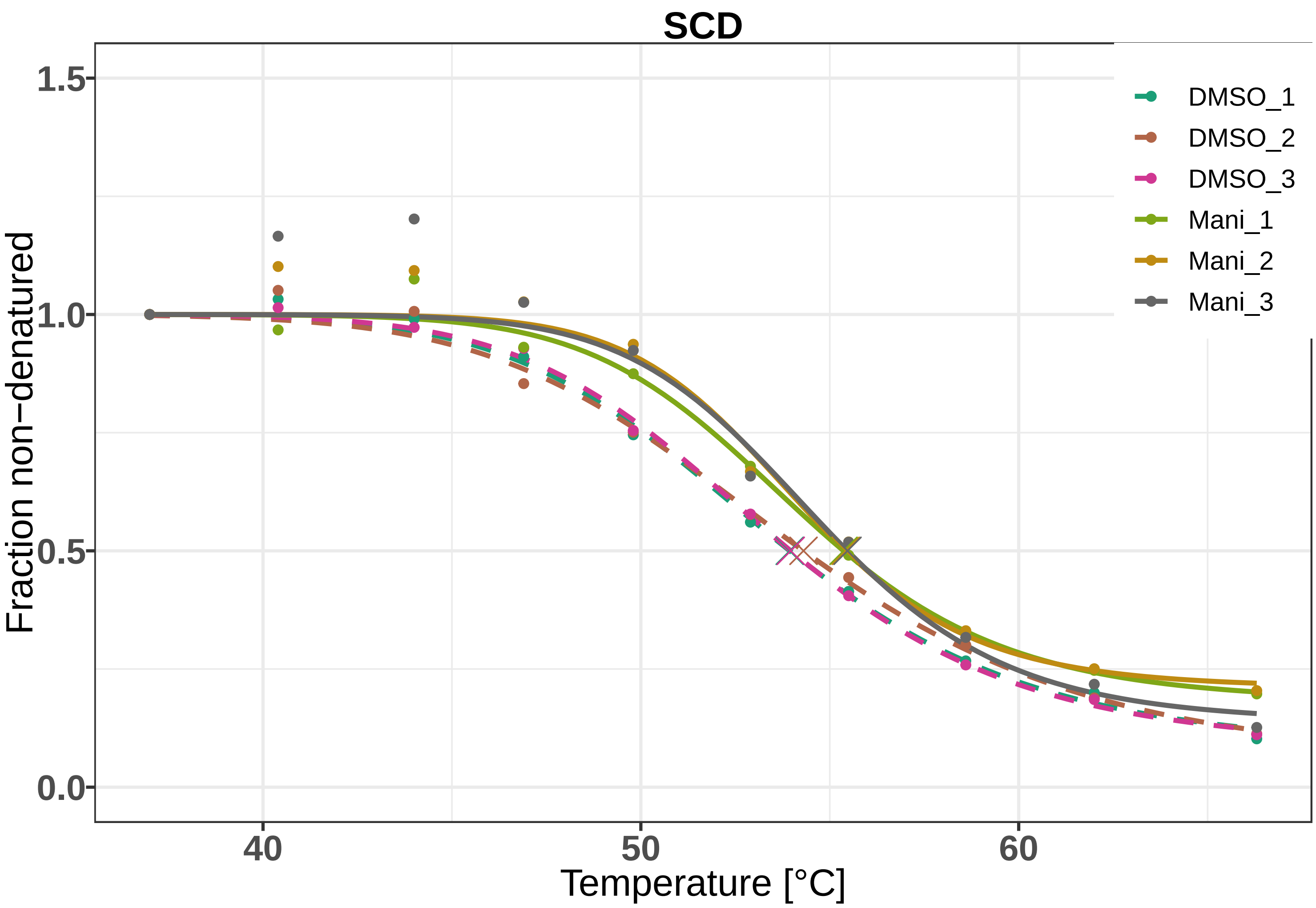

	meltPoint	slope	plateau	R2
DMSO_1	53.94	−0.067	0.08	1
DMSO_2	54.31	−0.06	0.05	0.99
DMSO_3	53.97	−0.069	0.08	1
Mani_1	55.37	−0.075	0.18	0.99
Mani_2	55.42	−0.088	0.21	0.98
Mani_3	55.47	−0.086	0.14	0.95

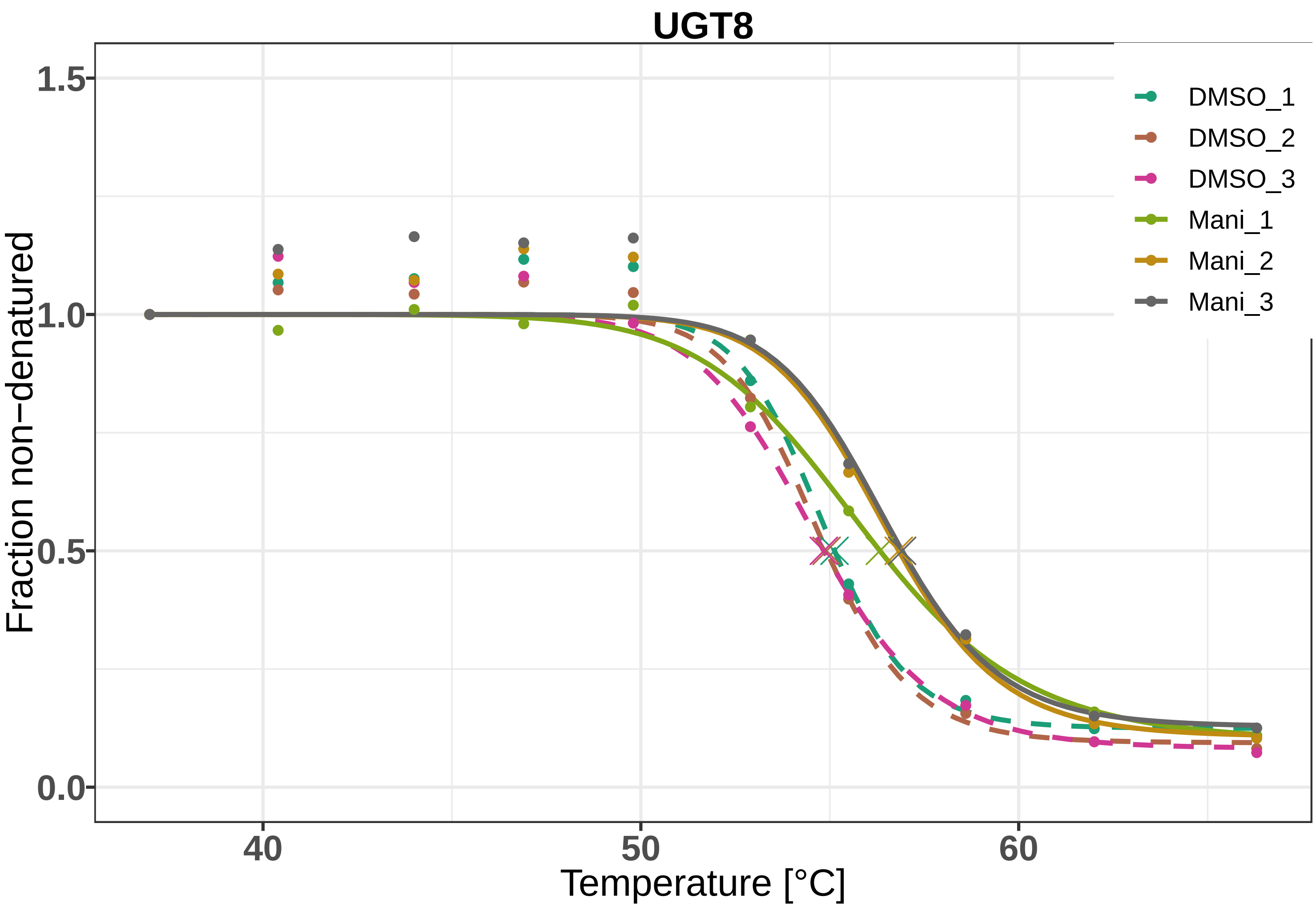

	meltPoint	slope	plateau	R2
DMSO_1	55.13	−0.19	0.12	0.98
DMSO_2	54.92	−0.18	0.09	0.99
DMSO_3	54.84	−0.15	0.08	0.98
Mani_1	56.33	−0.1	0.1	1
Mani_2	56.83	−0.15	0.11	0.97
Mani_3	56.91	−0.15	0.13	0.94

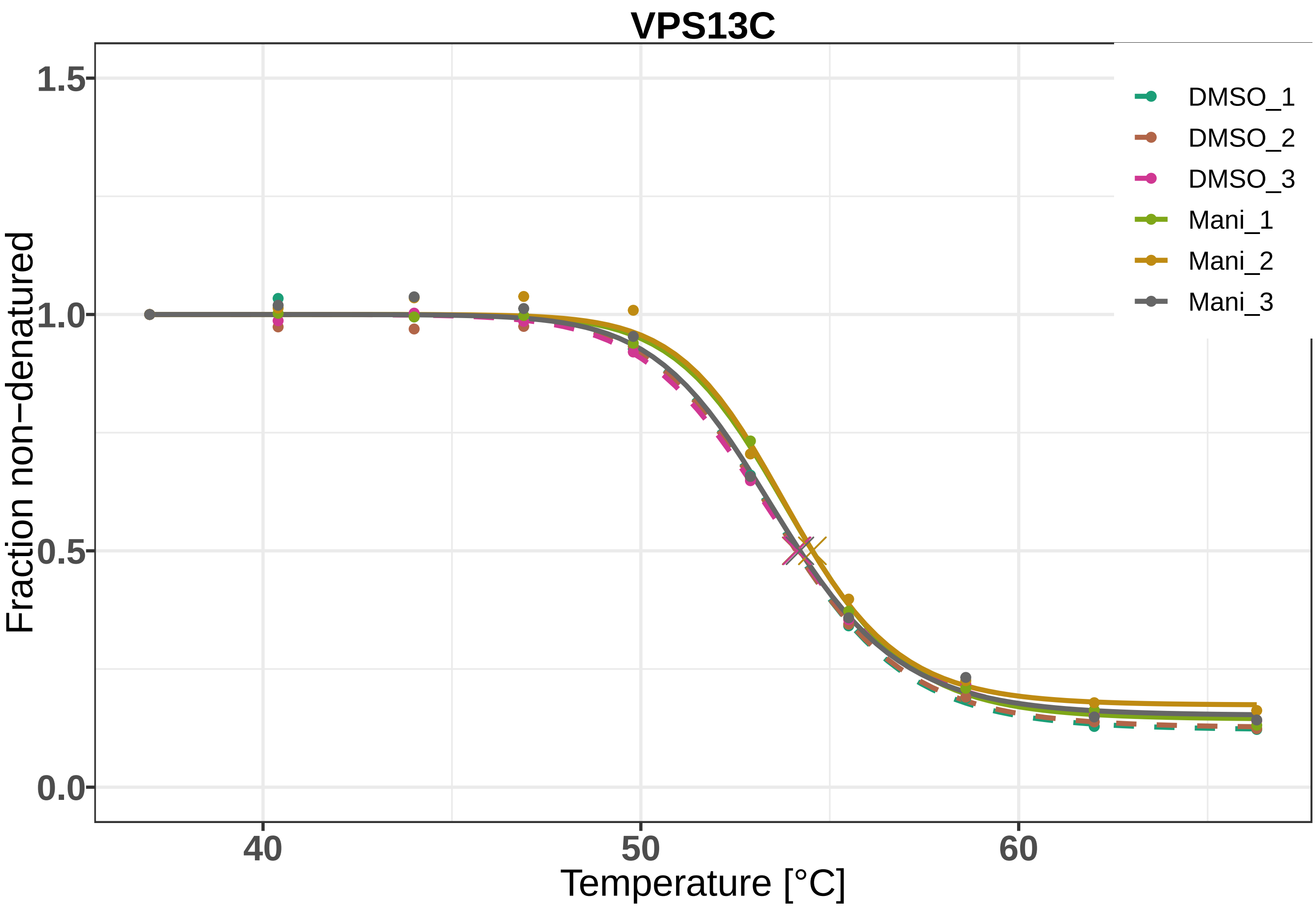

	meltPoint	slope	plateau	R2
DMSO_1	54.12	−0.13	0.12	1
DMSO_2	54.11	−0.13	0.13	1
DMSO_3	54.14	−0.12	0.14	1
Mani_1	54.54	−0.14	0.14	1
Mani_2	54.54	−0.14	0.17	1
Mani_3	54.21	−0.13	0.15	1
