# Supplementary material for: UGT8 mediated sulfatide synthesis modulates BAX localization and dictates apoptosis sensitivity of colorectal cancer
Source: Cell Death Differ. 2024 Nov 23;32(4):657–71. doi: 10.1038/s41418-024-01418-y (PMC11982410; doi:10.1038/s41418-024-01418-y)

Digitionine cytosolic fraction cyto c assay (Manidpine, A-1155463) (Figure 4f)

Cytosolic fraction

Cytochrome c 14kD

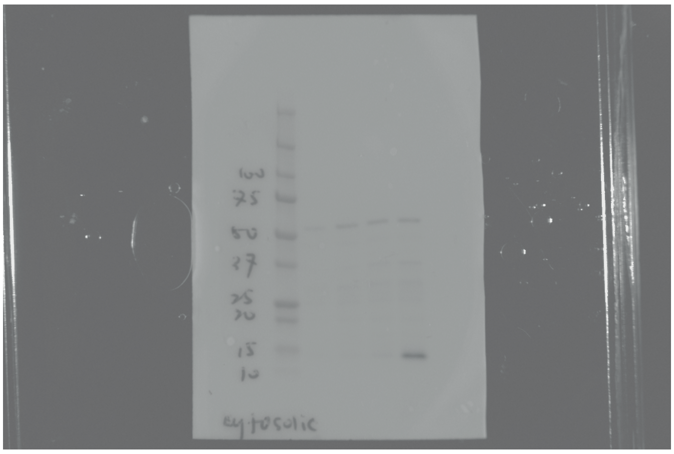

GAPDH 36kD

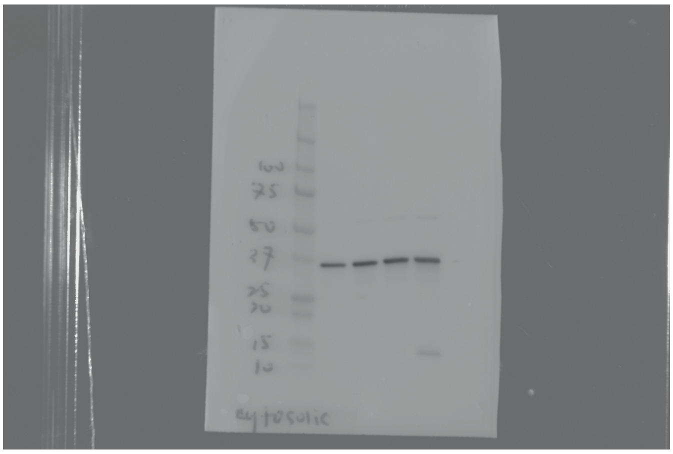

Fraction containing mitochondria

Cytochrome c 14kD

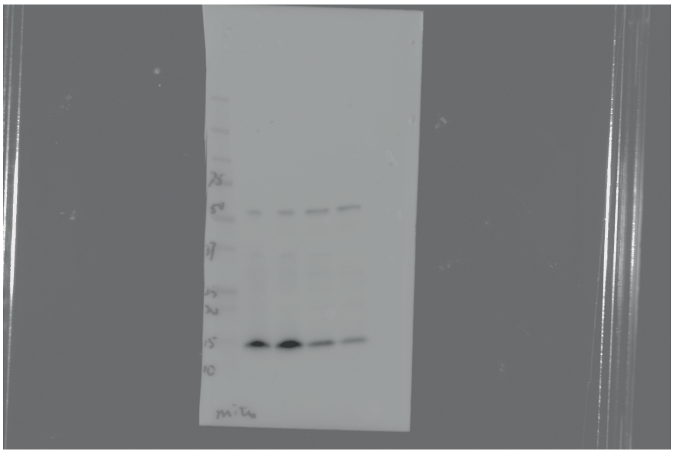

HSP60 60kD

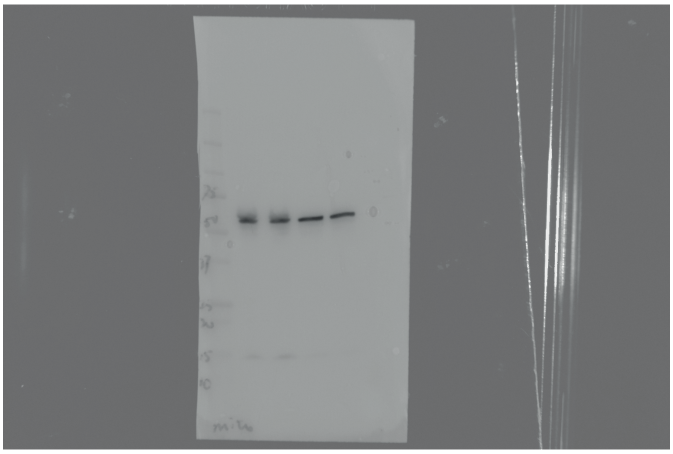

Digitionine cytosolic fraction cyto c assay (Manidpine, A-1155463, Sulfatides) (Figure 5g)

Cytosolic fraction

Cytochrome c 14kD

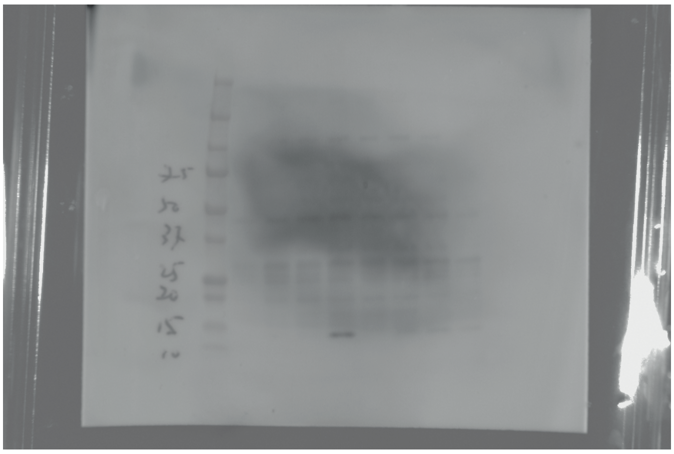

GAPDH 36kD

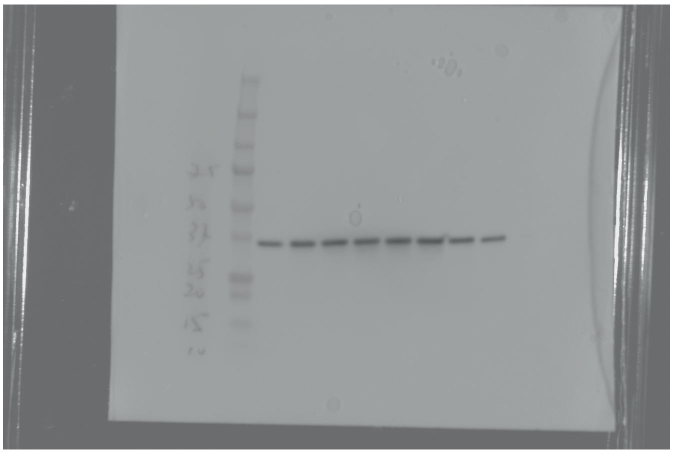

Fraction containing mitochondria

Cytochrome c 14kD

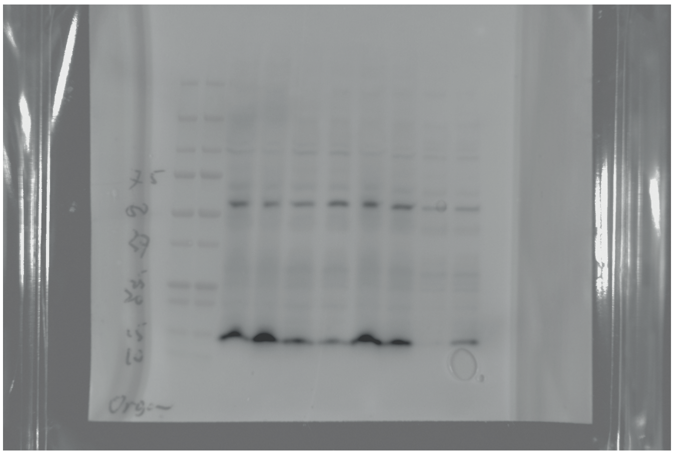

HSP60 60kD

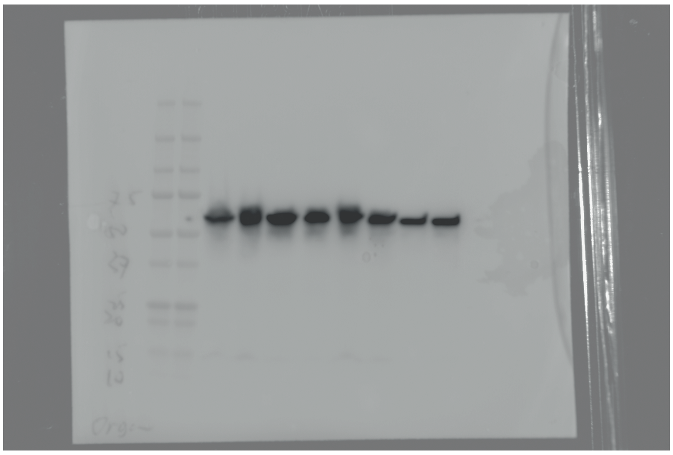

Isolated mitochondria BAX A-1155463 Sulfatides (Figure 5h)

Supernatants

Pellets

Cytochrome c
14kD

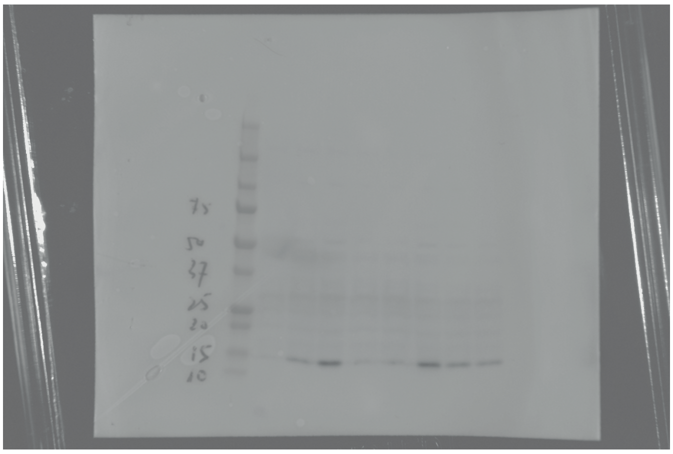

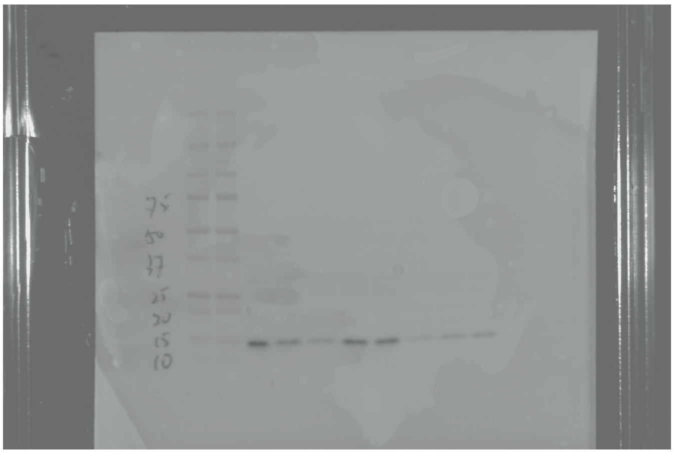

BAX and BAK KO validation (Extended Figure 2a)

BAX 20kD

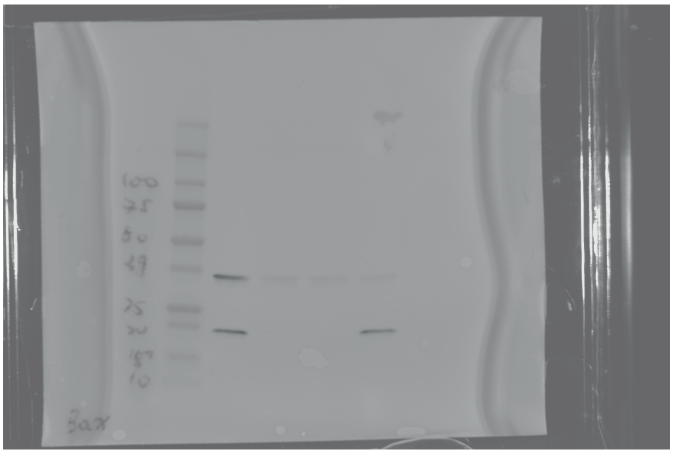

GAPDH 36kD

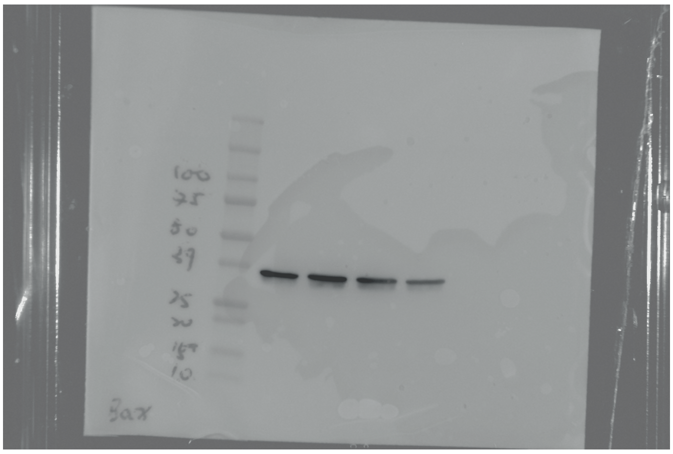

BAK 25kD

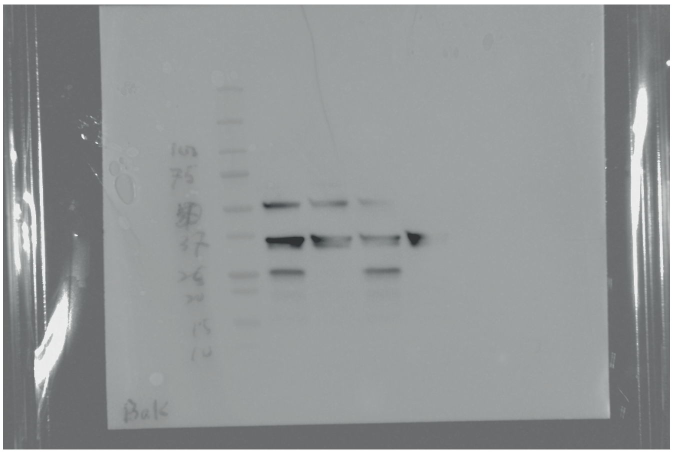

GAPDH 36kD

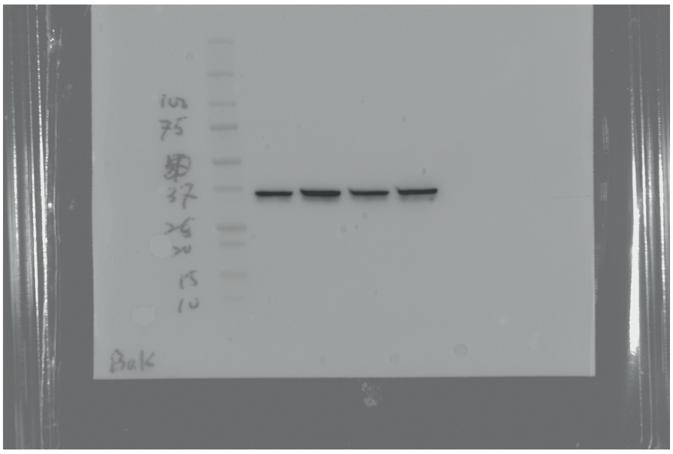

Mitochondria isolation validation (Extended Figure 3e)

HSP60 60kD

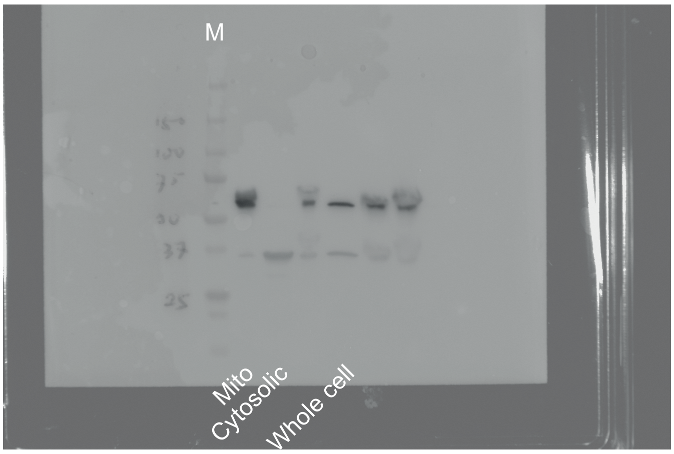

SERCA2 110kD

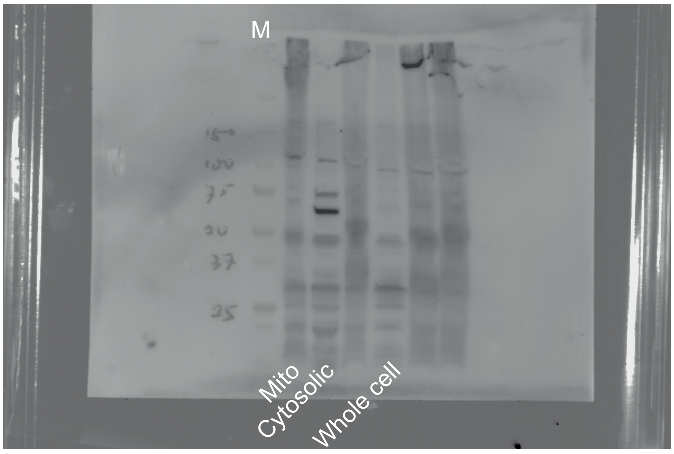

GAPDH 36kD

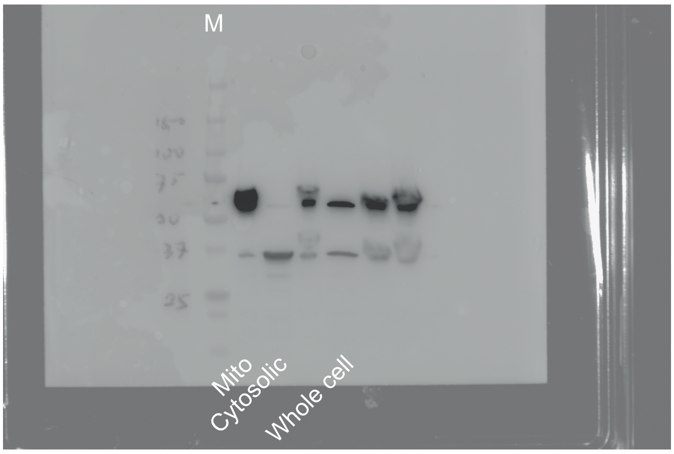

CFP-BAX Validation (Extended Figure 4a)

BAX

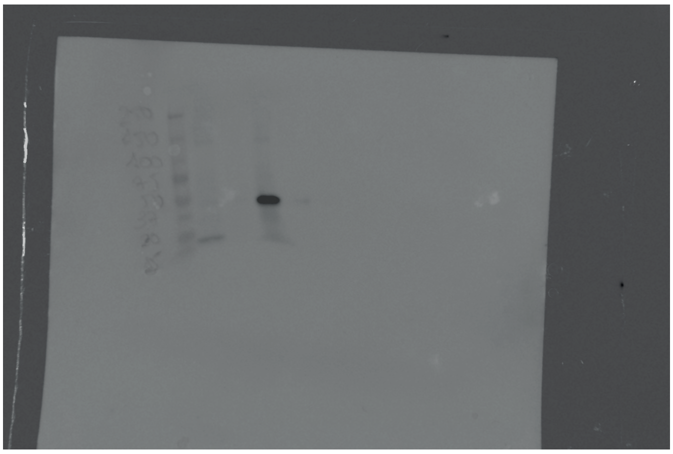

GAPDH

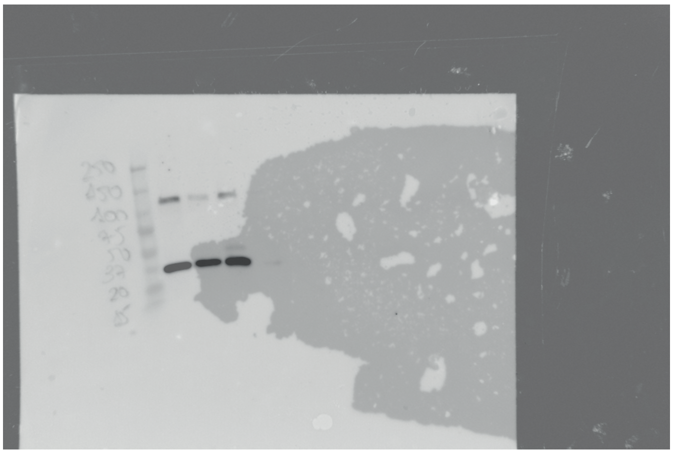

Supplement: Supplementary file 10 — Original WBlots [file 41418_2024_1418_MOESM10_ESM.pdf]
